# The Metabolic Fingerprint of Doxorubicin-Induced Cardiotoxicity in Male CD-1 Mice Fades Away with Time While Autophagy Increases

**DOI:** 10.3390/ph16111613

**Published:** 2023-11-15

**Authors:** Sofia Reis Brandão, Ana Reis-Mendes, Maria João Neuparth, Félix Carvalho, Rita Ferreira, Vera Marisa Costa

**Affiliations:** 1 Associate Laboratory i4HB—Institute for Health and Bioeconomy, Faculty of Pharmacy, University of Porto, 4050-313 Porto, Portugal; sofiarbrandao@ua.pt (S.R.B.); afreis.mendes@gmail.com (A.R.-M.); felixdc@ff.up.pt (F.C.); 2 UCIBIO-REQUIMTE, Laboratory of Toxicology, Department of Biological Sciences, Faculty of Pharmacy, University of Porto, 4050-313 Porto, Portugal; 3 LAQV-REQUIMTE, Department of Chemistry, University of Aveiro, 3810-193 Aveiro, Portugal; ritaferreira@ua.pt; 4 Laboratory for Integrative and Translational Research in Population Health (ITR), Research Centre in Physical Activity, Health and Leisure (CIAFEL), Faculty of Sports, University of Porto, 4200-450 Porto, Portugal; mjoao.neuparth@ipsn.cespu.pt; 5 TOXRUN—Toxicology Research Unit, University Institute of Health Sciences, CESPU, 4585-116 Gandra, Portugal

**Keywords:** cardio-oncology, doxorubicin, energetic metabolism, apoptosis, autophagy

## Abstract

The cardiotoxicity of doxorubicin (DOX) may manifest at the beginning/during treatment or years after, compromising patients’ quality of life. We intended to study the cardiac pathways one week (short-term, control 1 [CTRL1] and DOX1 groups) or five months (long-term, CTRL2 and DOX2 groups) after DOX administration in adult male CD-1 mice. Control groups were given saline, and DOX groups received a 9.0 mg/Kg cumulative dose. In the short-term, DOX decreased the content of AMP-activated protein kinase (AMPK) while the electron transfer flavoprotein-ubiquinone oxidoreductase (ETF-QO) increased compared to CTRL1, suggesting the upregulation of fatty acids oxidation. Moreover, mitofusin1 (Mfn1) content was decreased in DOX1, highlighting decreased mitochondrial fusion. In addition, increased B-cell lymphoma-2 associated X-protein (BAX) content in DOX1 pointed to the upregulation of apoptosis. Conversely, in the long-term, DOX decreased the citrate synthase (CS) activity and the content of Beclin1 and autophagy protein 5 (ATG5) compared to CTRL2, suggesting decreased mitochondrial density and autophagy. Our study demonstrates that molecular mechanisms elicited by DOX are modulated at different extents over time, supporting the differences on clinic cardiotoxic manifestations with time. Moreover, even five months after DOX administration, meaningful heart molecular changes occurred, reinforcing the need for the continuous cardiac monitoring of patients and determination of earlier biomarkers before clinical cardiotoxicity is set.

## 1. Introduction

The life span of cancer patients has increased considerably in recent decades due to better cancer therapies and earlier diagnosis [1]. Chemotherapeutic agents such as doxorubicin (DOX) are still among the most used cancer treatments. DOX is a topoisomerase II (TOP2) inhibitor given in the treatment of solid tumors and hematological malignancies [2]. Despite its efficacy against cancer, DOX has been associated with severe adverse side effects in non-target organs, mainly in the heart [2,3,4]. In fact, the cardiotoxicity of anticancer drugs is an emergent field known as cardio-oncology, with DOX being one of the most studied drugs.

The acute/subacute cardiotoxicity of DOX might manifest itself at the beginning of the treatment (up to two weeks after it ended), being rare and not dose-related. These manifestations include arrhythmias, acute coronary syndromes, acute heart failure, or pericarditis/myocarditis [2,5]. On the other hand, chronic cardiotoxicity is dose-related and may happen within one year or more after treatment. The early-onset chronic cardiotoxicity (within one year after treatment) has been associated with myocyte damage or death [5]. In contrast, the late-onset form may remain asymptomatic for several years [5,6]. This asymptomatic phase occurs because compensatory mechanisms such as adaptive myocardial hypertrophy are activated, allowing proper cardiovascular function for some time. However, when the compensatory mechanisms are overwhelmed, the heart function becomes compromised and the patients may develop cardiac symptomatic manifestations. Those manifestations may go from decreased left ventricular ejection fraction, or be irreversible like heart failure, where the therapeutic approaches are limited [5,6]. It was estimated that more than 50% of treated patients present left ventricular dysfunction 10 to 20 years after DOX administration and 5% of those patients would develop heart failure symptoms, half of whom would die [7,8]. Although heart failure, may represent a late manifestation of cardiotoxic anticancer drugs like DOX with poor diagnosis and high mortality, the mechanisms underlying the chronic cardiotoxicity of anticancer drugs are faintly represented in cardio-oncology preclinical studies [9].

While the cardiotoxicity associated with DOX is usually linked to the formation of reactive oxygen species (ROS) because of its molecular properties that favor redox cycling, this dogma has been challenged in recent years, since several adverse outcome pathways (AOPs) have been identified regarding DOX-induced cardiotoxicity. DOX elicits oxidative stress, lipid peroxidation, protein modification, DNA and mitochondrial damage, cardiomyocyte apoptosis, and disturbance of calcium and iron homeostasis that could be linked to the oxidative stress-induced hypothesis [9,10,11,12,13]. Nonetheless, new molecular mechanisms have been proposed, such as signaling pathways dependent on or independent of cardiac TOP2 β inhibition [9,14,15,16,17]. Regardless of the underlying mechanisms of anticancer drugs, cardiotoxicity is the second cause of death among cancer survivors (in some ages, cardiovascular mortality exceeds or equals cancer mortality) [18]. It is a known fact that cancer patients, upon remission, have their follow-up mostly limited to cancer recurrence, thus early signs of cardiotoxicity are easily neglected, leading to a spiral of irreversible cardiac damage, whose mechanisms are still under investigation.

Thus, herein, we proposed to evaluate the underlying cardiac molecular mechanisms modulated after DOX exposure at two different time-points. Hence, adult male CD-1 mice received multiple administrations of DOX to attain a total cumulative dose of 9 mg/kg, which is a clinically relevant total dose. Afterwards, mice were sacrificed one week (first time-point) or five months (second time-point) after the last administration to compare the early and late cardiac modulation of the most widely used chemotherapeutic agent, DOX. The molecular pathways searched are modulated at different extents over time after DOX administration, supporting cardiac adaptation. This study can further explain the differences in clinic cardiotoxic manifestations seen during the treatment and years after its termination.

## 2. Results

### 2.1. Doxorubicin Treatment Decreased Whole-Body and Heart Weight Five Months after Exposure

The four groups started with nine animals; however, two mice of the DOX2 group died one week and four weeks after the last intraperitoneal (i.p.) injection, respectively. The post-mortem examination of these animals found pleural effusions and a dilated myocardium, and therefore they were not considered for the following analysis.

No differences were found in the first time-point of DOX exposure (DOX1 vs. CTRL1 animals) for the morphometric parameters assessed (Table 1). On the other hand, the DOX2 group presented decreased whole-body and heart weight, as well as tibial length compared to their respective control (CTRL2). Nonetheless, no differences were found for the heart weight-to-whole-body weight and heart weight-to-tibial length ratios.

### 2.2. Doxorubicin Treatment Induced a Tendency for Decreased Albumin Concentration One Week after Exposure, and a Tendency for Decreased Content of C Reactive Protein (CRP) Five Months after Exposure on Serum

**Figure 1:**
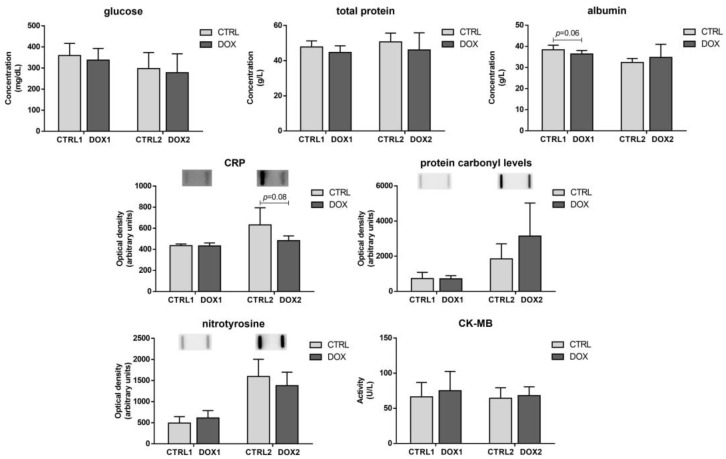
Long-term vs. short-term effects after DOX on blood serum. The concentration of glucose, total protein, and albumin, along with the content of C reactive protein (CRP), protein carbonyl levels, and nitrotyrosine, as well as the activity of creatine kinase-MB (CK-MB) are depicted. A representative image of the slot-blot obtained is presented above the corresponding graph. For the complete blot obtained, see Supplementary Figure S1. Values are expressed as mean ± SD (*n =* 9 for CTRL1 and CTRL2 and *n =* 7 for DOX1 and DOX2 for glucose, total protein, albumin, and CK-MB; *n =* 5–6 for CRP, protein carbonyl levels, and nitrotyrosine). Statistical analyses were carried out using unpaired two-sided *t*-test (*p* < 0.05).

No differences were found when comparing DOX1 and CTRL1 groups for the concentration of glucose and total protein (Figure 1). Nonetheless, a tendency towards decreased albumin concentration was found for the DOX1 group compared to CTRL1 (*p* = 0.06), but no differences in the content of CRP, protein carbonyl levels and nitrotyrosine were observed. When comparing DOX2 and CTRL2 groups, a trend towards decreased CRP content was found (*p* = 0.08). For the other serum systemic parameters assessed, no differences were found between DOX2 and CTRL2. Moreover, no impact was observed in the creatine kinase MB (CK-MB) activity, regardless of the time-point, indicating that no big differences occur in serum markers, independently of the time after DOX exposure.

**Figure 2:**
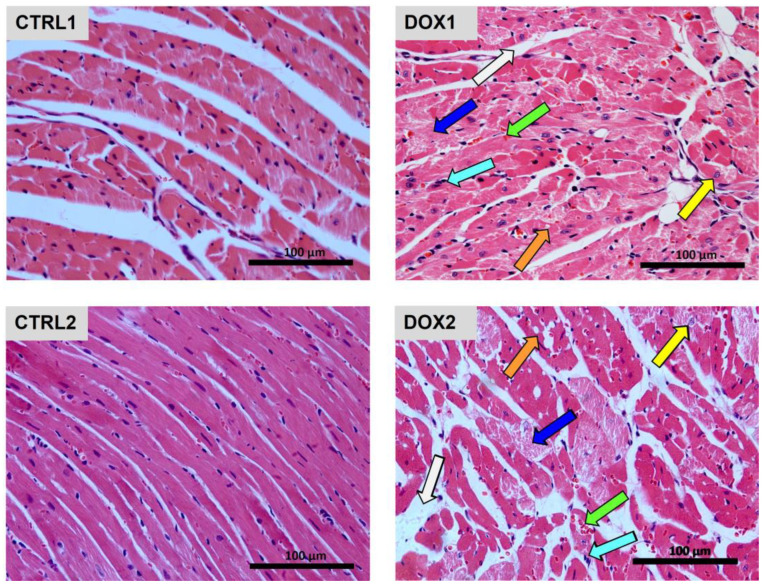
Long-term vs. short-term effects after DOX on the cardiac structure assessed by light microscopy. Representative light microscope micrographs obtained from three animals per group. Mice controls of each time-point (CTRL1 and CTRL2) were compared to DOX-treated animals (DOX1 and DOX2). DOX elicited, in both time-points, cardiac interstitial edema (white arrow), necrotic zones (blue arrow), vascular congestion (green arrow), inflammatory infiltration (cyan arrow), vacuolization (orange arrow), along with large and uncondensed nuclei (yellow arrow). Images were taken at 40× magnification (scale bar = 100 µm).

Additionally, the heart histological assessment showed increased tissue damage after DOX (Figure 2). When considering one week after exposure, DOX1 animals presented large and uncondensed nuclei, as well as vacuolization of the cytoplasm, along with necrosis, vascular congestion, interstitial inflammatory cell infiltration, and edema. Likewise, DOX2 animals displayed interstitial inflammatory cell infiltration and edema, with vascular congestion, but seemed to present more necrotic zones than DOX1. Moreover, DOX2 animals presented cytoplasm vacuolization and large and uncondensed nuclei. For more detailed information and quantification regarding the histological assessment, consult the following paper [19].

### 2.3. Doxorubicin Treatment Decreased the Cardiac Content of the Metabolic Regulator AMP-Activated Protein Kinase (AMPK), and Increased the Cardiac Content of Electron Transfer Flavoprotein-Ubiquinone Oxidoreductase (ETF-QO) One Week after Exposure

**Figure 3:**
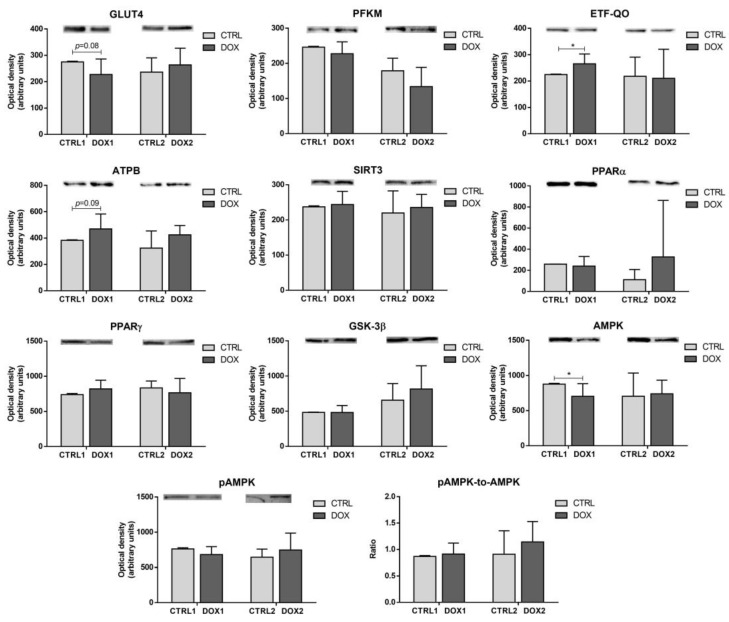
Long-term vs. short-term effects after DOX on heart metabolism and its regulation. The optical density of glucose transporter GLUT4 (GLUT4), phosphofructokinase (PFKM), electron transfer flavoprotein-ubiquinone oxidoreductase (ETF-QO), ATP synthase subunit β (ATPB), sirtuin 3 (SIRT3), peroxisome proliferator-activated receptors α (PPARα) and γ (PPARγ), glycogen synthase kinase 3 β (GSK-3β), AMP-activated protein kinase (AMPK), and phosphorylated AMPK (pAMPK) are depicted along with the pAMPK-to-AMPK ratio. A representative image of the Western blot obtained is presented above each graph. For the complete blot obtained, see Supplementary Figure S1. Values are expressed as mean ± SD (*n =* 5–6). Statistical analyses were performed using unpaired two-sided *t*-test: * *p* < 0.05.

The content of glucose transporter GLUT4 (GLUT4), which allows the import of glucose into cardiac cells [20], showed a trend to be decreased in the DOX1 group compared to CTRL1 (*p* = 0.08, Figure 3), probably indicating a reduced uptake of glucose. The content of the glycolytic enzyme phosphofructokinase (PFKM), responsible for the irreversible phosphorylation of fructose 6-phosphate to fructose 1,6-bisphosphate [21], was not changed by DOX administration either at the first or second time-points, evidencing no influence of DOX on glycolysis over time. On the other hand, the cardiac content of ETF-QO was increased in the DOX1 group compared to CTRL1, suggesting increased fatty acids oxidation (FAO), since ETF-QO acts in a coordinated way with electron transfer flavoproteins in the transfer of electrons from FAO enzymes to ubiquinone at oxidative phosphorylation (OXPHOS) [22]. Moreover, the content of ATP synthase subunit β (ATPB), which is an essential subunit of C-V of OXPHOS [23], showed a tendency to be increased in the DOX1 compared to CTRL1 (*p* = 0.09), pointing to a rise in oxidative metabolism for the first time-point, which was not seen longer after the exposure. Nonetheless, the content of modulators of these metabolic proteins, and respective energetic pathways, was not altered by DOX treatment, regardless of the time-point selected: sirtuin 3 (SIRT3), peroxisome proliferator-activated receptors α (PPARα) and γ (PPARγ), along with glycogen synthase kinase 3 β (GSK-3β). Additionally, AMPK content was decreased in DOX1 compared to CTRL1, despite no differences being found for either the content of phosphorylated AMPK (pAMPK) nor for the pAMPK-to-AMPK ratio, suggesting a higher effect of DOX on this metabolic modulator when considering one week after the exposure. This data emphasizes metabolic adaptation over time.

### 2.4. Doxorubicin Treatment Induced a Tendency for Decreased Cardiac Content of Manganese Superoxide Dismutase (MnSOD) Five Months after Exposure

**Figure 4:**
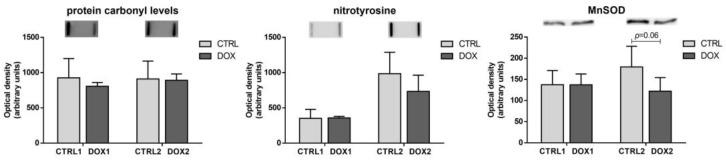
Long-term vs. short-term effects after DOX on heart redox/oxidative stress status. The optical density of protein carbonyl levels, nitrotyrosine, and manganese superoxide dismutase (MnSOD) are depicted. A representative image of the immuno-blot obtained is presented above each graph. For the complete blot obtained, see Supplementary Figure S1. Values are expressed as mean ± SD (*n =* 5–6). Statistical analyses were performed using unpaired two-sided *t*-test (*p* < 0.05).

No differences were found for the cardiac content of carbonylated and nitrated proteins, given by protein-2,4-dinitrophenyl hydrazone moiety and nitrotyrosine levels, either at the first or the second time-points of DOX exposure (Figure 4). Nevertheless, a trend towards decreased MnSOD content was found for the DOX2 group compared to CTRL2 (*p* = 0.06), probably indicating more impact of DOX over time on the redox/oxidative mitochondrial status.

### 2.5. Doxorubicin Treatment Induced a Tendency for Increased Cardiac Mitochondrial Density One Week after Exposure, While Decreasing It Five Months after Exposure

**Figure 5:**
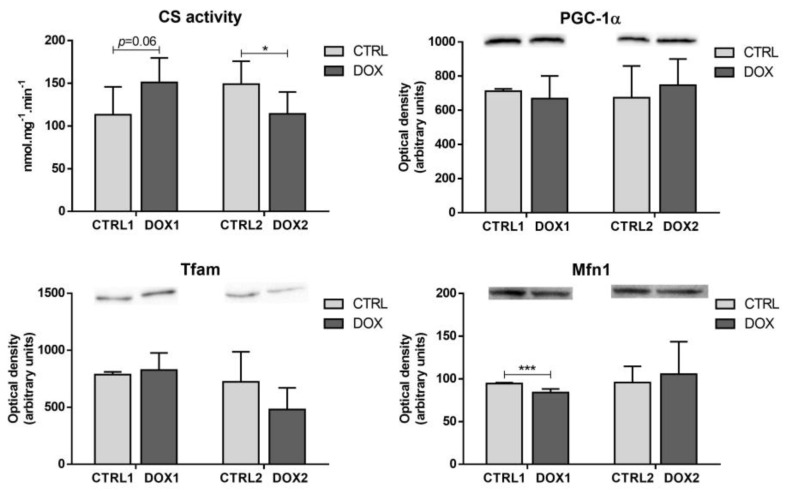
Long-term vs. short-term effects after DOX on heart mitochondrial density and biogenesis. Citrate synthase (CS) activity was determined using a spectrophotometric assay. The optical density of peroxisome proliferator-activated receptor γ coactivator 1 α (PGC-1α), mitochondrial transcription factor A (Tfam) and mitofusin1 (Mfn1) are depicted. A representative image of the Western blot obtained is presented above each graph. For the complete blot obtained, see Supplementary Figure S1. Values are expressed as mean ± SD (*n =* 5–6). Statistical analyses were performed using unpaired two-sided *t*-test: * *p* < 0.05, *** *p* < 0.001.

DOX1 group showed a tendency to increase the citrate synthase (CS) activity compared to CTRL1 (*p* = 0.06, Figure 5), with this measurement being considered a rough marker of mitochondrial density [24]. Additionally, the DOX1 group exhibited decreased mitofusin1 (Mfn1) content compared to CTRL1. This highlights its influence on mitochondrial density, since Mfn1 acts specifically in mitochondrial fusion, leading to elongated interconnected networks [25]. DOX2 animals presented a decrease in the activity of CS compared to CTRL2 ones, suggesting decreased mitochondrial density and cardiac mitochondrial adaptation with time. However, no differences were found for the content of the transcriptional coactivator, peroxisome proliferator-activated receptor γ coactivator 1 α (PGC-1α), and one of its mitochondrial targets, mitochondrial transcriptional factor A (Tfam) [26], after DOX exposure, regardless of the time-point selected.

### 2.6. Doxorubicin Treatment Increased Cardiac Apoptosis One Week after Exposure, While Decreasing Cardiac Autophagic Markers and Increasing Heat Shock Protein 27 (HSP27) Content Five Months after Exposure

**Figure 6:**
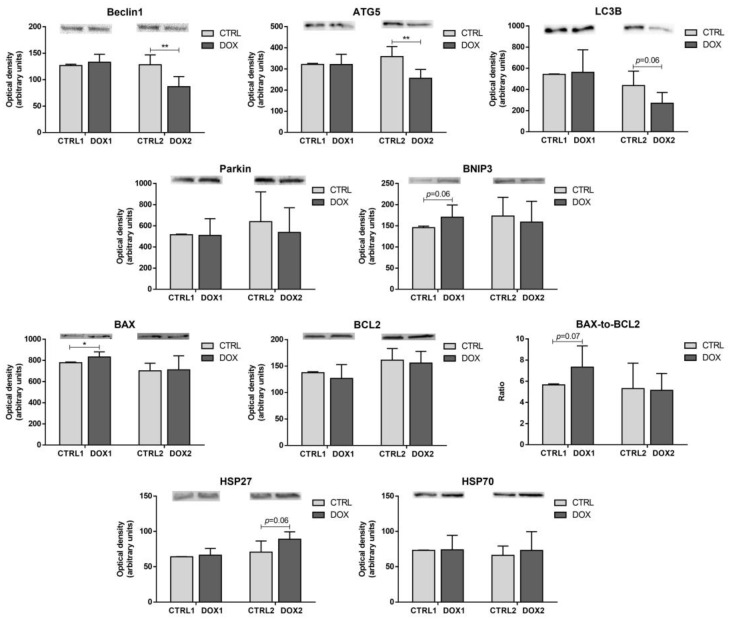
Long-term vs. short-term effects after DOX on heart autophagy and apoptosis. The optical density of Beclin1, autophagy protein 5 (ATG5), microtubule-associated protein 1 light chain 3 β (LC3B), Parkin, B-cell lymphoma-2 interacting protein 3 (BNIP3), B-cell lymphoma-2 associated X-protein (BAX), B-cell lymphoma-2 (BCL2), heat shock proteins 27 (HSP27) and 70 (HSP70) are depicted along with the BAX-to-BCL2 ratio. A representative image of the Western blot obtained is presented above each graph. For the complete blot obtained, see Supplementary Figure S1. Values are expressed as mean ± SD (*n =* 5–6). Statistical analyses were performed using unpaired two-sided *t*-test: * *p* < 0.05, ** *p* < 0.01.

No differences were observed for the content of the autophagy markers assessed when comparing DOX1 and CTRL1 groups: Beclin1, autophagy protein 5 (ATG5), and microtubule-associated protein 1 light chain 3 β (LC3B, Figure 6). On the other hand, the DOX2 group decreased Beclin1 and ATG5 content compared to CTRL2, pointing to reduced autophagy five months after DOX exposure and, thus, suggesting cardiac adaptation over time. Moreover, the content of LC3B showed a trend to be decreased in DOX2 animals compared to CTRL2 (*p* = 0.06).

Additionally, and despite no differences for Parkin, a tendency towards increased content of B-cell lymphoma-2 interacting protein 3 (BNIP3) was observed in DOX1 group compared to CTRL1 (*p* = 0.06). Conversely, no differences were seen for these two proteins that act on the elimination of damaged mitochondria, also termed mitophagy [27,28], when considering the second time-point groups. These results point out to modulation of this protein at different extents according to the time that has passed after the DOX exposure.

Moreover, the content of the pro-apoptotic protein B-cell lymphoma-2 associated X-protein (BAX) was increased in the DOX1 group compared to CTRL1. Despite no significant changes in the anti-apoptotic protein B-cell lymphoma-2 (BCL2), the BAX-to-BCL2 ratio, which is indicative of increased mitochondria-mediated apoptosis pathway [29], showed a trend to be increased when comparing the first time-point groups. On the other hand, no differences were seen for these proteins when contemplating the second time-point groups. Nonetheless, the content of HSP27 was increased in the DOX2 group compared to CTRL2 (*p* = 0.05). The distinct modulation observed at the two time-points is suggestive of cardiac molecular adaptation over time after DOX administration. Additionally, no differences were seen for the content of heat shock protein 70 kDa (HSP70) among groups.

### 2.7. Doxorubicin Treatment Did Not Affect Cardiac Regeneration Either One Week or Five Months after Exposure

**Figure 7:**
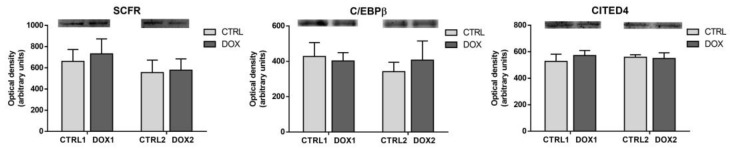
Long-term vs. short-term effects after DOX on heart regeneration. The optical density of mast/stem cell growth factor receptor Kit (SCFR), CCAAT/enhancer-binding protein β (C/EBPβ) and Cbp/p300-interacting transactivator 4 (CITED4) are depicted. A representative image of the Western blot obtained is presented above each graph. For the complete blot obtained, see Supplementary Figure S1. Values are expressed as mean ± SD (*n =* 5–6). Statistical analyses were performed using unpaired two-sided *t*-test (*p* < 0.05).

No differences were found for the content of cardiac regeneration markers assessed, mast/stem cell growth factor receptor Kit (SCFR), CCAAT/enhancer-binding protein β (C/EBPβ), and Cbp/p300-interacting transactivator 4 (CITED4), either at first or second time-points of DOX exposure (Figure 7). SCFR initiates several proliferative and survival cascades on cardiac progenitor cells (CPCs) [30]. C/EBPβ acts as a coactivator of cellular proliferation on cardiomyocytes [31], and it may also inhibit CITED4, an inhibitor of apoptosis and autophagy [32]. Thus, these results suggest no impact of this pathway on DOX-induced cardiac muscle remodeling.

## 3. Discussion

Cardio-oncology is a clinical and scientific topic with a myriad of unanswered questions. DOX was identified as a cardiotoxic drug as soon as its application on clinical assays began [3,4], but even nowadays its underlying mechanisms are not completely elucidated. When addressing chronic cardiotoxicity, the studies are scarce, and that paradigm needs to be further scrutinized to determine new patterns of post-cancer follow up. Our work aims to contribute to this theme in an integrated fashion, where two time-points were evaluated in parallel to address the DOX-induced cardiotoxicity in a preclinical model. Adult mice of approximately 12 weeks old (about 20–21 human years) received a clinically relevant dose of DOX (9 mg/kg) distributed over three weeks. One week after the last administration, corresponding roughly to seven human months, was the selected time-point to mimic the early-onset form of chronic cardiotoxicity and infer about the cardiac modulation at the short-term. The second time-point, five months after the treatment ended, corresponds to around 11 human years, and implied long-term modulation of cardiac tissue [33]. This time-point represents the late-onset form of chronic cardiotoxicity, which is associated with poorer prognosis. Moreover, the two time-points selected herein allowed the exploration of cardiac adaptation and AOPs changes over time.

Overall, no meaningful changes in whole-body and heart weight were found in the short-term after DOX exposure (one week, DOX1 vs. CTRL1). Moreover, no big differences were observed in the serum biochemical parameters. Indeed, only a tendency towards decreased albumin concentration was found for DOX1 compared to CTRL1, without other alterations on serum biomarkers. Albumin is the most abundant protein in the blood, being in charge of binding and transport of peptides, fatty acids and drugs, among other components, being also an inflammatory marker [34]. Thus, this finding for albumin concentration may suggest tendency to an increased inflammatory response, and DOX-induced cardiotoxic mechanisms were previously associated with increased inflammation [14,15,35], but we do not overrule other mechanisms. Indeed, histologic cardiac damage was observed after one week of DOX exposure, as seen and quantified in a previous work of the group [19], showing that serum biomarkers are not as sensitive as one could hope for.

**Figure 8:**
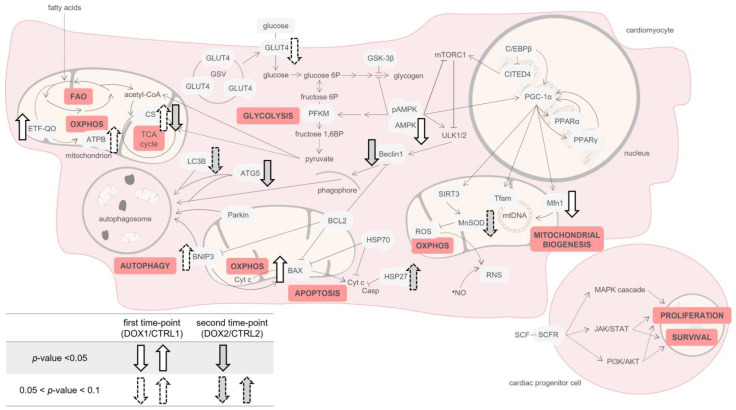
Proposed schematic representation of the molecular pathways modulated by DOX at first and second time-points selected (one week vs. five months after DOX exposure). The modulation considering the first time-point, DOX1/CTRL1, is represented in white, while grey indicates the second time-point groups: DOX2/CTRL2. Proteins found increased or decreased are indicated by up or down arrows, respectively. The predisposition of proteins (0.05 < *p*-value < 0.1) to be increased or decreased is indicated by dashed arrows. At a short-term period, metabolism and apoptosis were impacted, whereas long-term after DOX exposure mainly decreased autophagy. For summarized changes over time of all biomarkers assessed, see Supplementary Table S1. Figure partly made with Servier medical art, provided by Servier, licensed under a Creative Commons Attribution 3.0 unported license. 1,6BP: 1,6-bisphosphate, 6P: 6-phosphate, AMPK: AMP-activated protein kinase, ATG5: autophagy protein 5, ATPB: ATP synthase subunit β, BAX: B-cell lymphoma-2 associated X-protein, BCL2: B-cell lymphoma-2, BNIP3: BCL2 interacting protein 3, Casp: caspase, C/EBPβ: CCAAT/enhancer-binding protein β, CITED4: Cbp/p300-interacting transactivator 4, CS: citrate synthase, Cyt c: cytochrome c, ETF-QO: electron transfer flavoprotein-ubiquinone oxidoreductase, FAO: fatty acids oxidation, GLUT4: glucose transporter, GSK-3β: glycogen synthase kinase 3 β, GSV: GLUT4 storage vesicle, HSP: heat shock protein, JAK/STAT: Janus kinase/signal transducer and activator of transcription pathway, LC3B: microtubule-associated protein 1 light chain 3 β, MAPK: mitogen-activated protein kinase, Mfn1: mitofusin1, MnSOD: manganese superoxide dismutase, mtDNA: mitochondrial DNA, mTORC1: serine/threonine-protein kinase mTOR complex 1, •NO: radical nitric oxide, OXPHOS: oxidative phosphorylation, pAMPK: phosphorylated AMPK, PFKM: phosphofructokinase, PGC-1α: peroxisome proliferator-activated receptor γ coactivator 1 α, PI3K/AKT: phosphatidylinositol-3-kinase/protein kinase B, PPAR: peroxisome proliferator-activated receptor, RNS: reactive nitrogen species, ROS: reactive oxygen species, SCF: stem cell factor, SCFR: mast/stem cell growth factor receptor Kit, SIRT3: sirtuin 3, TCA: tricarboxylic acid, Tfam: mitochondrial transcription factor A, ULK1/2: complex of serine/threonine-protein kinases ULK1 and ULK2.

Regarding the findings on cardiac muscle, a tendency towards decreased content of total GLUT4 happened when comparing DOX1 with CTRL1. GLUT4 is the main glucose transporter in cardiomyocytes and is located in intracellular GLUT4 storage vesicles (GSVs, Figure 8) [20]. Decreased GLUT4 levels may suggest less transport of glucose into cardiomyocytes. Indeed, considering that the heart muscle uses the available circulating supplies and that neither changes in circulating glucose levels or in the content of cardiac PFKM occurred, it may hint that no big alterations in glucose uptake into cardiomyocytes or in glycolysis happened in the short-term after DOX exposure [21]. On the other hand, other studies have reported the upregulation of glycolysis after DOX, following higher cumulative doses [36,37,38]. In those studies, authors advocate it as being a consequence of the decline in FAO. However, in our study, the cardiac content of ETF-QO was increased in DOX1 compared to CTRL1, suggesting increased FAO [22]. Similarly, the content of ATPB presented a trend towards increased values. Thus, it seems that FAO and OXPHOS, the main energetic pathways of heart muscle [22,23], were increased in the short-term after DOX treatment, while glycolysis suffered no meaningful changes. Nonetheless, the modulation observed for AMPK, a metabolic regulator of glucose and lipid oxidation [39], differs from the one observed for FAO when considering DOX1 and CTRL1 groups. Indeed, the content of AMPK was decreased, which may be linked to glucose metabolism and therefore to the decrease in GLUT4 content. Thus, it seems likely that other pathways may have enhanced FAO. Namely, PPARα and PPARγ pathways, which are also crucial in the metabolism of lipids and glucose on cardiomyocytes [40], may have interfered with the AMPK role. Moreover, AMPK downregulation was previously found in a human cardiac cell line exposed to 0.1 μM of DOX for 24 h, which also presented the upregulation of FAO enzymes. Although the authors did not discuss or gave any hypothesis regarding these facts [41], they evidenced the ability of DOX to modulate this energy outcome pathway.

Moreover, DOX1 showed a tendency to increase the CS activity compared to CTRL1, suggesting an increased mitochondrial density [24]. Mitochondrial density reflects the balance between biogenesis and clearance [42]. Nonetheless, herein no differences were found for PGC-1α or Tfam, suggesting no meaningful impact on mitochondrial biogenesis. Moreover, no alterations were found for the proteins under the transcriptional activation of PGC-1α including SIRT3, PPARα, and PPARγ that are essential to keep energy metabolism and mitochondria functionality [26,40]. On the other hand, DOX1 decreased the Mfn1 content compared to CTRL1 (Figure 8), highlighting its influence on mitochondrial dynamics. Mnf1 acts specifically in mitochondrial fusion, leading to elongated interconnected networks that allow the renewal of damaged mitochondrial DNA (mtDNA) and/or enable energy supply inside the cells. Mitochondria fusion and its opposite process, fission, are essential for mitochondria movement and morphology, and consequently to keep their health [25]. Thus, the decrease in Mfn1 suggests less fusion of mitochondria and eventually an elevated number of mitochondria that may be related to the increased tendency of CS activity. Indeed, a reduced amount of Mfn1 was associated with enhanced mitochondrial fission as a compensatory mechanism to eliminate dysfunctional mitochondria through mitophagy [25].

Additionally, a tendency to increase the content of BNIP3 was observed in DOX1 compared to CTRL1 (Figure 8). This protein locates in the outer mitochondrial membrane connecting with the autophagic protein LC3B and initiating the elimination of damaged mitochondria [28]. Although BNIP3 is often linked to mitophagy, no significant changes were seen for other related markers, namely Parkin and autophagy proteins (Beclin1, ATG5, and LC3B) when comparing DOX1 and CTRL1. Moreover, BNIP3 has been linked to other cardiac pathways. Indeed, it acts on apoptosis through stimulation of the pore formation by pro-apoptotic proteins, including BAX. This allows cytochrome c (Cyt c) release. BNIP3 also blocks the activation of BAX once it heterodimerizes with anti-apoptotic proteins, such as BCL2 [28]. In the present work, the content of BAX was increased, as well as the ratio BAX-to-BCL2, indicative of increased mitochondria-mediated apoptosis [29], suggesting an influence of short-term DOX exposure on the mitochondrial apoptotic pathway possible through BNIP3.

On the other hand, whole-body and heart weight, as well as tibial length, were found to decrease when a long time as passed after DOX treatment (five months, DOX2 vs. CTRL2), showing the influence of this low cumulative dose on animals’ well-being over time. Treatment with DOX was previously shown to decrease whole-body and heart weight [16,35,43], including in one study of our group where a similar preclinical model submitted to a double dose but with a shorter time after exposure was used [35]. The decline in whole-body weight was associated with gastrointestinal toxicity of DOX [35]. In the present work with low dosing, those effects are still persistent five months after the DOX exposure, despite no differences being seen at one week, implying adaptation to this anthracycline over time.

No significant differences were seen in the markers of whole-body metabolism, inflammation, and cardiac damage in serum. Still, a tendency to decreased levels of CRP was noted for DOX2 group compared to CTRL2, but no differences in the protein carbonyl levels or nitrotyrosine content were found in serum (Figure 8). Carbonyl levels and nitrotyrosine allow the observation of the consequences of exposure to ROS and reactive nitrogen species (RNS) in proteins [44]. These data suggest that DOX does not elicit oxidative stress in the long term after exposure. Actually, similar results were found in the short-term paradigm, which may be derived by the low (but clinically relevant) cumulative dose used herein (9 mg/kg). Nonetheless, histologically, heart damage was still evident even five months after DOX administration, as seen and quantified previously by our group [19], indicating the relevance of this dosage to impact the cardiac tissue, despite serum markers seeming to not be modulated at this stage.

Regarding energetic metabolism, no differences in any of the proteins evaluated were found in the long-term time-point after DOX treatment, contrasting with the short-term time-point. This implies that a cardiac metabolic adaptation occurred over time. Despite their role as metabolic powerhouses in heart muscle, mitochondria are also the main cardiac local of ROS production [44]. Herein, no differences in cardiac ROS and RNS levels were found based on the protein carbonyl levels and nitrotyrosine content. Nevertheless, the content of the mitochondrial enzyme MnSOD showed a tendency towards decreased values when comparing DOX2 with CTRL2 (Figure 8). This enzyme transforms the anion superoxide radical to hydrogen peroxide [44], but herein no differences in the protein carbonyl levels were found. MnSOD is located specifically on mitochondria, and we measured its expression on heart homogenates, therefore the predisposition to be decreased may be a consequence of mitochondria dynamics influenced by DOX at this dose scheme rather than a direct effect on redox/oxidative stress. Indeed, decreased MnSOD content along with other markers of oxidative damage was reported in cardiac muscle of male C57BL/6 mice administered with a single higher dose of DOX (15 mg/kg i.p.) [45]. Nonetheless, our findings indicate mitochondrial adaptation over time at this cumulative dose of DOX and possibly not a response related to oxidative stress.

The activity of CS was decreased in DOX2 compared to CTRL2, advocating for decreased mitochondrial density when a long-term period elapsed after DOX exposure. This finding corroborates the trend observed for decreased MnSOD content when considering the second time-point, and contrasts to what happened in the short-term that revealed a predisposition to increase the CS activity (Figure 8). CS activity was reported to be modulated in heart dysfunction without a significant impact on MnSOD [46], while we saw differences in both these mitochondrial markers. These outcomes emphasize the impact of DOX on cardiac mitochondrial adaptation over time. However, no other differences in the mitochondrial biogenesis markers assessed were observed, suggesting that no impact on mitochondrial biogenesis occurred. The lack of differences in PGC-1α, and their targets Tfam and Mfn1, may be explained by the absence of changes for pAMPK and AMPK content when considering DOX2 and CTRL2, because this kinase is one of the major modulators of PGC-1α [39].

In our study, despite no differences found in AMPK and without assessing the content of the complex of serine/threonine-protein kinases ULK1 and ULK2 (ULK1/2), Beclin1 and ATG5 were significantly decreased in DOX2 compared to CTRL2. This contrasts with the first time-point that showed no differences for these autophagy proteins, indicating different cardiac modulation by DOX over time (Figure 8). Beclin1 recruitment by ULK1/2 is essential to form the phagophore and bring together other autophagy proteins, such as ATG5, which expand the phagophore membrane [47]. Indeed, anthracyclines, including DOX, were associated with the blockage of this initiation step of autophagy [48]. The content of LC3B, which is a direct marker of autophagosome formation, also demonstrated a tendency to be decreased, suggesting that autophagy is decreased at the long-term after DOX exposure, without being impacted in short-term and, consequently, indicating cardiac alterations with time. Curiously, no changes happened for Parkin and BNIP3 content when considering five months after DOX administration, suggesting no influence in mitophagy [27,28]. These mitophagy markers were previously related to interfere in cardiotoxicity caused by anthracyclines, including DOX [48]. Considering the proteins searched herein that act in several steps/pathways of autophagy, it may be inferred this biological process is decreased even five months after DOX administration.

HSP27 content was found to increase in DOX2 compared to CTRL2, but no differences were observed for HSP70. Both HSP27 and HSP70, among other pathways, were described to act in autophagy and apoptosis in opposite directions [26,47,49]. Thus, the increased content of HSP27 that we observed corroborates the decline in autophagy flux and the lack of differences in the apoptotic process when a longer time after DOX exposure has happened [49]. The contribution of HSP70, which seems to act in the autolysosomes formation, is smaller compared to the other autophagy proteins assessed and even to HSP27 [47], thus justifying the non-existence modulation for HSP70 content in our work. However, HSP70 is also involved in several other cellular pathways.

In this work, we also intended to study the influence of DOX on cardiac regeneration, since cardiomyocytes present reduced proliferative ability and would be of great relevance to determine if these markers changed over time. Indeed, after apoptosis, cardiomyocytes are usually replaced by myofibroblasts that promote fibrosis, and therefore there is a loss of cardiac function. The CPCs present specific markers on their surface including the SCFR, which is known to initiate several intracellular cascades that stimulate the proliferation and survival of CPCs [30]. Herein, the content of this receptor was assessed, but no differences were found. Moreover, no differences were found for the content of C/EBPβ and CITED4, which are in accordance with the lack of changes on SCFR (Figure 8), suggesting no influence of DOX on the CPCs pool regardless of the time of exposure in this preclinical model of adult age and at this particular cumulative dose (9 mg/Kg).

## 4. Materials and Methods

### 4.1. Chemicals

Acetyl coenzyme A (acetyl-CoA) sodium salt, bovine serum albumin (BSA), 2,4-dinitrophenylhydrazine (DNPH), 5,5′-dithiobis-(2-nitrobenzoic acid) (DTNB), DOX hydrochloride, horseradish peroxidase-conjugated anti-goat (A5420) antibody, phenylmethanesulfonyl fluoride (PMSF), phosphate-buffered saline (PBS), Ponceau S, protease inhibitors cocktail (P8340), sodium chloride (NaCl), sodium dodecyl sulfate (SDS), trifluoroacetic acid (TFA), Triton X-100, and Tween20 were purchased from Sigma-Aldrich (St. Louis, MO, USA). For information (supplier and reference code) on the primary antibodies used herein see Supplementary Table S2. Horseradish peroxidase-conjugated anti-mouse (NA931) or anti-rabbit (NA934) antibodies were provided by GE Healthcare (Buckinghamshire, UK). Enhanced chemiluminescence (ECL) reagent was purchased from Bio-Rad (Hercules, CA, USA). The chemicals and reagents used were of analytical grade or the highest grade possible.

### 4.2. Animal Experimental Design

Adult (~3 months old) male CD-1 mice (*Mus musculus*) were obtained from Charles River Laboratories (L’Arbresle, France) and maintained in the rodent animal house facility of the Institute of Biomedical Sciences Abel Salazar (ICBAS-UP). Mice were kept according to the conditions and procedures described previously [16]. The protocol was performed following the European Council Directive (2010/63/EU), being the animal protocol approved by the local Committee Responsible for Animal welfare (ORBEA, project nº 140/2015) and by the national competent authorities (General Directorate of Food and Veterinary, DGAV, reference nº 0421/000/000/2016).

In detail, two control groups and two DOX groups were considered (*n =* 9 for each group). DOX solution was prepared in saline solution (0.9% NaCl) and in sterile conditions and was delivered through i.p. injections. A 1.5 mg/kg of DOX injection was given biweekly (on Tuesdays and Thursdays, alternating the place of administration on the animal’s abdomen) for three weeks, so the DOX animals received in the end a total cumulative dose of 9 mg/kg. The control animals received six i.p. injections of 0.9% NaCl/Kg in the same days and conditions as the DOX group. The administration schedule was planned to mimic the multiple administrations at separated time-points given in human therapy [6]. The human equivalent dosage was calculated based on allometric scaling and considering the conversion factor 37 for body area surface between mice and humans, as recommended by the Food and Drug Administration [50]. The 9 mg/Kg cumulative dose of DOX corresponds roughly to ~50 mg/m^2^ in humans, which is much lower than the maximum lifelong dose recommended for humans (400–550 mg/m^2^) [12].

The control and DOX-treated animal groups sacrificed one week (first time-point) after the last i.p. injection were named CTRL1 and DOX1, respectively. The animal groups sacrificed five months (second time-point) after the last injection were named CTRL2 (control animals) and DOX2 (DOX-treated). Animals started with approximately 12 weeks old (about 20–21 human years) and at the end of the administrations they were 15 weeks old (about 23–24 human years). In the first time-point of sacrifice, mice were approximately 16 weeks old, which corresponds nearly to 24 years old in human years. At the second time-point, five months had passed, and animals were 36 weeks old, which roughly corresponds to 35–36 human years [33]. Therefore, this protocol allows an extensive follow-up of cardiac damage in laboratory animals, which is extremely rare in preclinical studies in the cardio-oncology field.

### 4.3. Blood Collection and Serum Evaluation

At sacrifice, the animals were anesthetized with isoflurane inhalation and sacrificed by exsanguination. In detail, the abdominal cavity was opened for blood collection through the inferior vena cava for commercial tubes containing an inert clotting agent (Vetlab ZT Plain + Gel, 1.1 mL fill, Vetlab Supplies, West Sussex, UK). Then, blood was left at room temperature for at least 45 min to form the clot that was further pelleted by centrifugation (900× *g*, 10 min, room temperature). Taken serum was aliquoted and stored at −80 °C until the biochemical analysis. An aliquot was analyzed for total protein, albumin, and glucose concentration, as well as CK-MB activity, using an AutoAnalyzer (Prestige 24i, Cormay PZ, Diamond Diagnostics, Holliston, MA, USA). The content of serum CRP and nitrotyrosine was assessed by immunoblotting (see *Immunoblotting Evaluation* section). For the determination of serum protein carbonyl levels, slot-blot was used after derivatization of proteins. In this case, a given volume (V) of serum was mixed with 1 V of 12% SDS, 2 V of 2 mM DNPH/10% TFA, and incubated in dark for 30 min. Afterwards, 1.5 V of 2 M Tris-base/18% of β-mercaptoethanol was added to stop the reaction. The derivatized proteins were diluted 1:150 in tris-buffered saline (TBS) before the immunoblotting.

### 4.4. Heart Collection, Histological Analysis, and Homogenization

Following blood collection, the hearts were excised and weighed. The heart apex was used for the histological analysis that was carried out as previously described [19,51]. After hematoxylin and eosin staining, the images were taken with a Carl Zeiss Imager A1 light microscope equipped with an AxioCam MRc 5 digital camera (Oberkochen, Germany) to assess tissue damage. A portion containing the two ventricles was homogenized using lysis buffer (100 mM potassium phosphate, pH 7.4, 0.1% [*v*/*v*] Triton X-100, with protease inhibitors cocktail [1:400] and PMSF [1:1000]; 50 mg of tissue/mL of buffer) using a Teflon pestle on a tight-fitting Potter–Elvehjem glass homogenizer at 0–4 °C. Aliquots of cardiac homogenates were stored at −80 °C for further analysis. Protein content was estimated with the commercial method DC Protein Assay (Bio-Rad), using BSA as standard and the manufacturer’s instructions.

### 4.5. Immunoblotting Evaluation

Serum samples were diluted in TBS (for CRP and nitrotyrosine evaluation samples were diluted 1:50, while for 2,4-dinitrophenyl hydrazone moiety detection a 1:150 dilution was used) and afterwards slot-blotted (50 µL) onto a nitrocellulose membrane (Amersham Protran, GE Healthcare, Munich, Germany) under a vacuum system.

In the heart homogenates, nitrotyrosine content (10 μg) and protein carbonyl levels (20 μg) were assessed through slot-blot. The other cardiac proteins were evaluated by Western blot (30 μg) using a 12.5% SDS-PAGE and following Laemmli [52]. The resolved proteins were blotted for 1 h at 200 mA in transfer buffer.

The blockage of membranes was performed with 5% (*w*/*v*) nonfat dry milk in TBS with Tween 20 (TBS-T) for 1 h. Then, the membranes were incubated with the primary antibody for 90 min at room temperature (serum proteins) or overnight at 4 °C (cardiac proteins). For further information regarding the primary antibodies used, and their application and dilution, see Supplementary Table S2. After the incubation with primary antibodies, membranes were washed with TBS-T and incubated with the appropriate secondary horseradish peroxidase-conjugated antibody, diluted 1:1000 or 1:2000 in 5% (*w*/*v*) nonfat dry milk prepared in TBS-T. After washing with TBS-T, membranes were incubated with ECL reagent following manufacturer’s recommendations. The immunoreactive bands were automatically detected using the ChemiDoc XRS+ Imaging System (Bio-Rad) and the images obtained were analyzed with Image Lab software version 6.0.1 (Bio-Rad). The efficacy of protein transfer/loading was controlled by the Ponceau S staining instead of housekeeping markers since most of them are affected in the cardiac tissue by DOX [38,41]. Ponceau S staining of all membranes, as well as the blots obtained, are presented in Supplementary Figure S1. Results are presented in arbitrary units of optical density.

### 4.6. Citrate Synthase (CS) Activity Evaluation

The activity of CS was determined by measuring the 2-nitro-5-thiolbenzoate anion at 412 nm (molar extinction coefficient of 13.6 mM^−1^ cm^−1^), following Coore et al. [53]. This anion results from the reaction of DTNB with the free thiol groups of CoA, which are formed during the reaction of acetyl-CoA with oxaloacetate. The absorbance was read for approximately 2 min at 30 °C using a microplate reader (Multiskan GO, Thermo Fisher Scientific, Northumberland, UK) and the values were normalized to the amount of protein in the heart homogenates.

### 4.7. Statistical Analysis

Results are indicated as mean ± standard deviation (SD). Differences among the experimental groups were analyzed by the unpaired two-sided *t*-test of GraphPad Prism software (version 6.0.1). Values obtained were considered statistically significant when the *p*-value < 0.05. For *p*-values < 0.1, a tendency was assumed.

## 5. Conclusions

Preclinical evaluation of the long-term cardiotoxicity of anticancer drugs is lacking. Thus, as far as we are aware, this is one of the few studies to evaluate the cardiac effects of a clinically relevant dose (9 mg/Kg) of DOX at short-term (one week) and for such a long time after exposure (five months). Indeed, the cardiac AOPs observed differ between the two time-points, highlighting the distinct manifestations for early and late chronic cardiotoxicity, and implying cardiac adaptation after DOX exposure with time. Cardiac energy metabolism was one of the pathways that showed a clear adaptation over time. It was greatly impacted in the short-term, while the long-term did not promote major alterations at the protein level, showing the flexibility of the heart after insult. Indeed, decreased content of cardiac AMPK, the main metabolic regulator of the oxidation of glucose and fatty acids, and increased content of ETF-QO suggest increased FAO in the short-term after DOX treatment. These metabolic changes were accompanied by increased apoptosis given by the increased BAX content in the short-term, suggesting higher elimination of damaged cardiac cells. At the same time, it seems likely that mitochondria became more numerous after treatment, according to the decreased content of Mfn1 and the tendency to increase CS activity. Nevertheless, the DOX fingerprint was still present five months after administration, and this cardiotoxic agent was able to decrease the activity of CS, pointing to decreased mitochondrial density, and being in accordance with the predisposition seen for the content of the mitochondrial enzyme MnSOD. Moreover, a general decrease in the content of autophagy markers, including Beclin1 and ATG5, and less evident, but still relevant, LC3B, was observed, showing disruption of autophagy, a pathway that was not impacted at the short-term. In addition, HSP27 content was found to have increased, suggesting the higher modulation of autophagy in the long-term after DOX exposure. Thus, the cardiac adaptation seen over time, extrapolated through the different outcomes observed for the two time-points selected after the DOX exposure, is indicative of the huge importance to place a warning regarding the long-term evaluation of cardiac health on patients treated with anthracyclines. Moreover, finding an early biomarker of damage before irreversible clinical damage is present and the recommendation for maximum lifetime cumulative doses for each patient, are of outmost importance.

## Figures and Tables

**Table 1:** Morphometric parameters measured at the sacrifice day (one week for CTRL1 and DOX1, and five months for CTRL2 and DOX2, after the last i.p. injection). Values are expressed as mean ± standard deviation (SD, *n* = 9 for CTRL1, DOX1, and CTRL2; *n =* 7 for DOX2). Statistical analyses were carried out using unpaired two-sided *t*-test: * *p* < 0.05, ** *p* < 0.01 vs. CTRL2.

Morphometric Parameter	CTRL1	DOX1	CTRL2	DOX2
Whole-body weight (g)	42.671 ± 3.450	40.557 ± 2.348	47.525 ± 2.416	43.167 ± 4.373 *
Heart weight (g)	0.228 ± 0.048	0.216 ± 0.028	0.246 ± 0.017	0.220 ± 0.025 *
Tibial length (cm)	1.90 ± 0.08	1.90 ± 0.13	2.02 ± 0.05	1.92 ± 0.06 **
Heart weight to whole-body weight (mg/g)	5.30 ± 0.81	5.33 ± 0.61	5.20 ± 0.55	5.11 ± 0.42
Heart weight to tibial length (g/cm)	0.120 ± 0.025	0.114 ± 0.015	0.122 ± 0.007	0.115 ± 0.014

## Data Availability

The data presented in this study are available on request from the corresponding author.

## Supplementary Materials

The following supporting information can be downloaded at: https://www.mdpi.com/article/10.3390/ph16111613/s1, Supplementary Figure S1: Ponceau S staining and Slot/Western blots images for each protein evaluated; Supplementary Table S1: Biomarkers found modulated after DOX administration for the two time-points selected. The assessment (morphometric/serum/cardiac tissue) and the biological/molecular pathway of each biomarker is indicated. No changing (=), upregulation (↑) and downregulation (↓) is indicated for each time-point considering the modulation found for DOX compared to CTRL group. The predisposition to increased or decreased values in DOX compared to the CTRL group is also indicated; Supplementary Table S2: Specification of the supplier, reference code, host species, purity, and dilutions for each primary antibody used as well as their application on serum (S) or cardiac (C) samples. Antibodies were diluted 1:200 to 1:1000 in 5% (*w*/*v*) nonfat dry milk prepared in TBS-T.

## Abbreviations

AMPK: AMP-activated protein kinase, AOPs: adverse outcome pathways, ATG5: autophagy protein 5, ATPB: ATP synthase subunit β, BAX: BCL2 associated X-protein, BCL2: B-cell lymphoma-2, BNIP3: BCL2 interacting protein 3, C/EBPβ: CCAAT/enhancer-binding protein β, CITED4: Cbp/p300-interacting transactivator 4, CK-MB: creatine kinase-MB, CoA: coenzyme A, CPCs: cardiac progenitor cells, CRP: C reactive protein, CS: citrate synthase, CTRL1: control group at first time-point, CTRL2: control group at second time-point, DOX1: doxorubicin group at first time-point, DOX2: doxorubicin group at second time-point, ETF-QO: electron transfer flavoprotein-ubiquinone oxidoreductase, FAO: fatty acids oxidation, GLUT4: glucose transporter GLUT4, GSK-3β: glycogen synthase kinase 3 β, HSP: heat shock protein, i.p.: intraperitoneal, LC3B: microtubule-associated protein light chain 3, Mfn1: mitofusin1, MnSOD: manganese superoxide dismutase, NaCl: sodium chloride, OXPHOS: oxidative phosphorylation, pAMPK: phosphorylated form of AMP-activated protein kinase, PFKM: phosphofructokinase, PGC-1α: peroxisome proliferator-activated receptor γ coactivator 1 α, PPAR: peroxisome proliferator-activated receptor, ROS: reactive oxygen species, RNS: reactive nitrogen species, SCFR: mast/stem cell growth factor receptor Kit, SD: standard deviation, SDS: sodium dodecyl sulfate, SIRT3: sirtuin 3, TBS: Tris buffered saline, TBS-T: Tris buffered saline with Tween 20, Tfam: mitochondrial transcription factor A, ULK1/2: complex of serine/threonine-protein kinases ULK1 and ULK2.
