# Supplementary material for: The Metabolic Fingerprint of Doxorubicin-Induced Cardiotoxicity in Male CD-1 Mice Fades Away with Time While Autophagy Increases
Source: Pharmaceuticals (Basel). 2023 Nov 15;16(11):1613. doi: 10.3390/ph16111613 (PMC10675798; doi:10.3390/ph16111613)

Supplementary Figure S1. Ponceau S staining and Slot/Western blots images for each protein evaluated.

CRP - serum

PonceauS

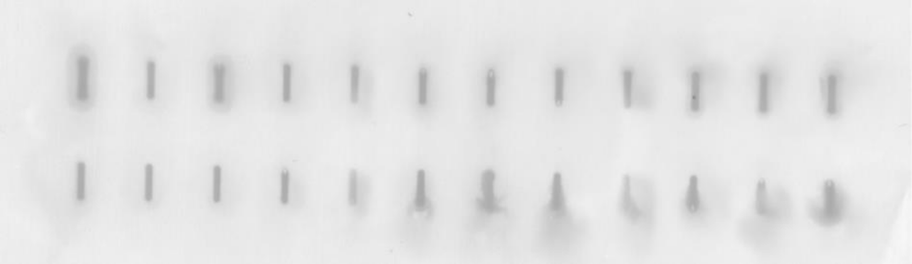

Blot

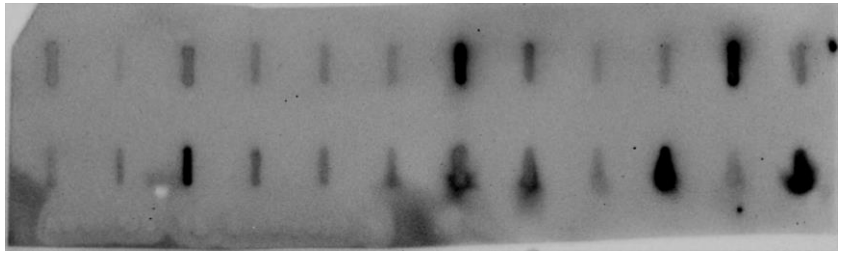

Samples order from upper left corner: CTRL1-1 | DOX1-1 | CTRL2-1 | DOX2-1 | CTRL1-2 | DOX1-2 | CTRL2-2 | DOX2-2 | CTRL1-3 | DOX1-3 | CTRL2-3 | DOX2-3
CTRL1-4 | DOX1-4 | CTRL2-4 | DOX2-4 | CTRL1-5 | DOX1-5 | CTRL2-5 | DOX2-5 | CTRL1-6 | DOX1-6 | CTRL2-6 | DOX2-6

DNP (protein carbonyl levels) - serum

Blot

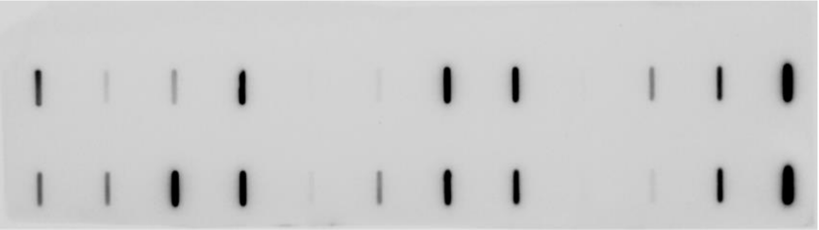

Samples order from upper left corner: CTRL1-1 | DOX1-1 | CTRL2-1 | DOX2-1 | CTRL1-2 | DOX1-2 | CTRL2-2 | DOX2-2 | CTRL1-3 | DOX1-3 | CTRL2-3 | DOX2-3
CTRL1-4 | DOX1-4 | CTRL2-4 | DOX2-4 | CTRL1-5 | DOX1-5 | CTRL2-5 | DOX2-5 | CTRL1-6 | DOX1-6 | CTRL2-6 | DOX2-6

Nitrotyrosine - serum

PonceauS

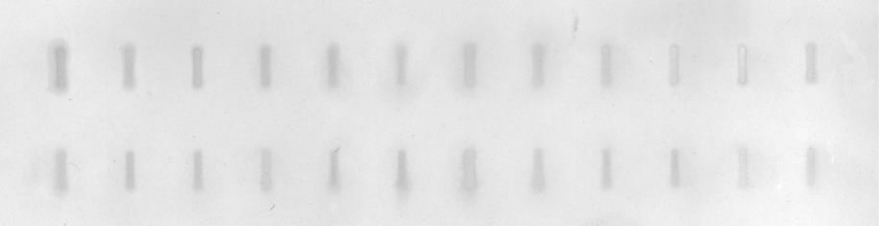

Blot

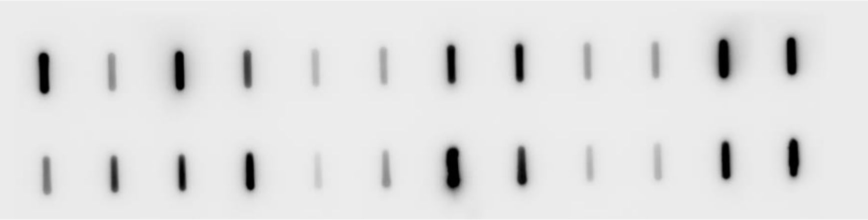

Samples order from upper left corner: CTRL1-1 | DOX1-1 | CTRL2-1 | DOX2-1 | CTRL1-2 | DOX1-2 | CTRL2-2 | DOX2-2 | CTRL1-3 | DOX1-3 | CTRL2-3 | DOX2-3
CTRL1-4 | DOX1-4 | CTRL2-4 | DOX2-4 | CTRL1-5 | DOX1-5 | CTRL2-5 | DOX2-5 | CTRL1-6 | DOX1-6 | CTRL2-6 | DOX2-6

GLUT4 - PonceauS

1

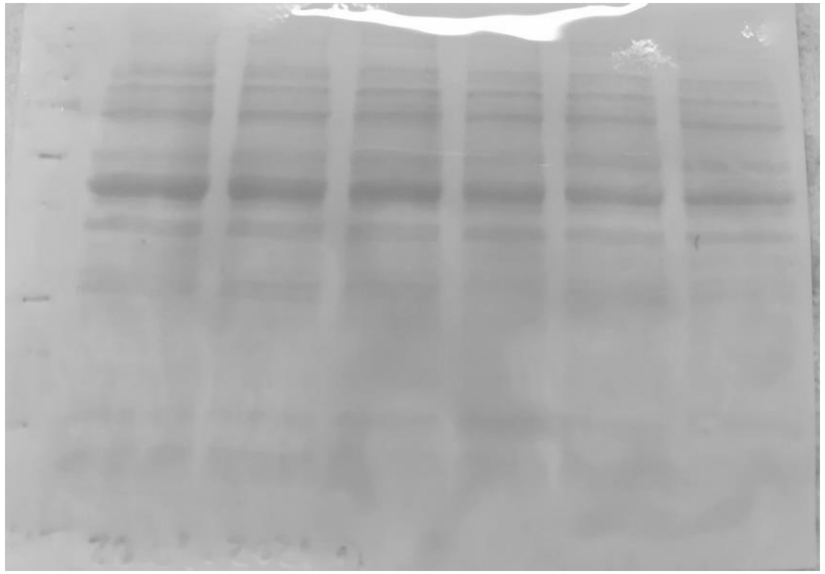

2

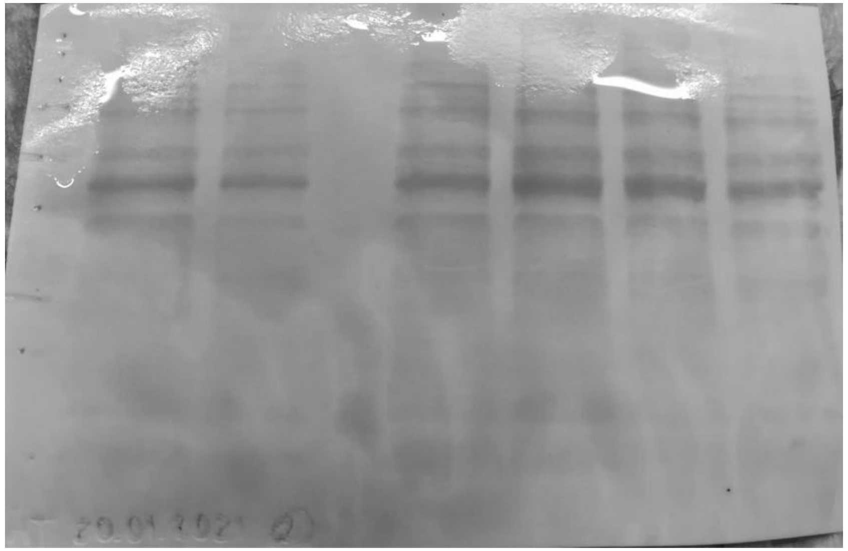

3

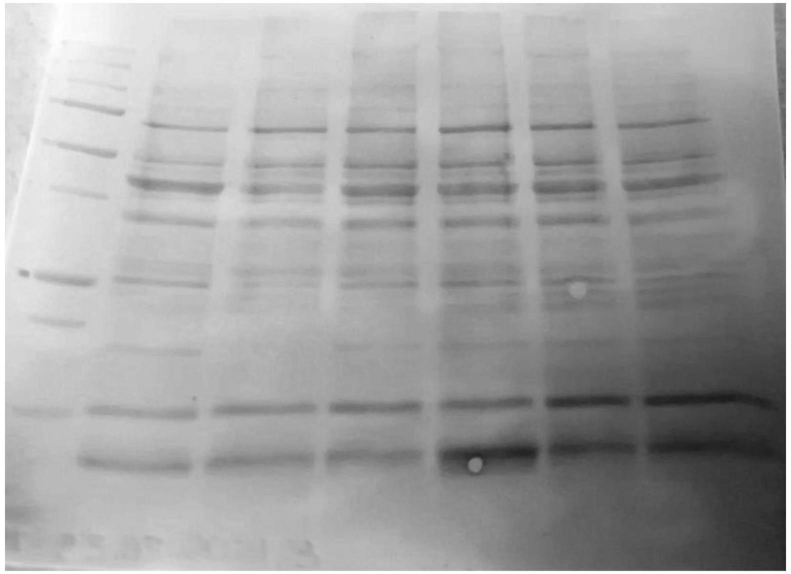

4

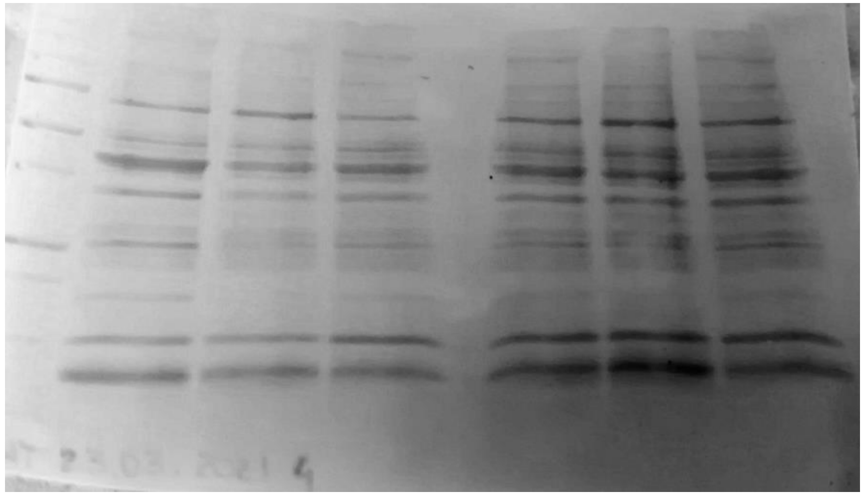

5

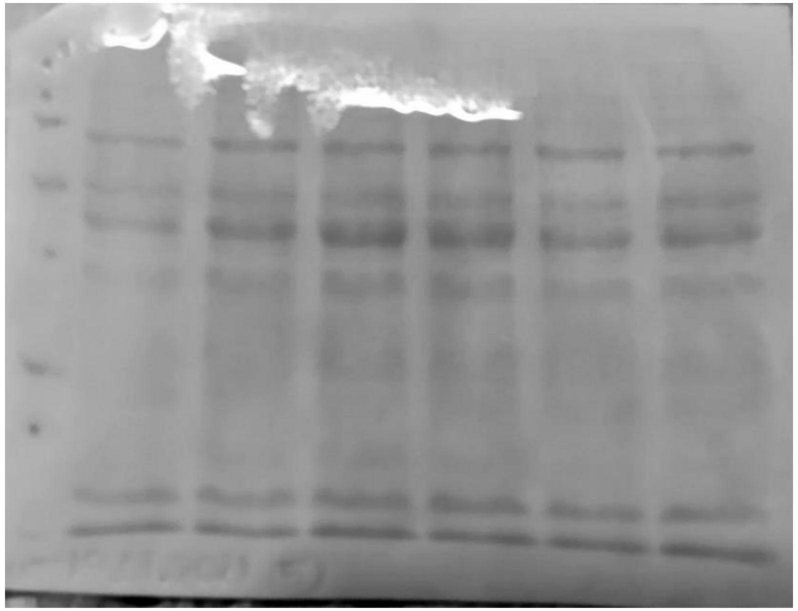

6

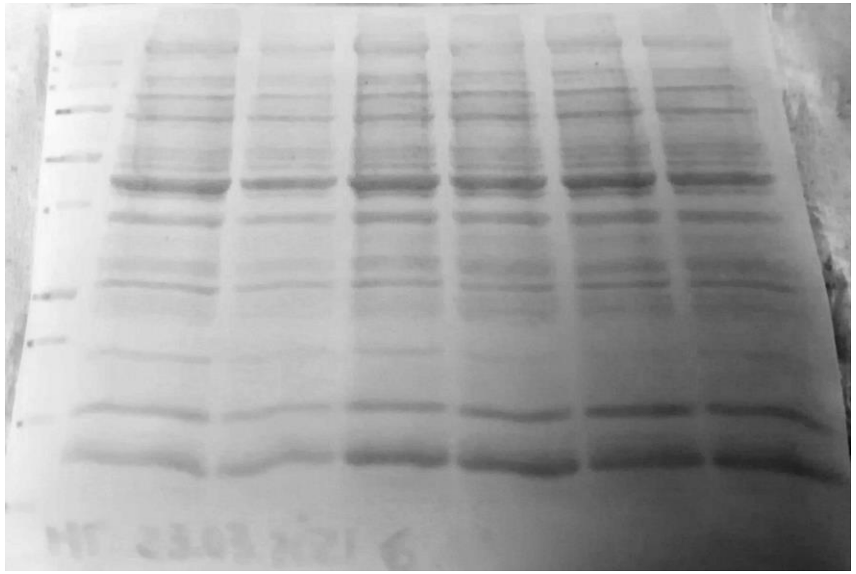

MWM: molecular weight marker

Samples order from the left in each of the 6 membranes: MWM | MTX | DOX18 | **CTRL1 | DOX1 | CTRL2 | DOX2**

GLUT4 - Blot

1

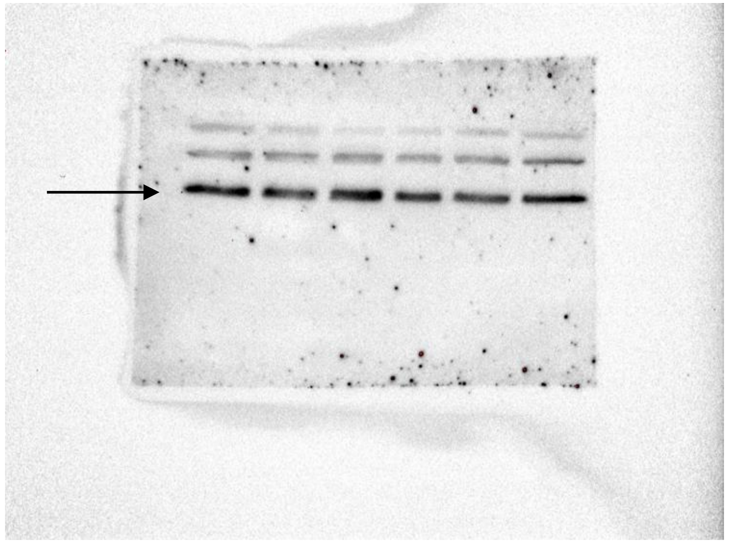

2

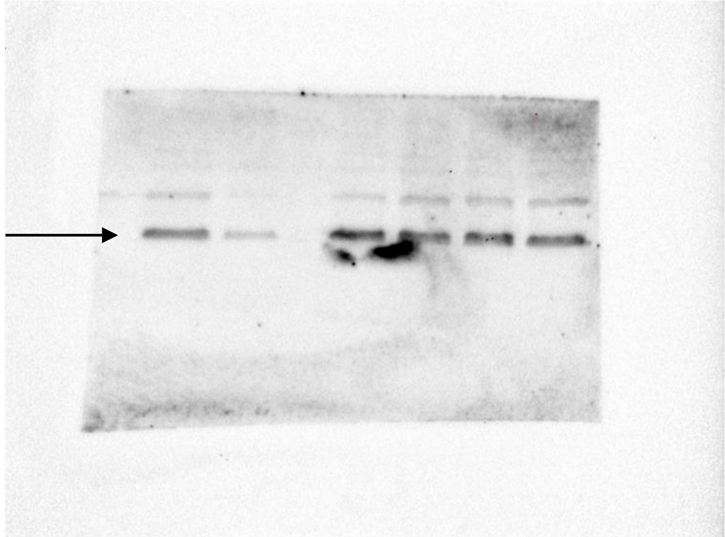

3

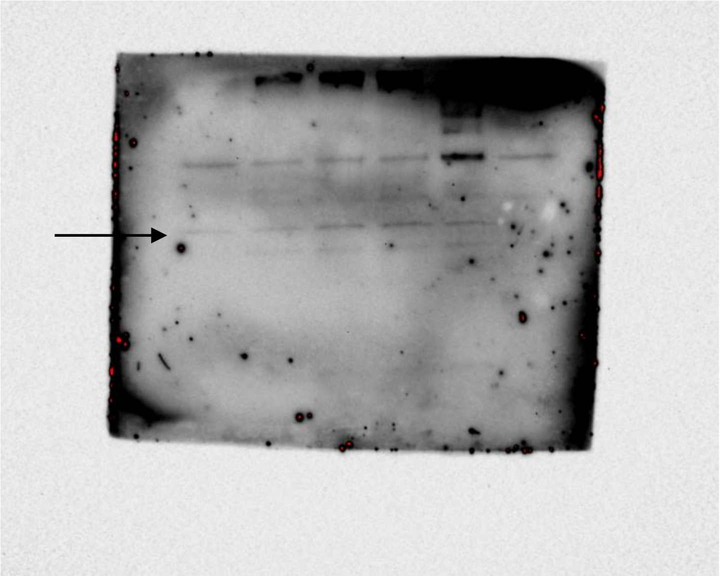

4

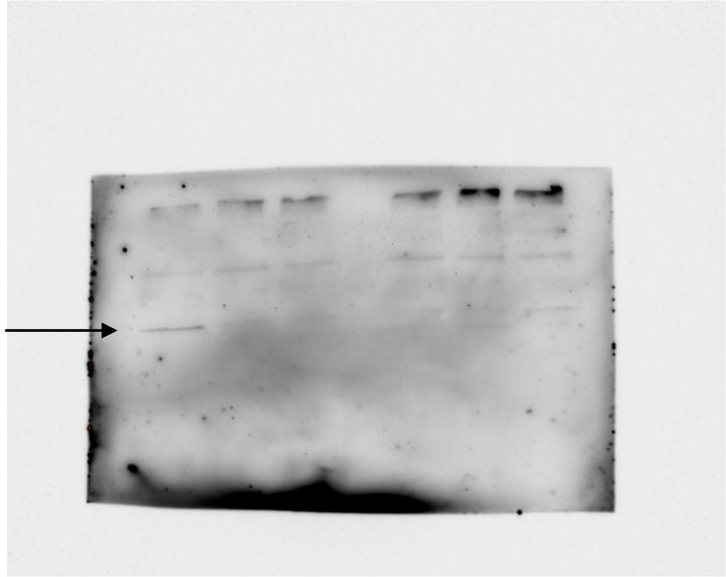

5

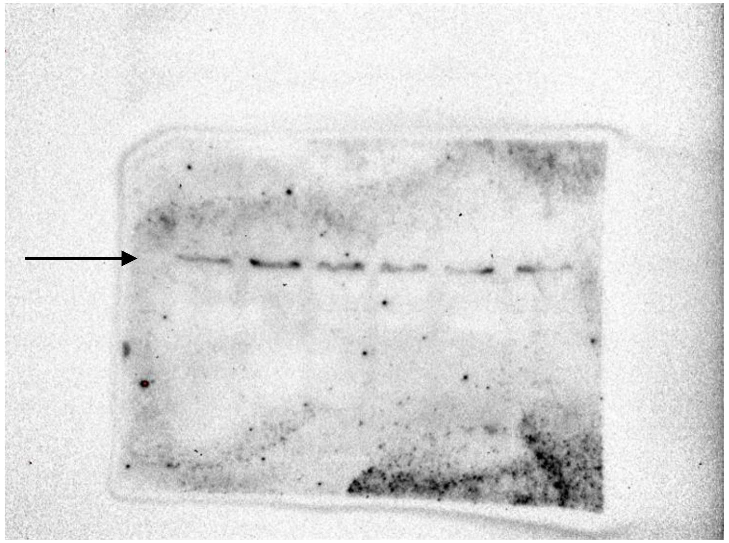

6

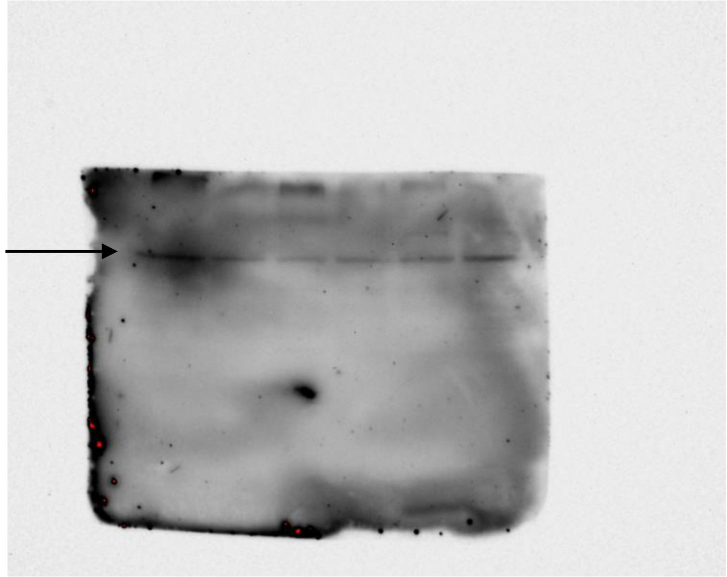

MWM: molecular weight marker

Samples order from the left in each of the 6 membranes: MWM | MTX | DOX18 | **CTRL1 | DOX1 | CTRL2 | DOX2**

PFKM - PonceauS

1

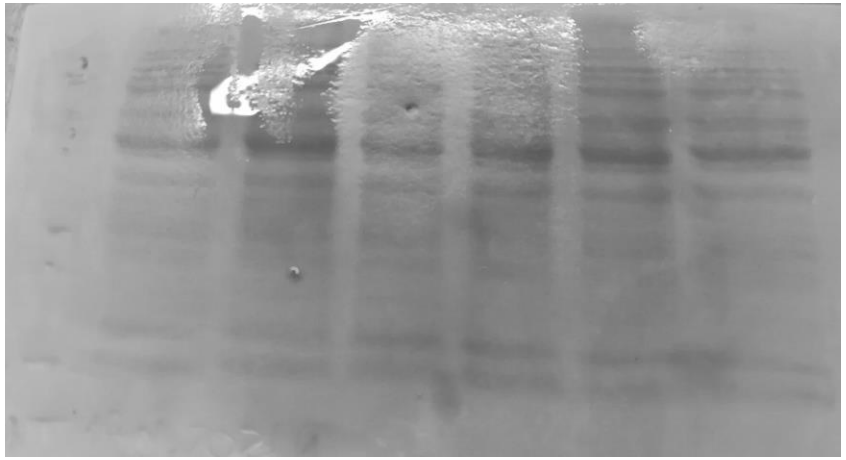

2

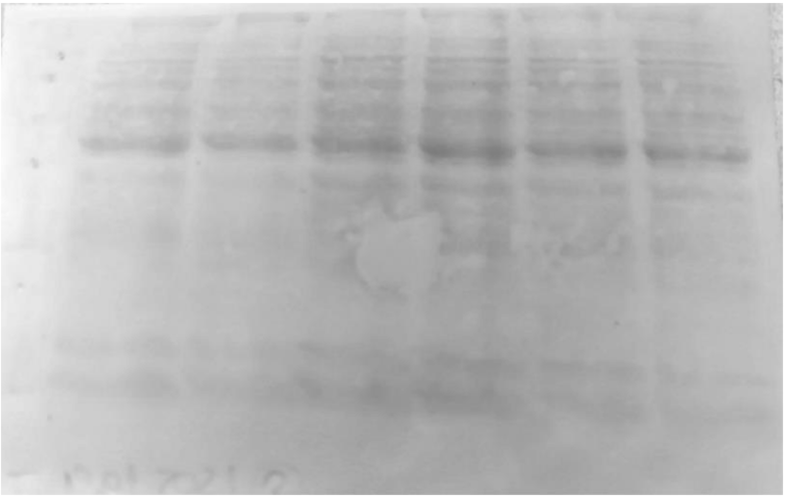

3

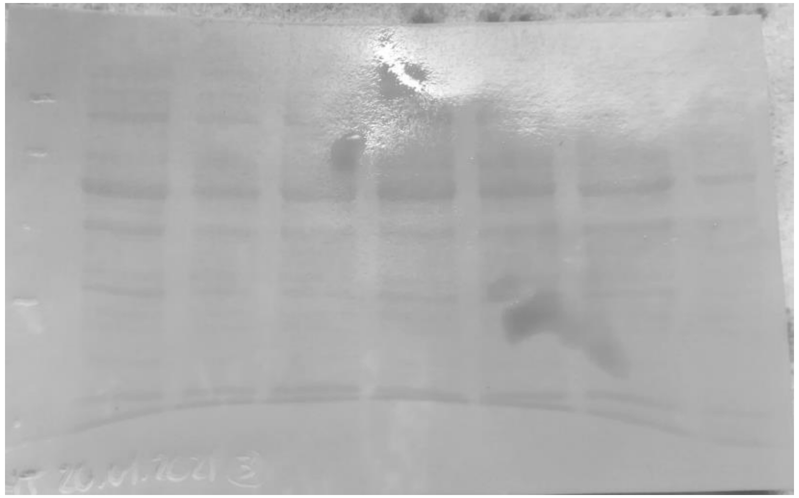

4

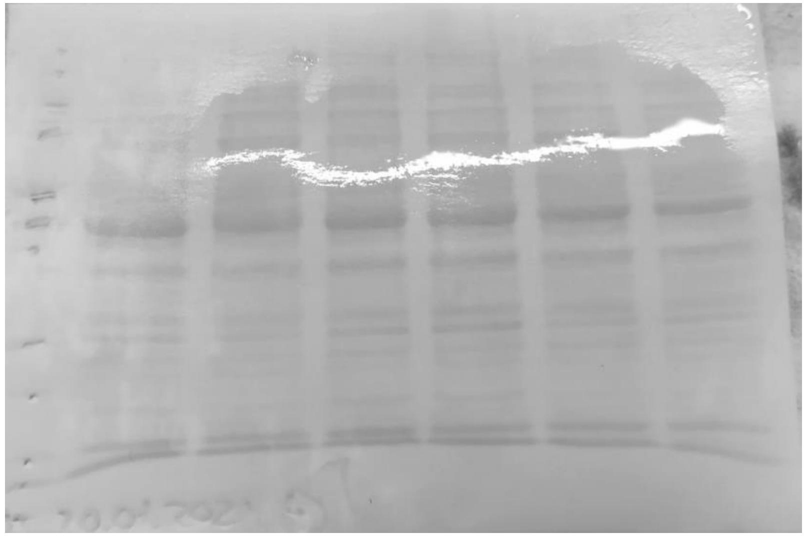

5

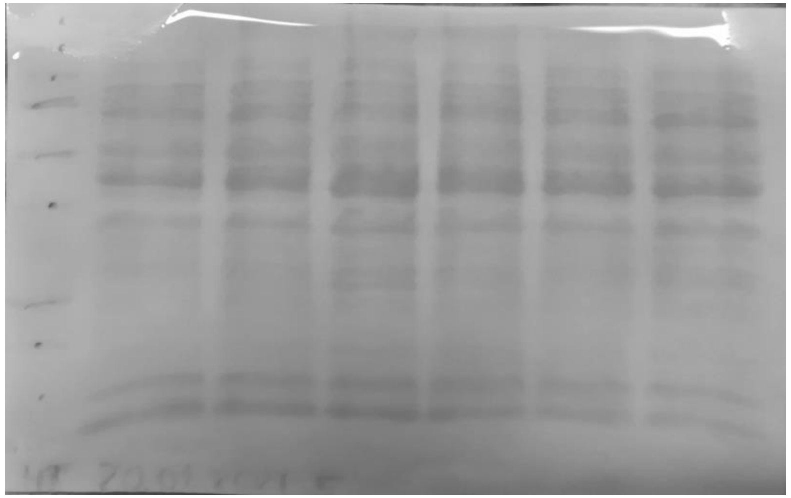

6

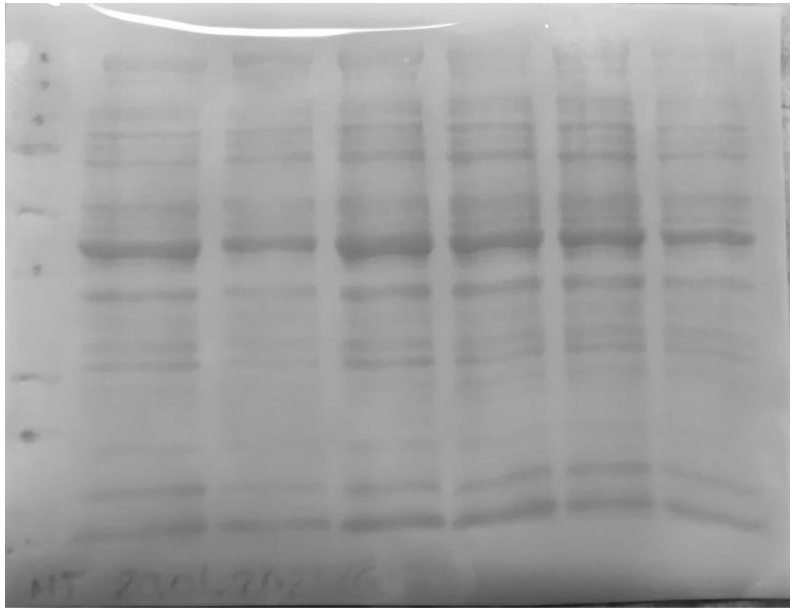

MWM: molecular weight marker

Samples order from the left in each of the 6 membranes: MWM | MTX | DOX18 | **CTRL1 | DOX1 | CTRL2 | DOX2**

PFKM - Blot

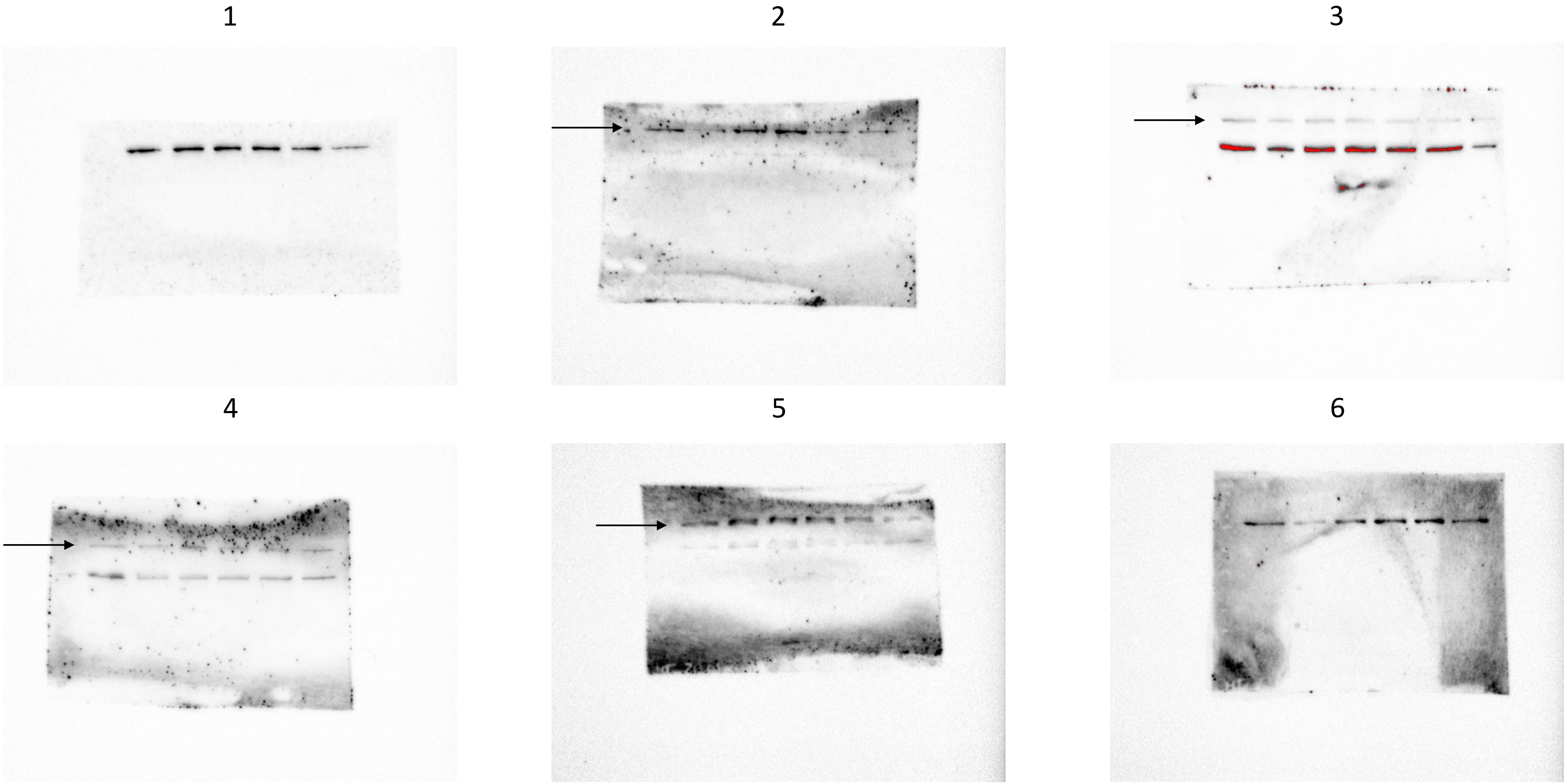

MWM: molecular weight marker

Samples order from the left in each of the 6 membranes: MWM | MTX | DOX18 | **CTRL1 | DOX1 | CTRL2 | DOX2**

ETF-QO - PonceauS

1

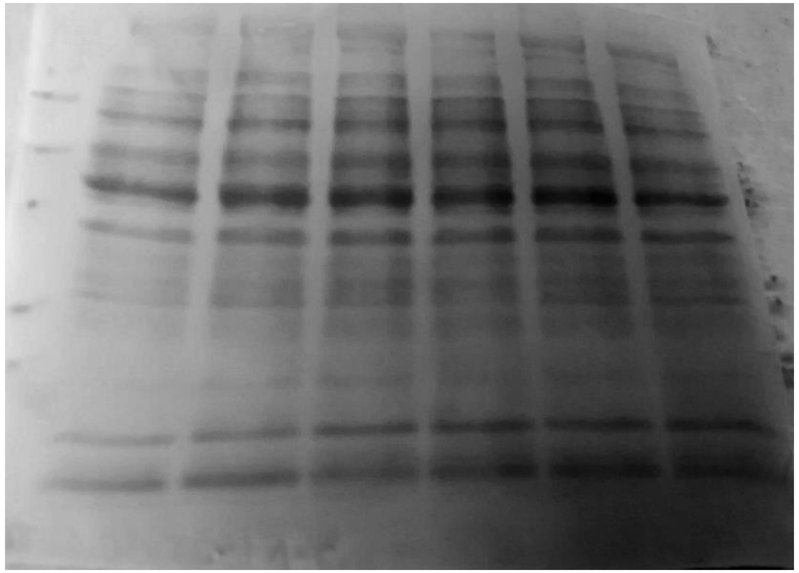

2

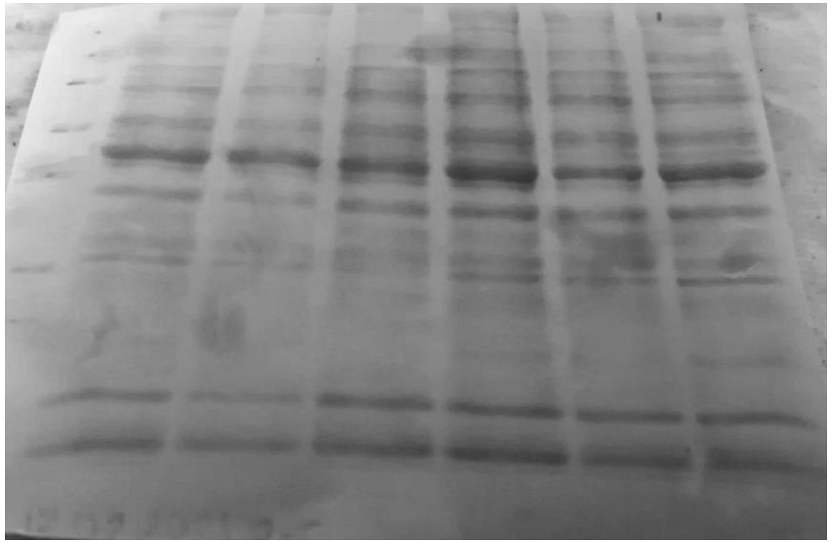

3

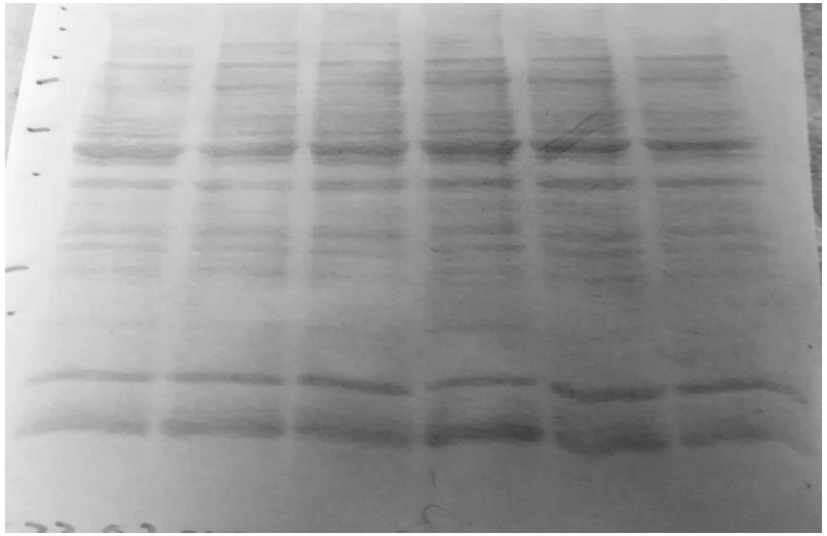

4

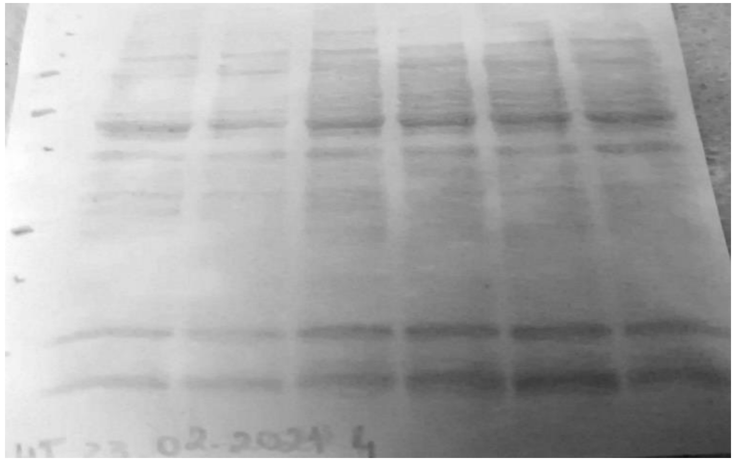

5

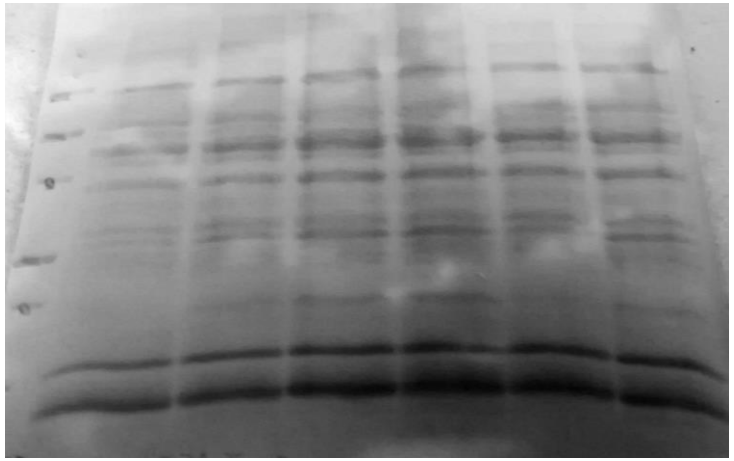

6

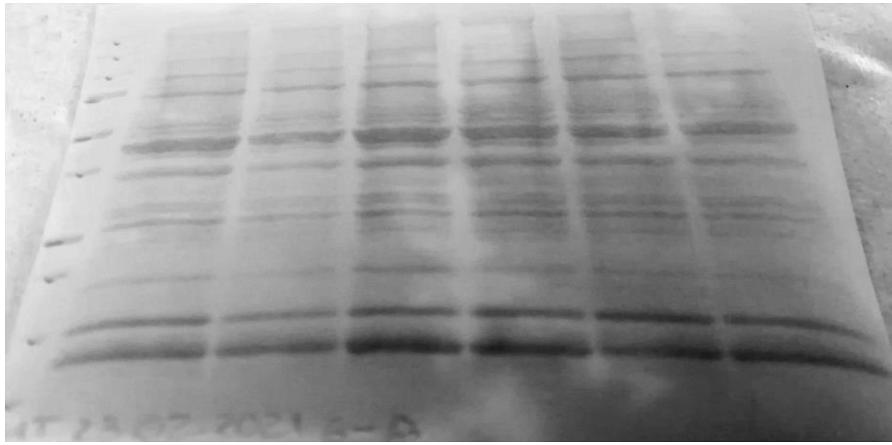

MWM: molecular weight marker

Samples order from the left in each of the 6 membranes: MWM | MTX | DOX18 | **CTRL1 | DOX1 | CTRL2 | DOX2**

ETF-QO - Blot

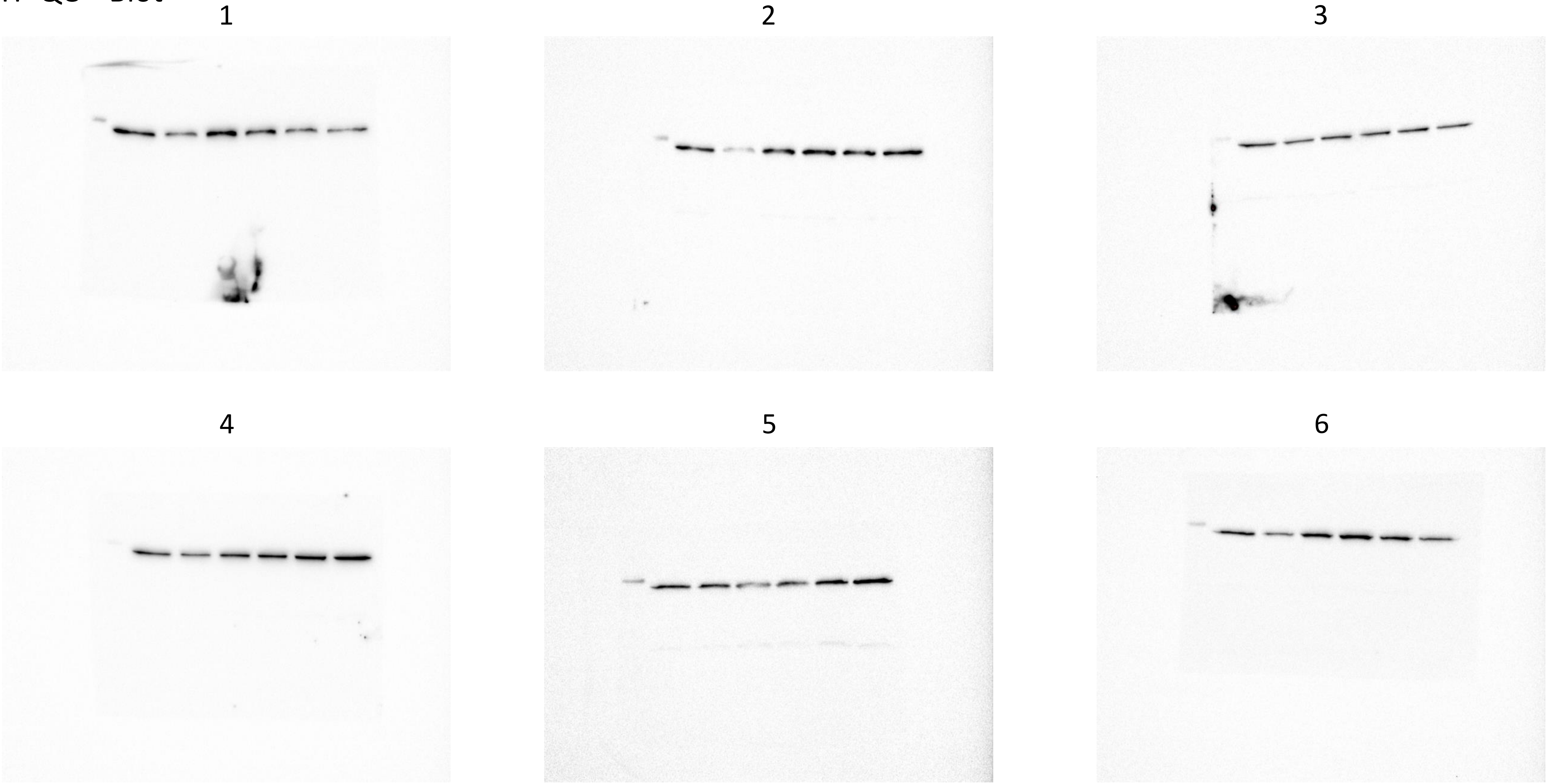

MWM: molecular weight marker

Samples order from the left in each of the 6 membranes: MWM | MTX | DOX18 | **CTRL1 | DOX1 | CTRL2 | DOX2**

ATPB - PonceauS

1

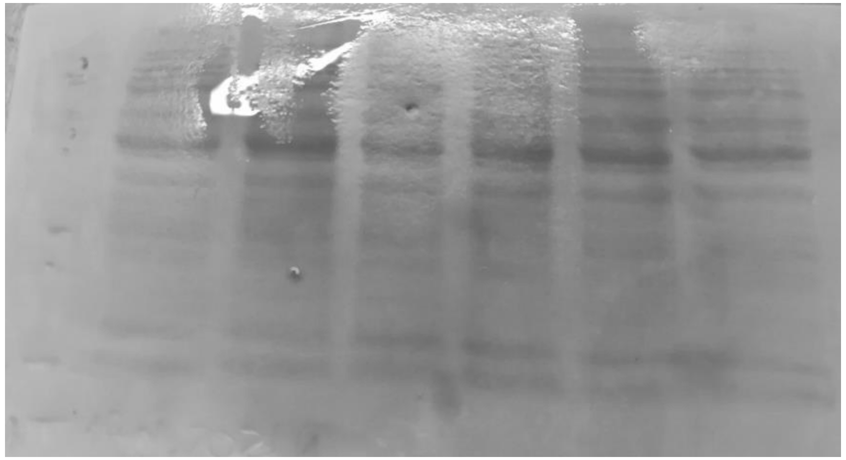

2

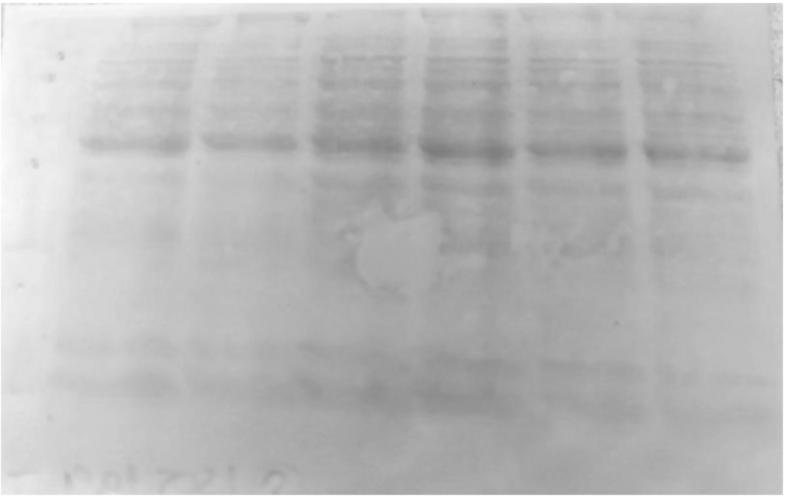

3

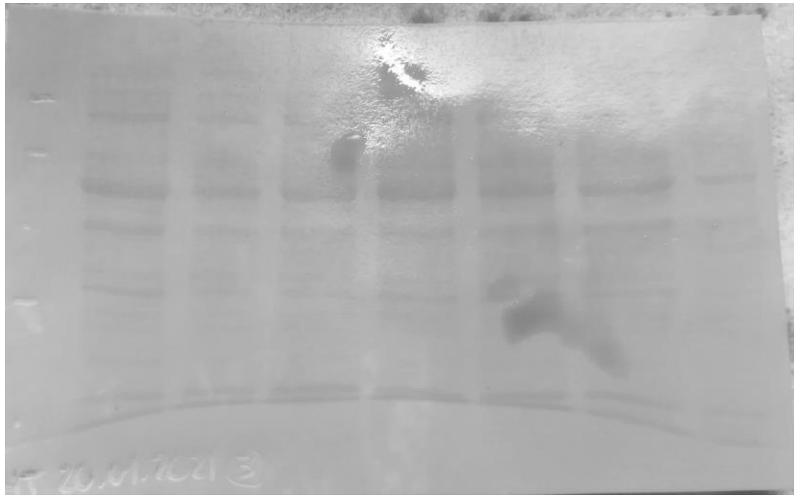

4

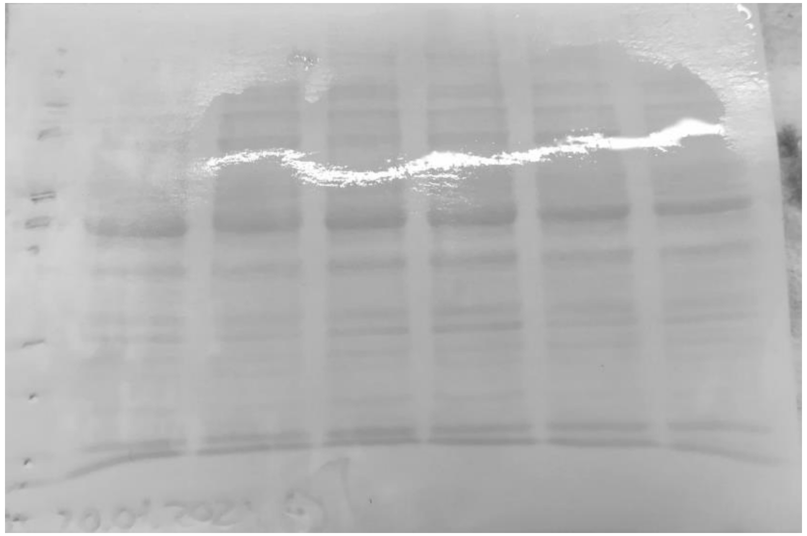

5

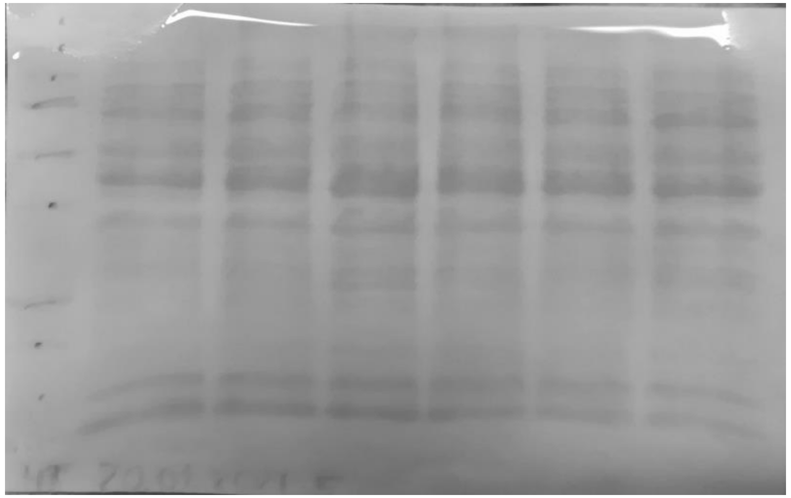

6

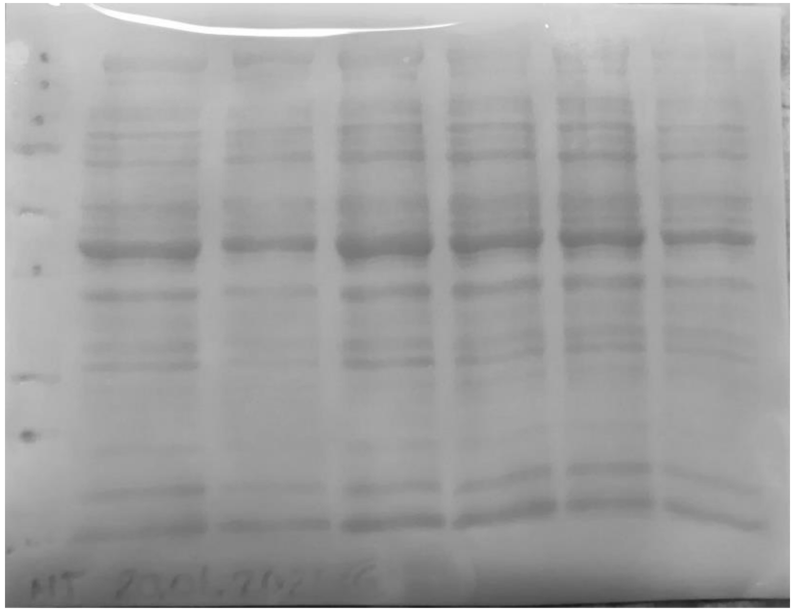

MWM: molecular weight marker

Samples order from the left in each of the 6 membranes: MWM | MTX | DOX18 | **CTRL1 | DOX1 | CTRL2 | DOX2**

ATPB - Blot

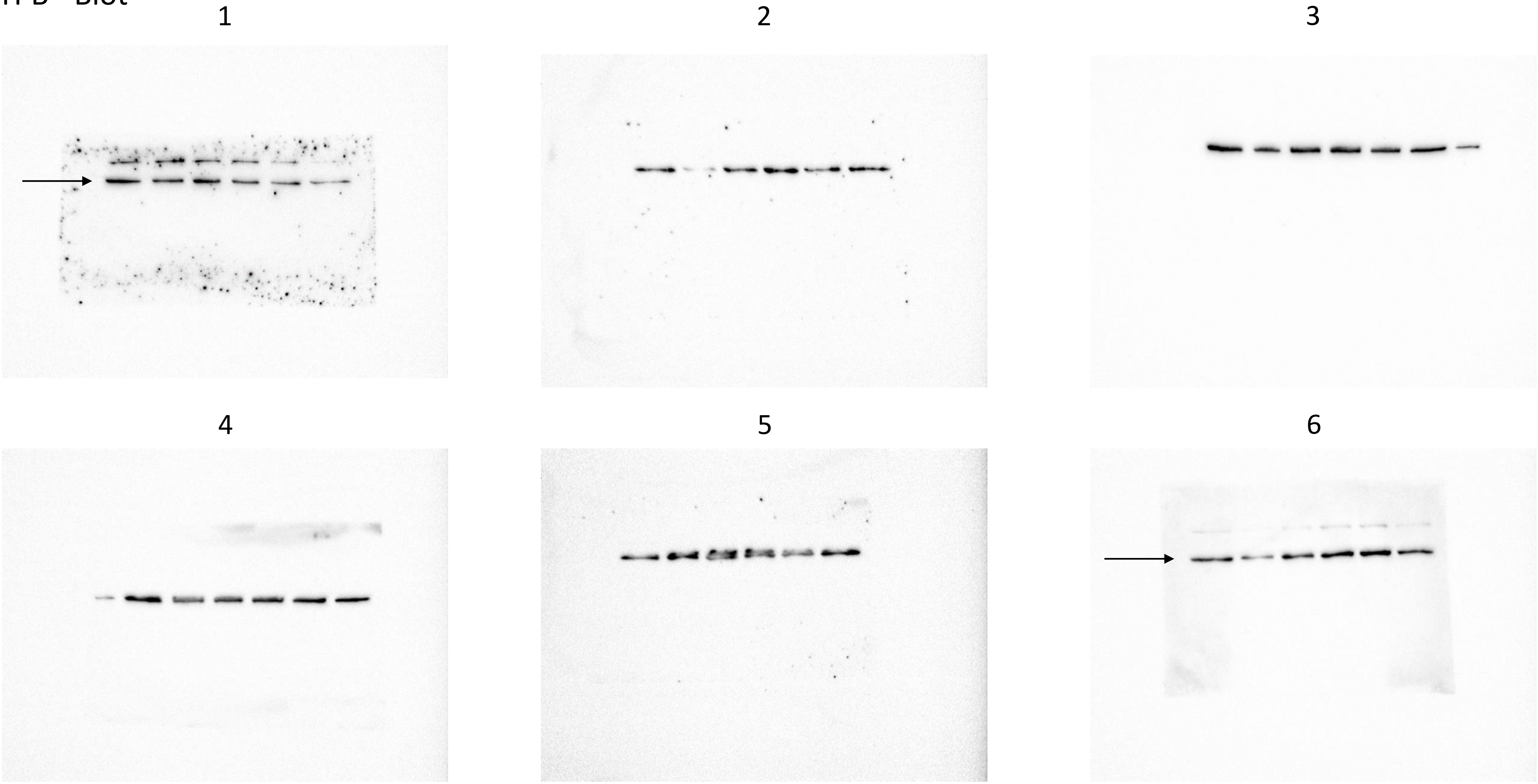

MWM: molecular weight marker

Samples order from the left in each of the 6 membranes: MWM | MTX | DOX18 | **CTRL1 | DOX1 | CTRL2 | DOX2**

SIRT3 - PonceauS

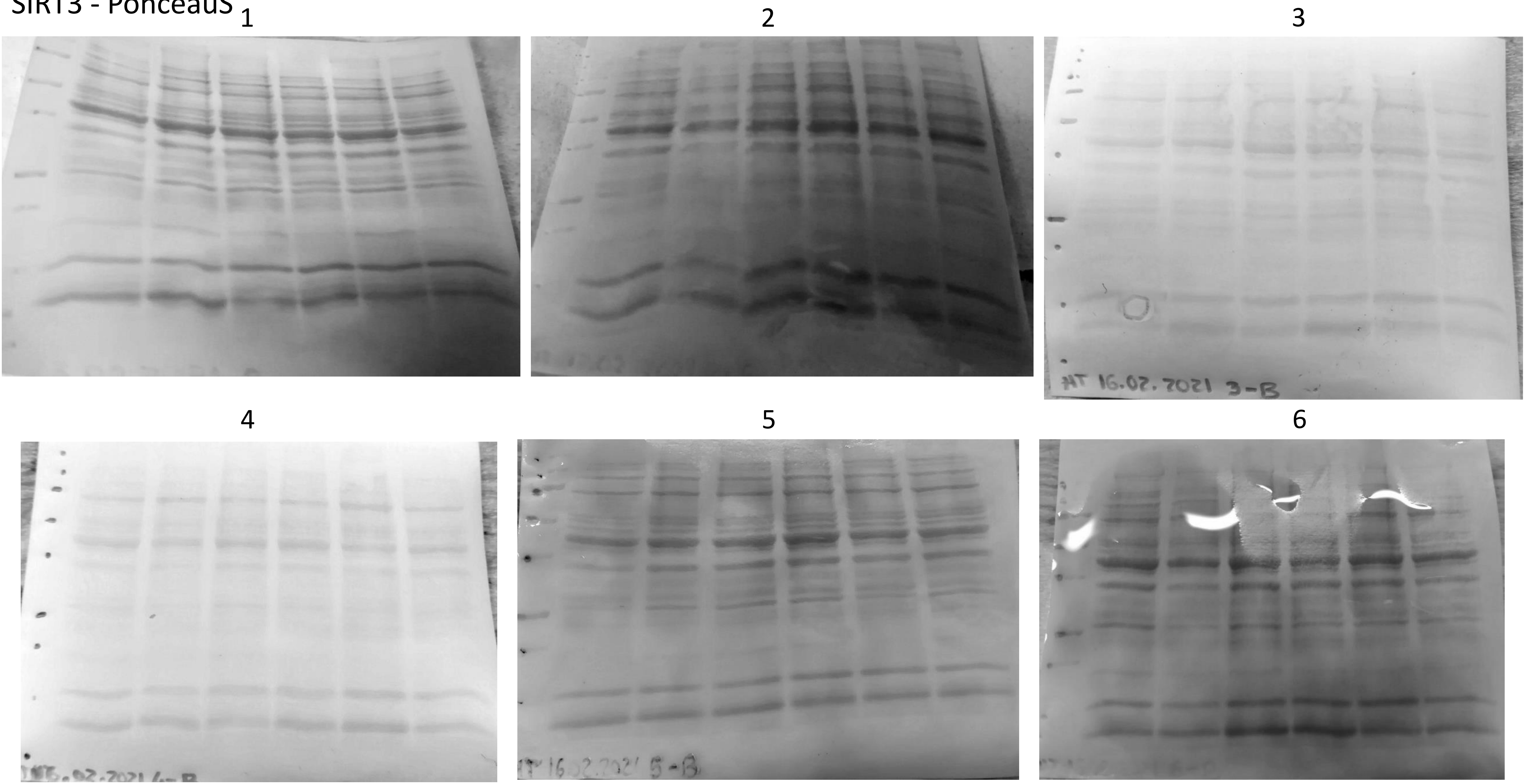

MWM: molecular weight marker

Samples order from the left in each of the 6 membranes: MWM | MTX | DOX18 | **CTRL1 | DOX1 | CTRL2 | DOX2**

SIRT3 - Blot

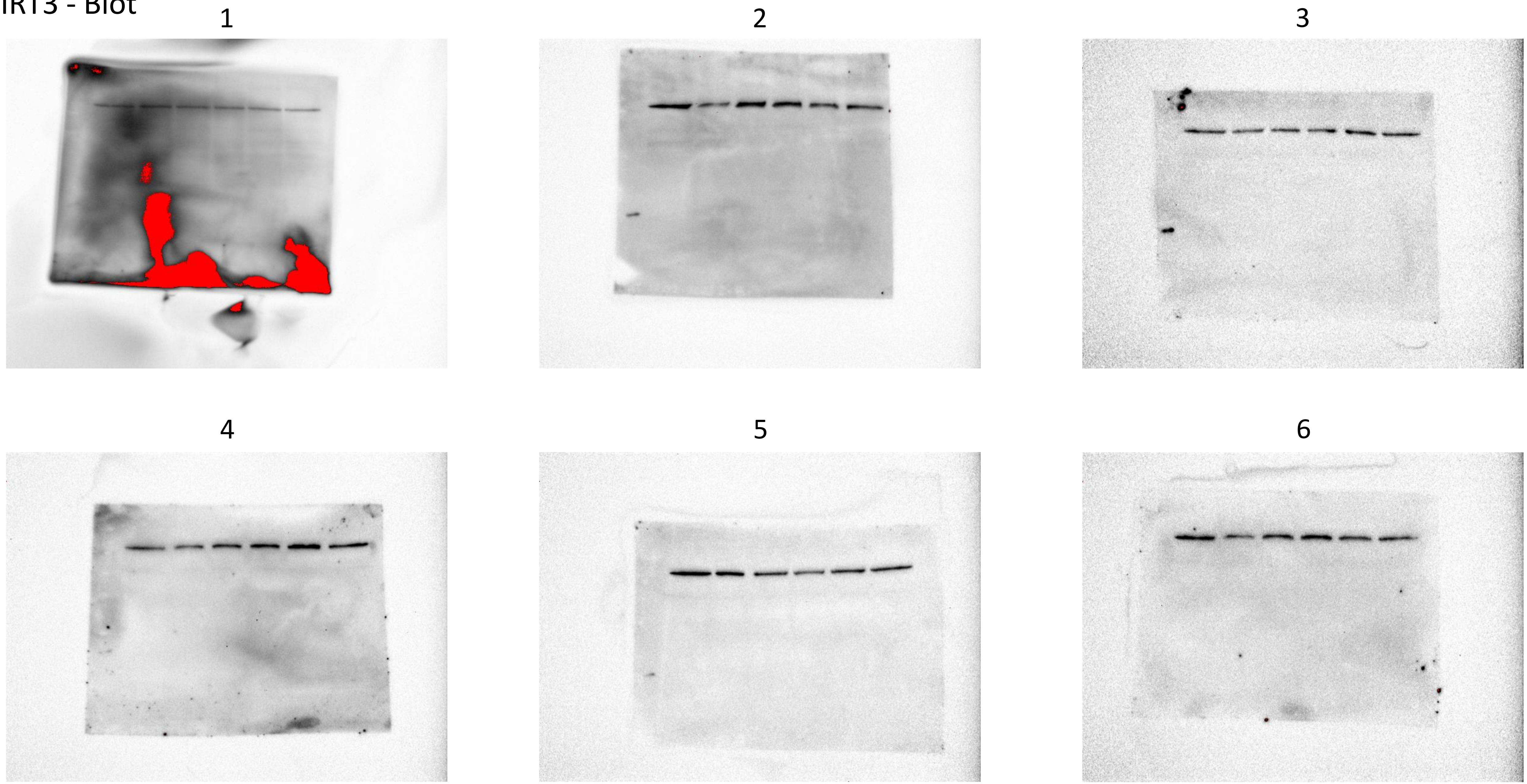

MWM: molecular weight marker

Samples order from the left in each of the 6 membranes: MWM | MTX | DOX18 | **CTRL1 | DOX1 | CTRL2 | DOX2**

PPARα - PonceauS

1

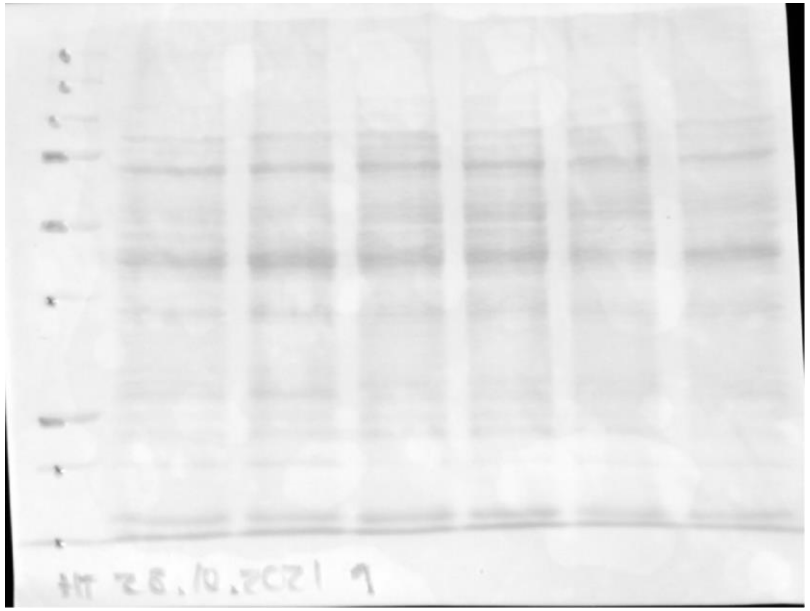

2

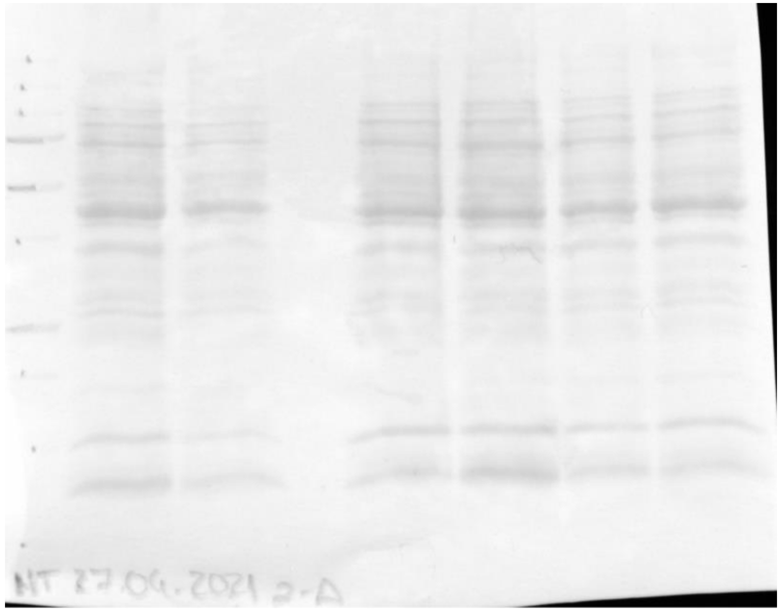

3

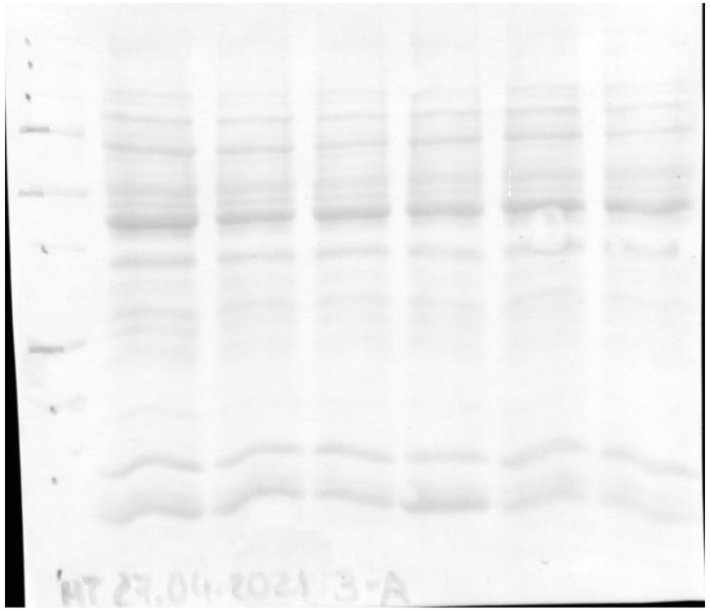

4

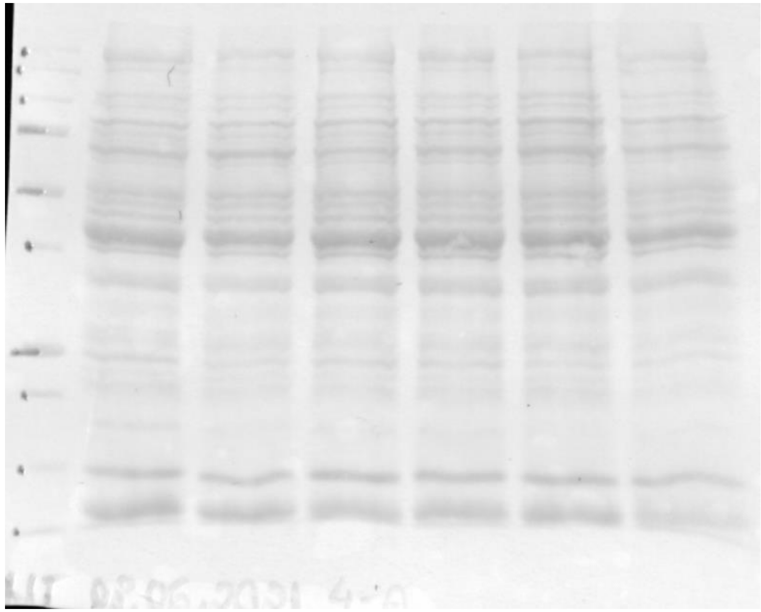

5

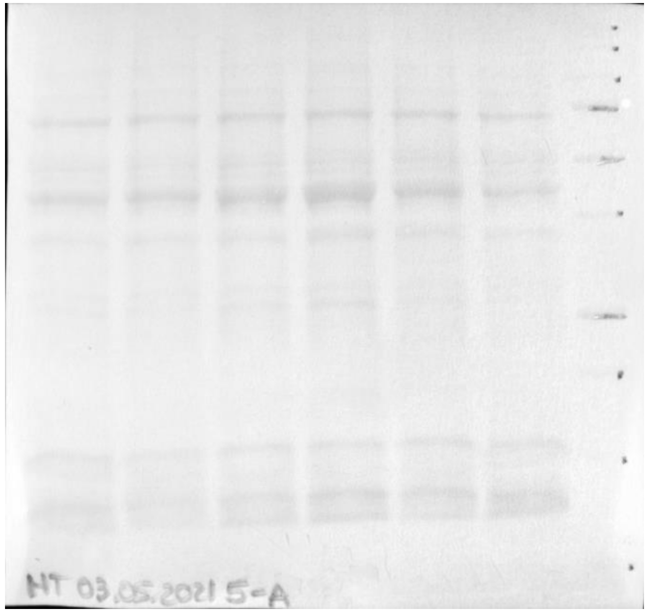

6

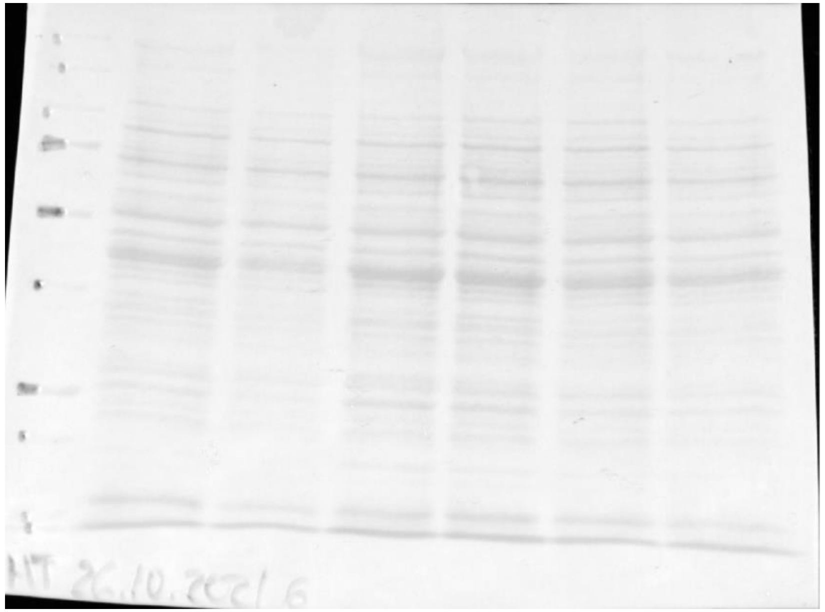

MWM: molecular weight marker

Samples order from the left in each of the 6 membranes: MWM | MTX | DOX18 | **CTRL1 | DOX1 | CTRL2 | DOX2**

PPARα - Blot

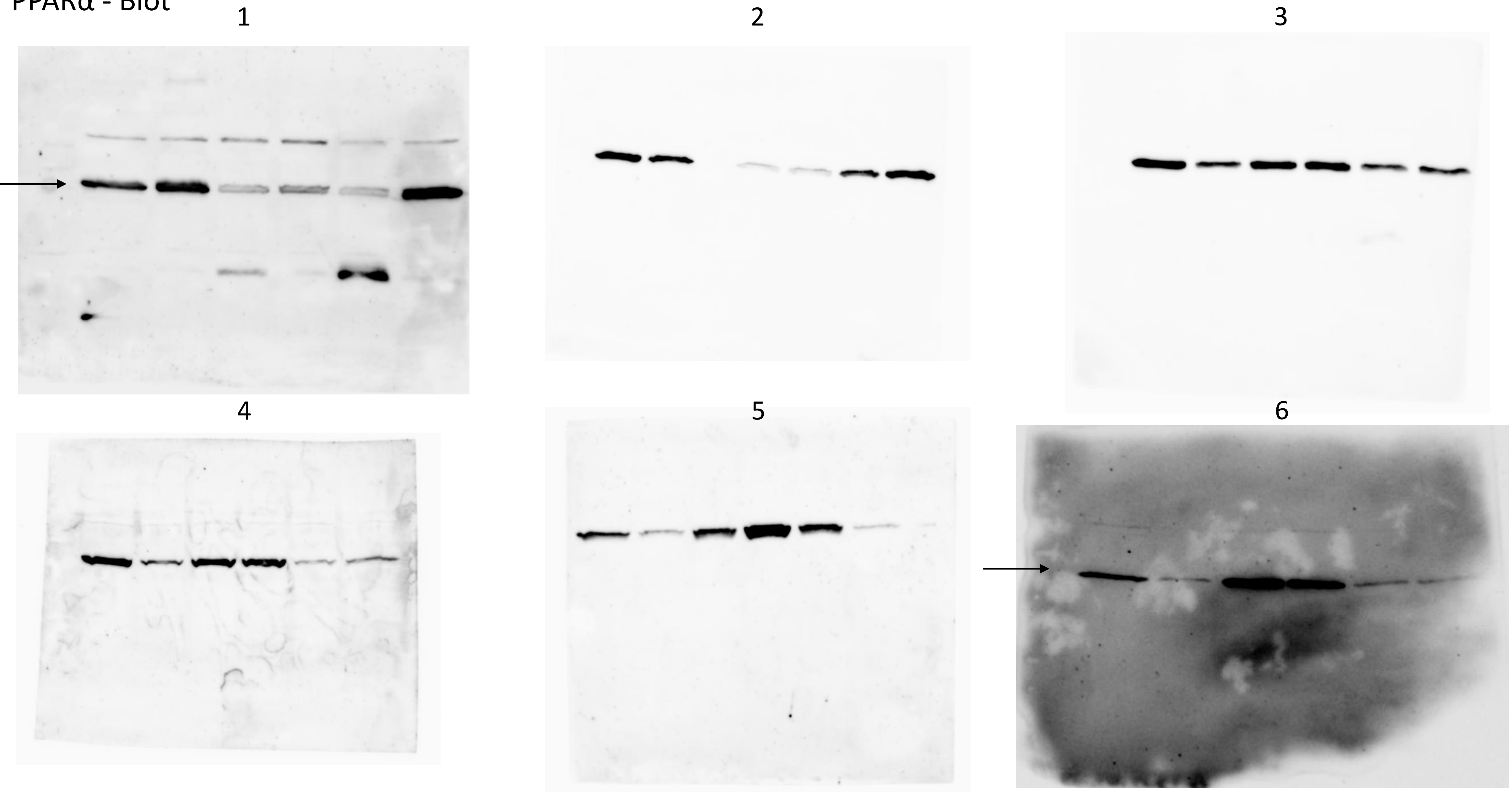

MWM: molecular weight marker

Samples order from the left in each of the 6 membranes: MWM | MTX | DOX18 | **CTRL1 | DOX1 | CTRL2 | DOX2**

PPARγ - PonceauS

1

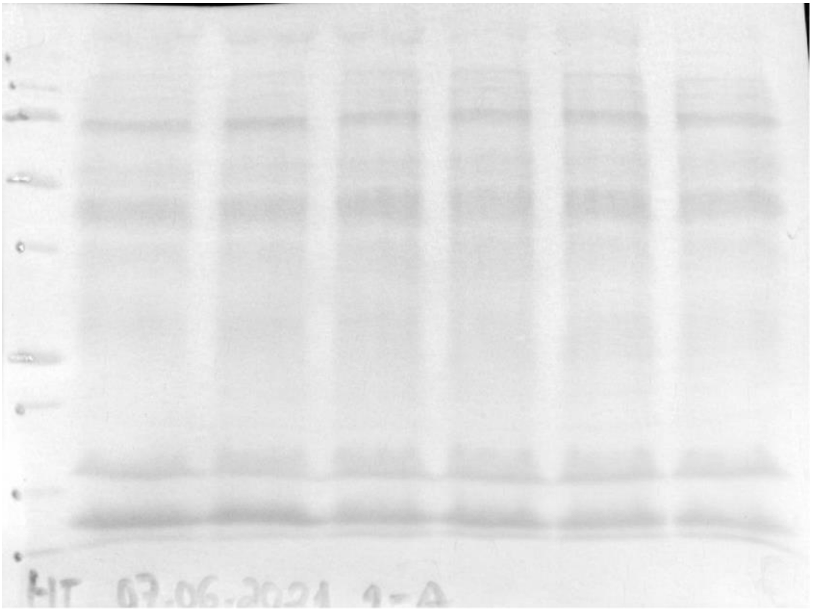

2

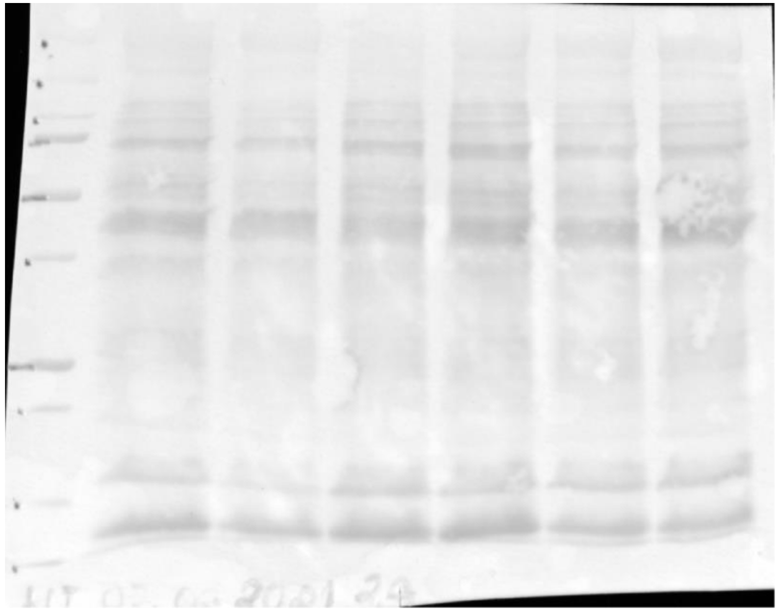

3

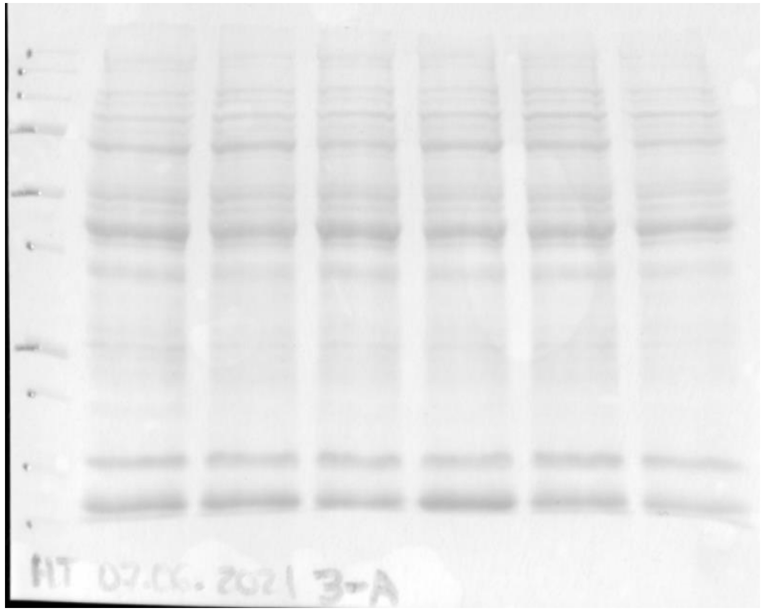

4

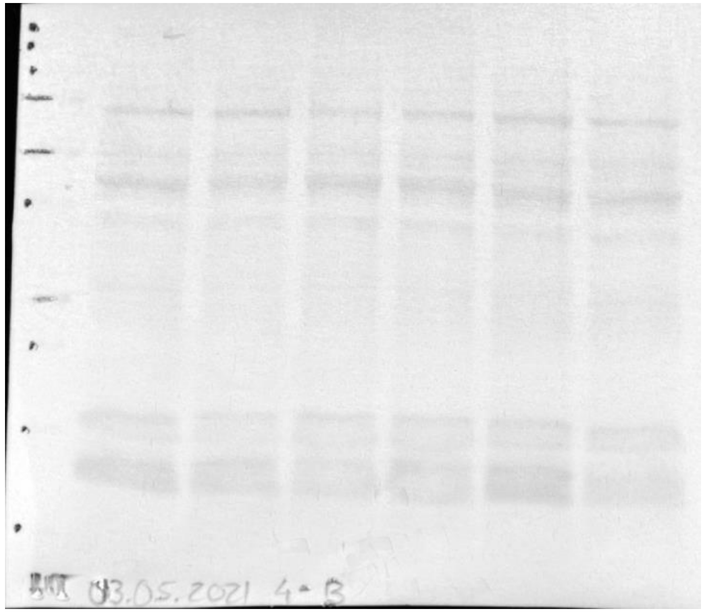

5

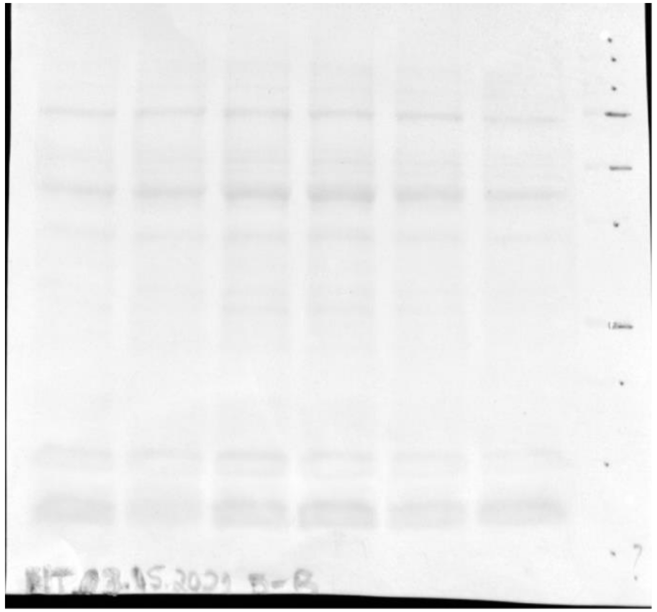

6

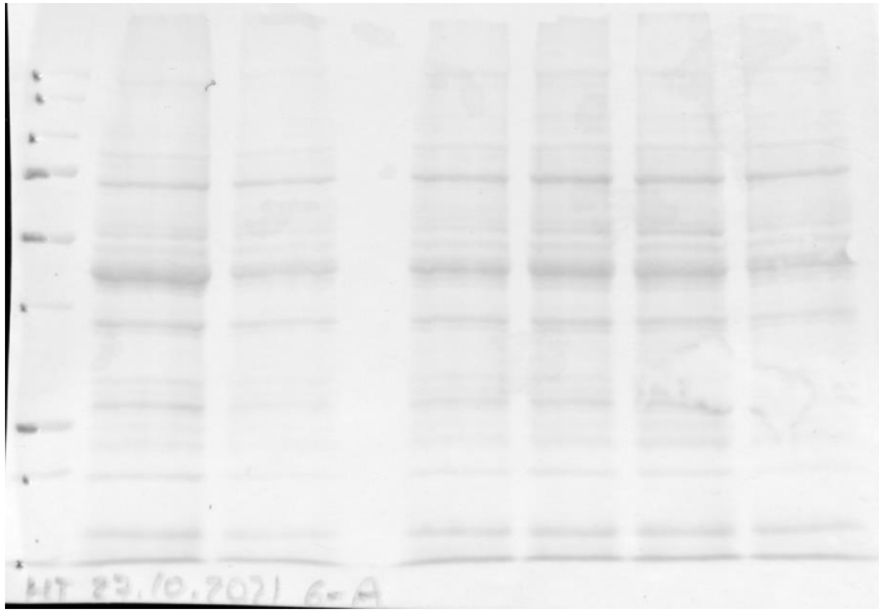

MWM: molecular weight marker

Samples order from the left in each of the 6 membranes: MWM | MTX | DOX18 | **CTRL1 | DOX1 | CTRL2 | DOX2**

PPARγ - Blot

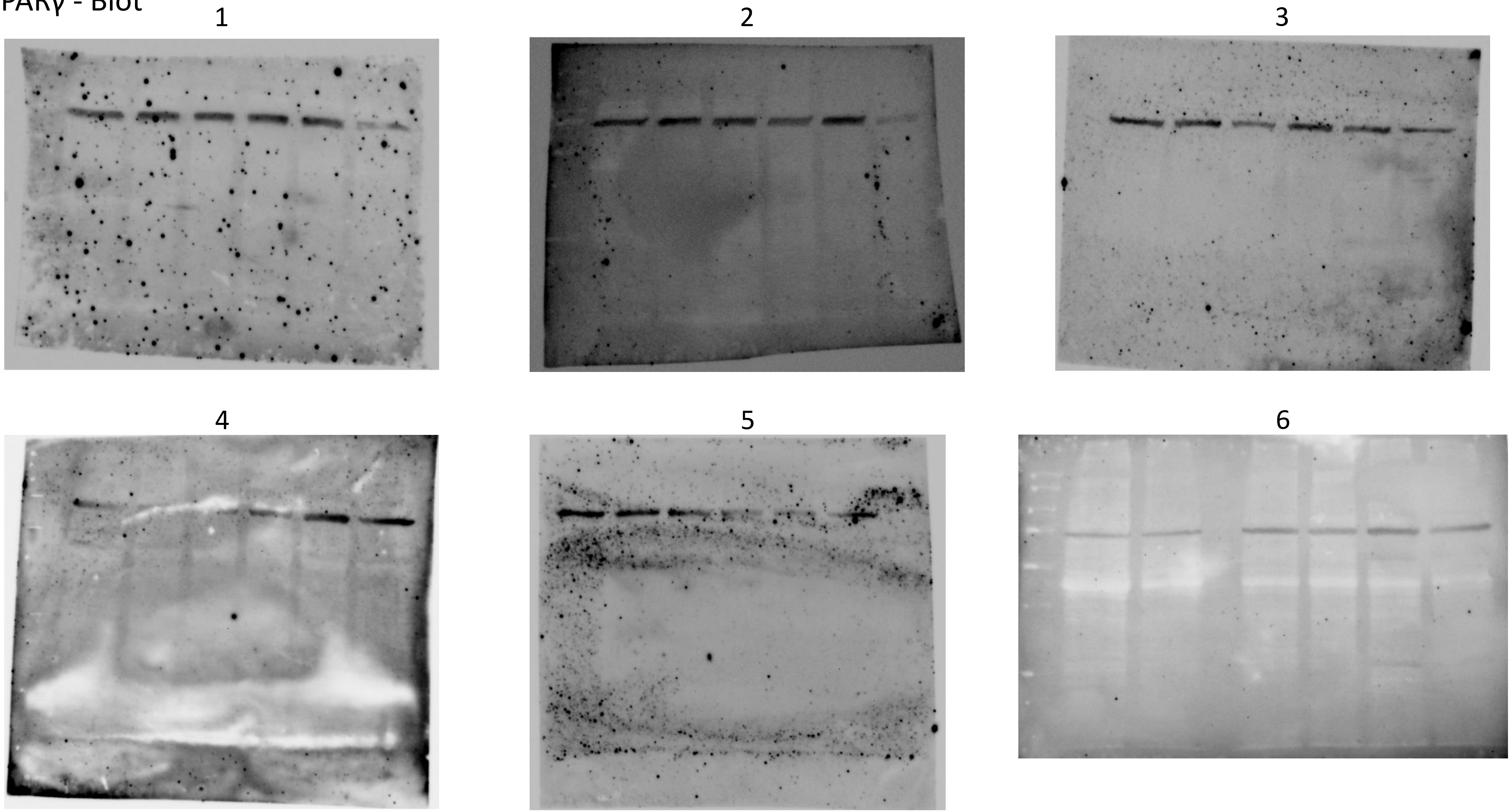

MWM: molecular weight marker

Samples order from the left in each of the 6 membranes: MWM | MTX | DOX18 | **CTRL1 | DOX1 | CTRL2 | DOX2**

GSK-3β - PonceauS

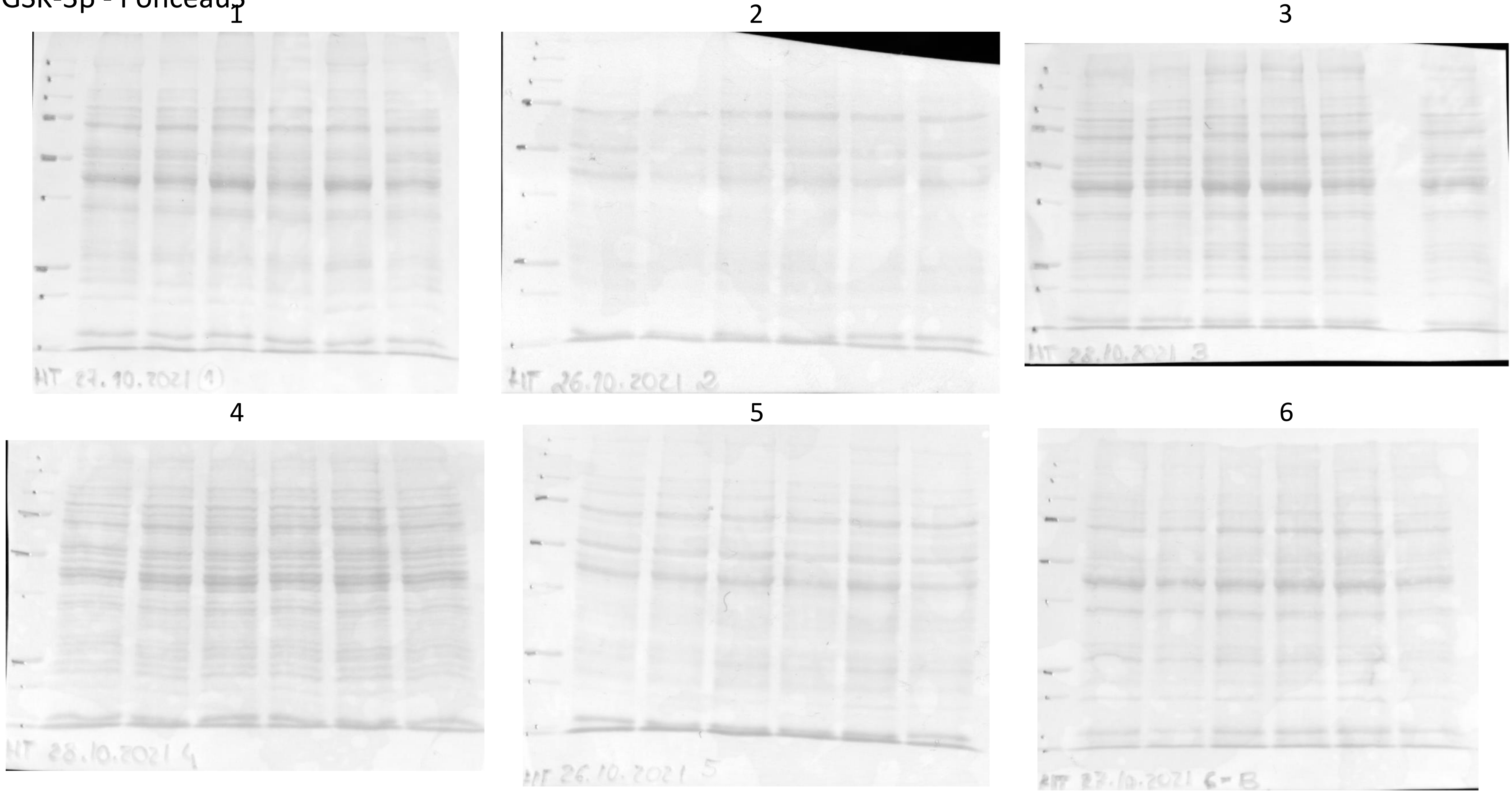

MWM: molecular weight marker

Samples order from the left in each of the 6 membranes: MWM | MTX | DOX18 | **CTRL1 | DOX1 | CTRL2 | DOX2**

GSK-3β - Blot

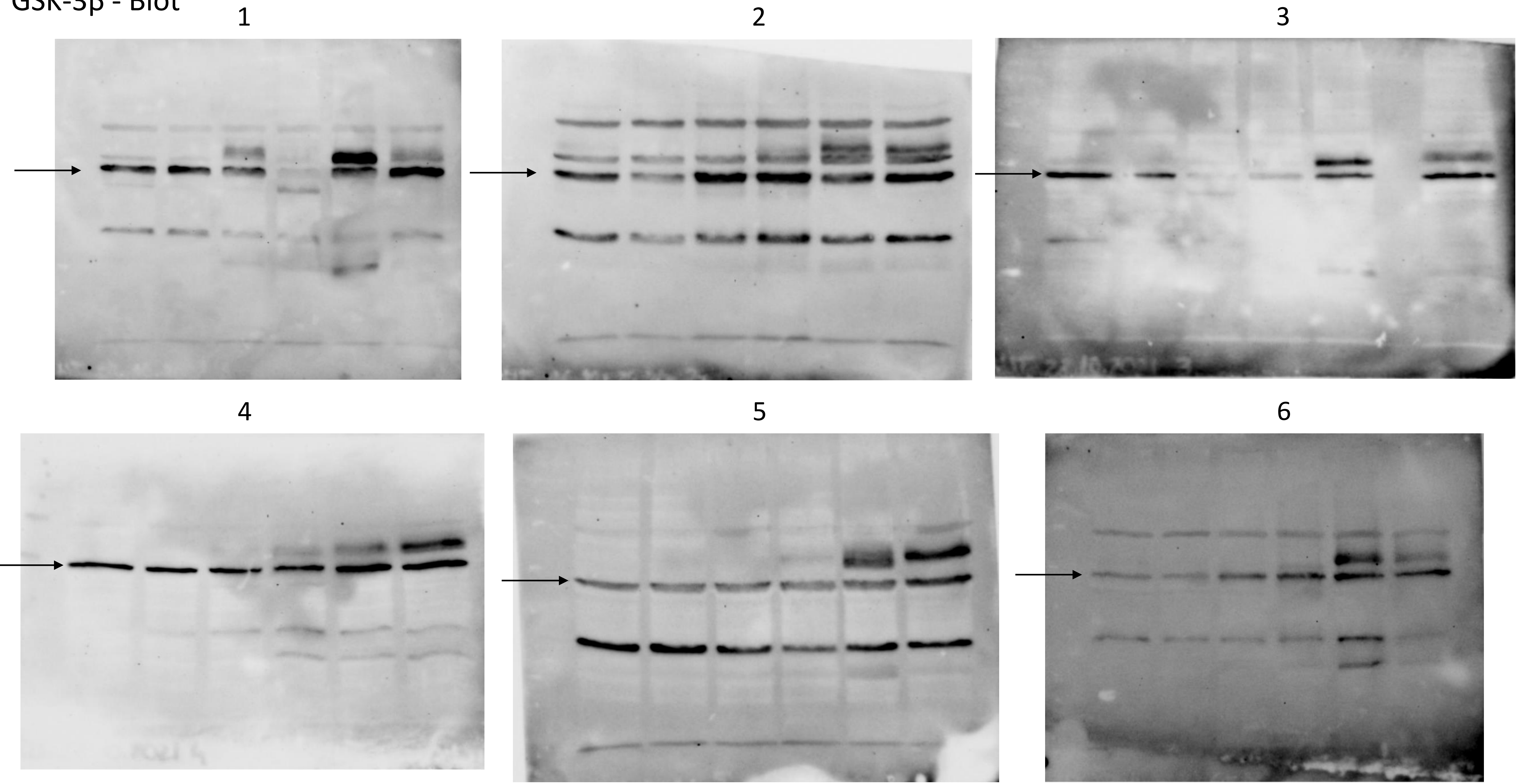

MWM: molecular weight marker

Samples order from the left in each of the 6 membranes: MWM | MTX | DOX18 | **CTRL1 | DOX1 | CTRL2 | DOX2**

AMPK - PonceauS

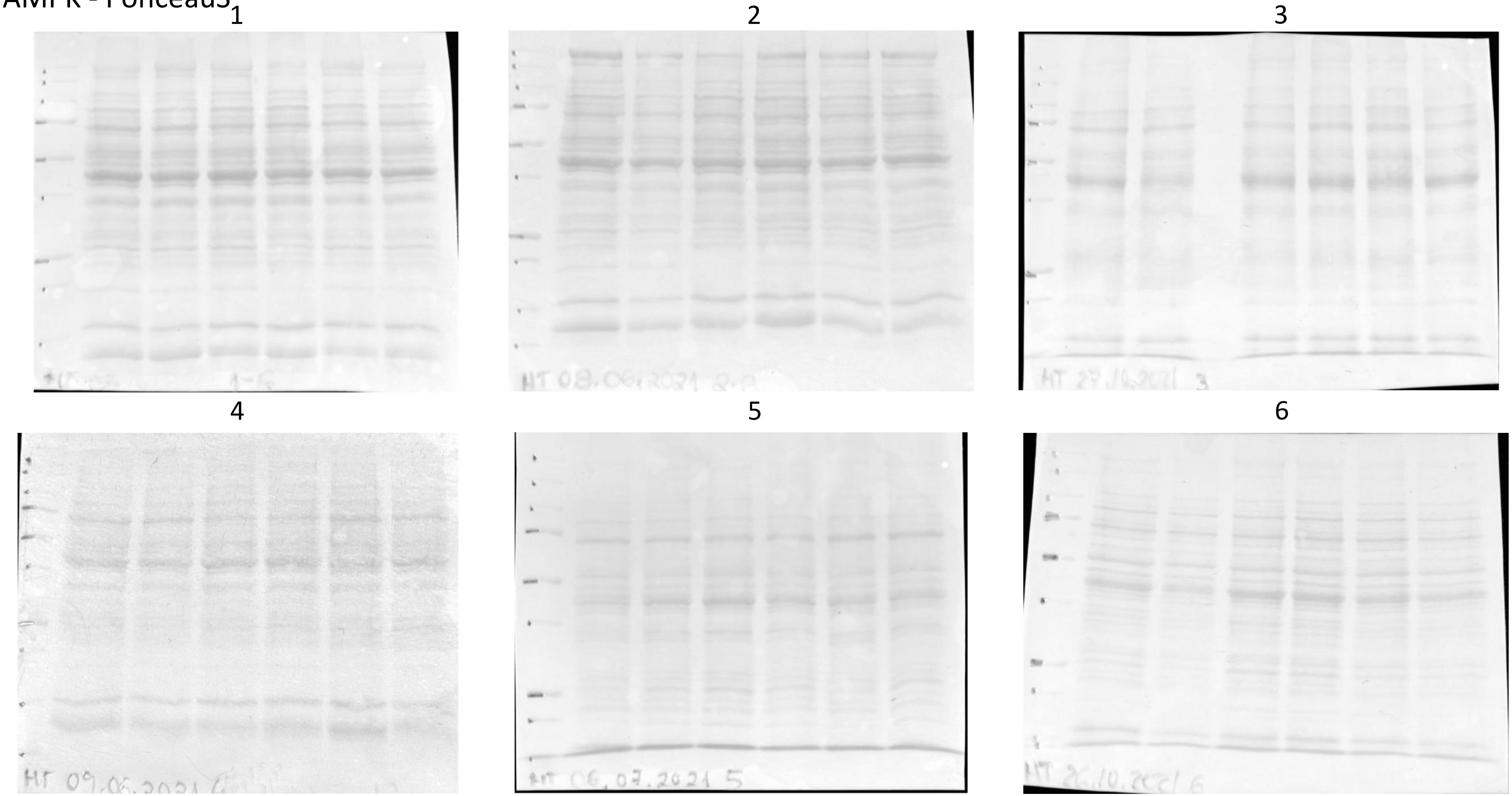

MWM: molecular weight marker

Samples order from the left in each of the 6 membranes: MWM | MTX | DOX18 | **CTRL1 | DOX1 | CTRL2 | DOX2**

AMPK - Blot

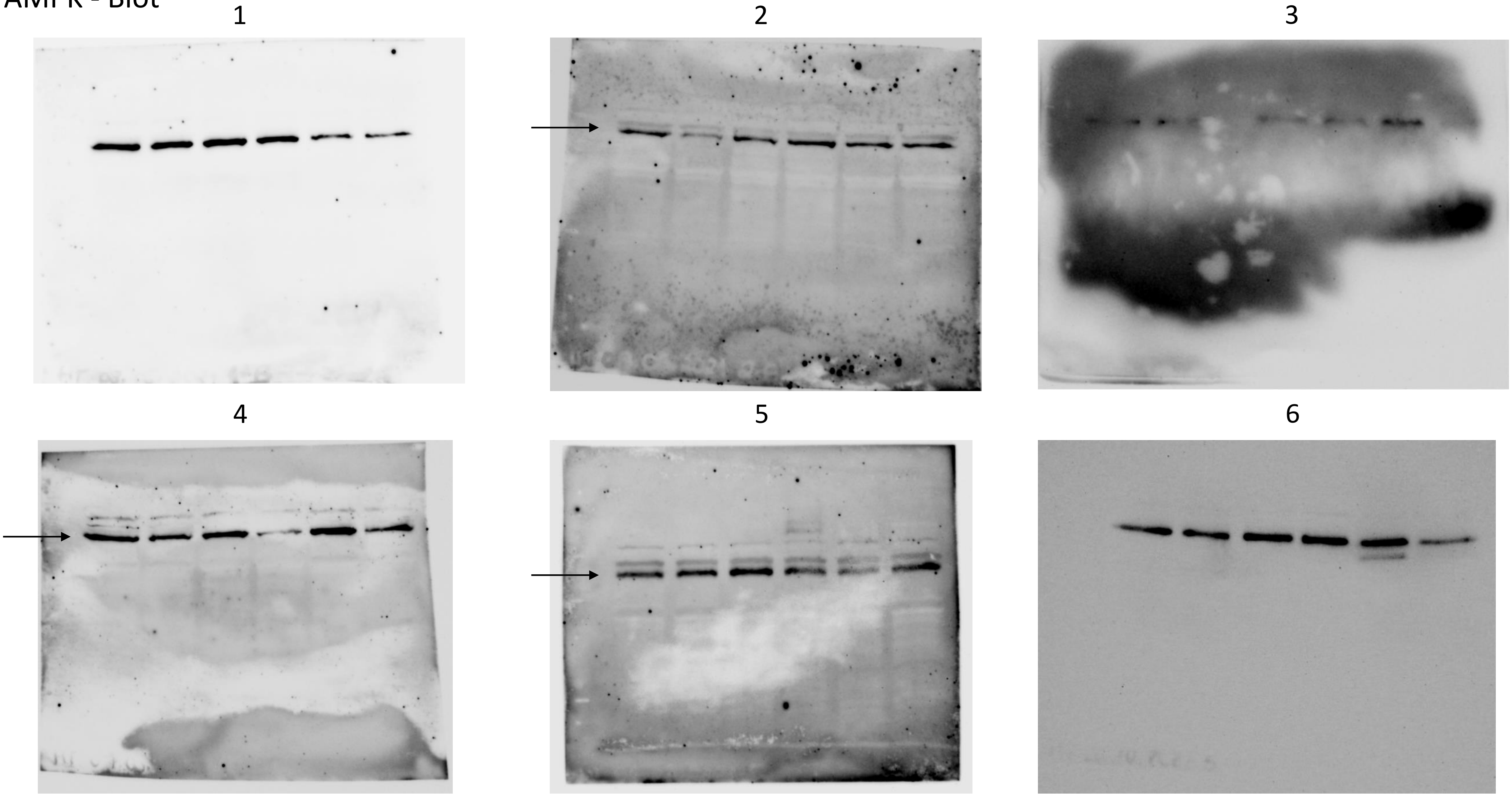

MWM: molecular weight marker

Samples order from the left in each of the 6 membranes: MWM | MTX | DOX18 | **CTRL1 | DOX1 | CTRL2 | DOX2**

pAMPK - PonceauS

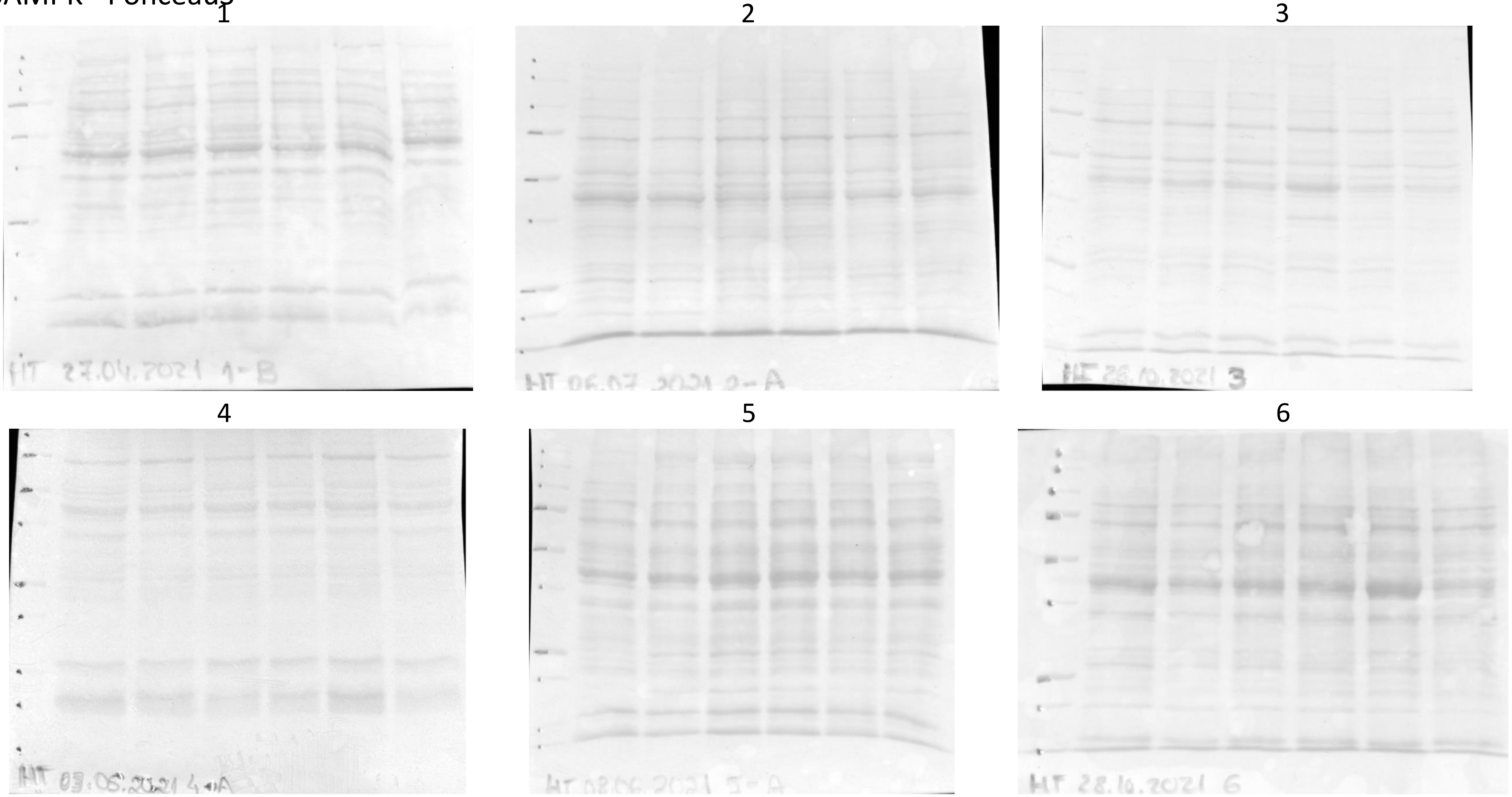

MWM: molecular weight marker

Samples order from the left in each of the 6 membranes: MWM | MTX | DOX18 | **CTRL1 | DOX1 | CTRL2 | DOX2**

pAMPK - Blot

1

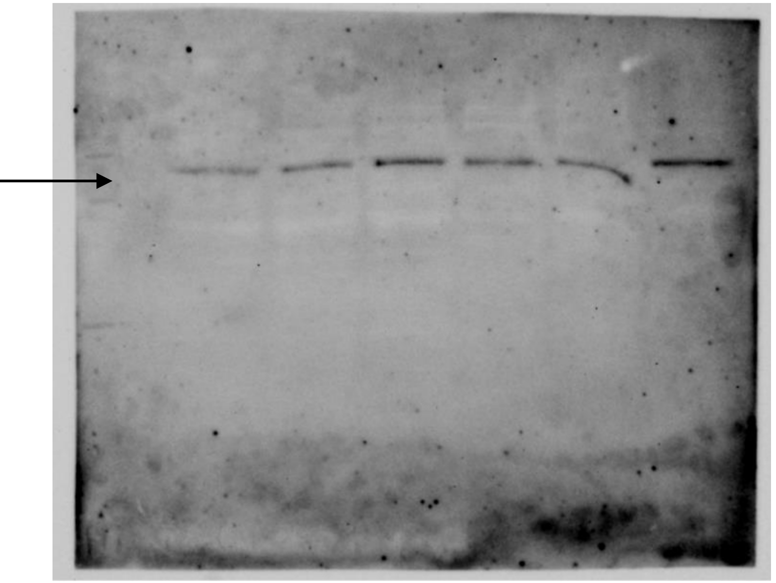

2

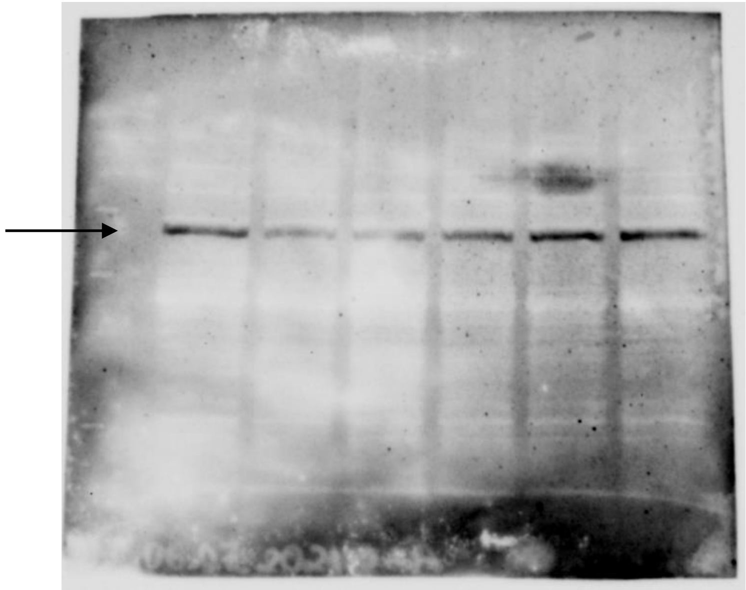

3

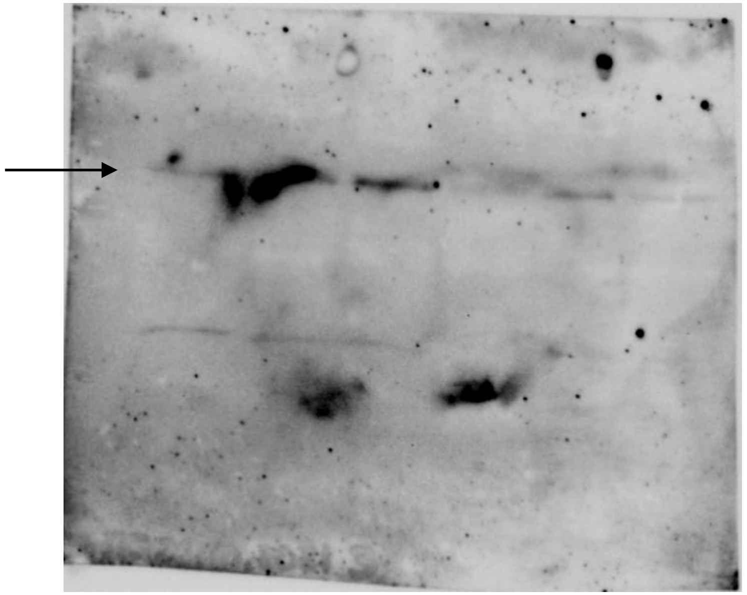

4

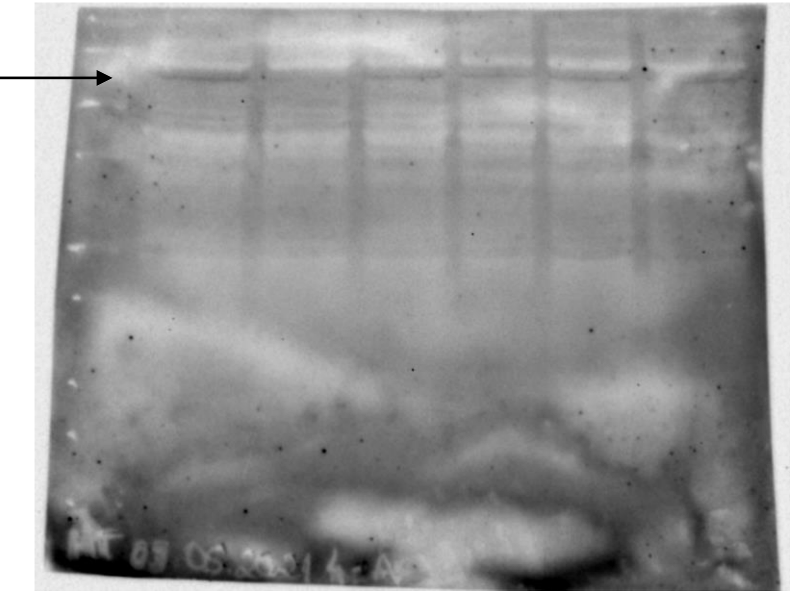

5

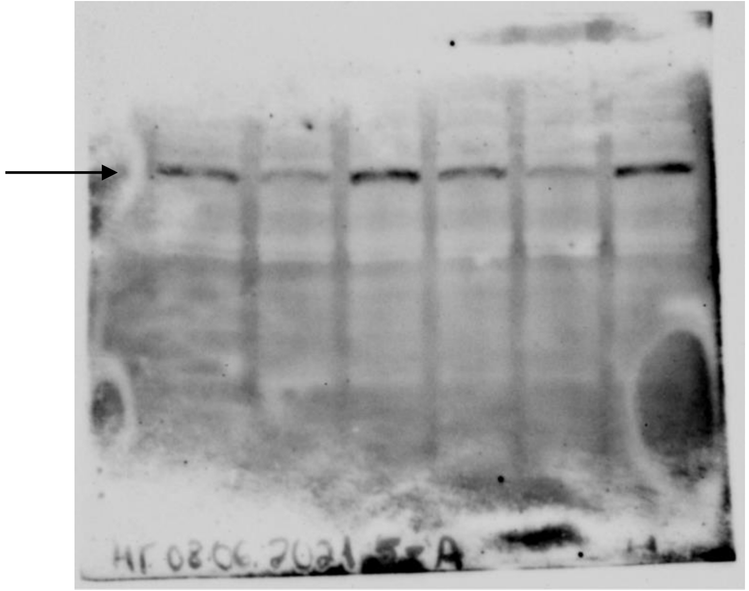

6

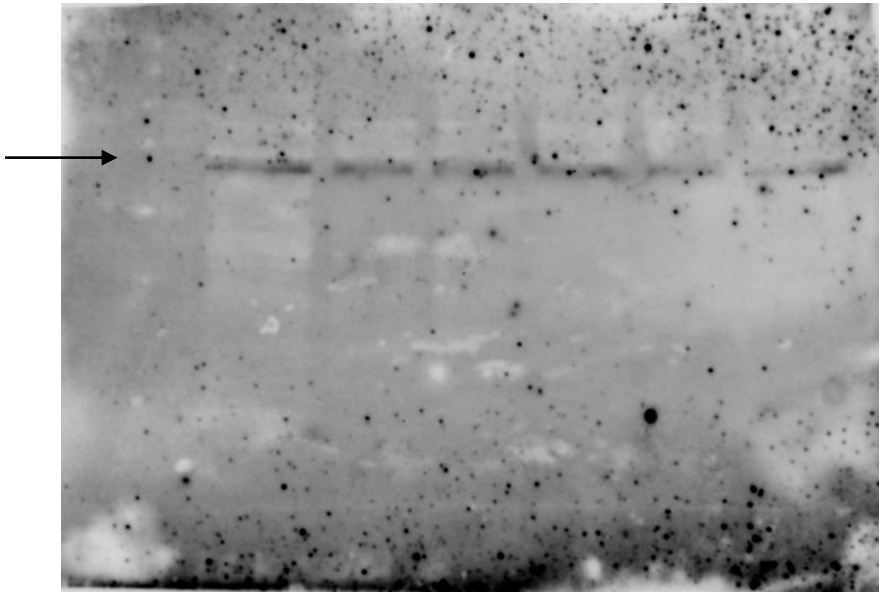

MWM: molecular weight marker

Samples order from the left in each of the 6 membranes: MWM | MTX | DOX18 | **CTRL1 | DOX1 | CTRL2 | DOX2**

DNP (protein carbonyl levels) - cardiac

Blot

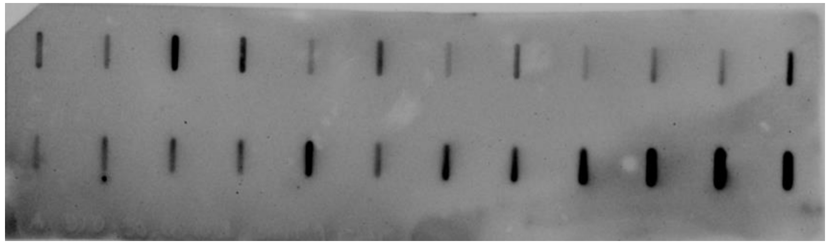

Samples order from upper left corner: CTRL1-1 | DOX1-1 | CTRL2-1 | DOX2-1 | CTRL1-2 | DOX1-2 | CTRL2-2 | DOX2-2 | CTRL1-3 | DOX1-3 | CTRL2-3 | DOX2-3
CTRL1-4 | DOX1-4 | CTRL2-4 | DOX2-4 | CTRL1-5 | DOX1-5 | CTRL2-5 | DOX2-5 | CTRL1-6 | DOX1-6 | CTRL2-6 | DOX2-6

Nitrotyrosine - cardiac

PonceauS

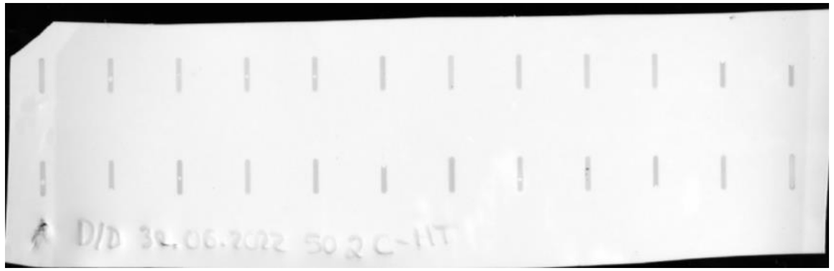

Blot

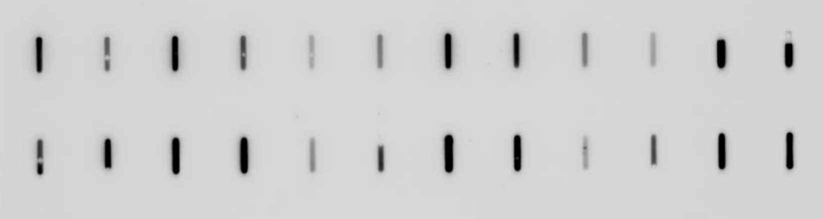

Samples order from upper left corner: CTRL1-1 | DOX1-1 | CTRL2-1 | DOX2-1 | CTRL1-2 | DOX1-2 | CTRL2-2 | DOX2-2 | CTRL1-3 | DOX1-3 | CTRL2-3 | DOX2-3
CTRL1-4 | DOX1-4 | CTRL2-4 | DOX2-4 | CTRL1-5 | DOX1-5 | CTRL2-5 | DOX2-5 | CTRL1-6 | DOX1-6 | CTRL2-6 | DOX2-6

MnSOD - PonceauS

1

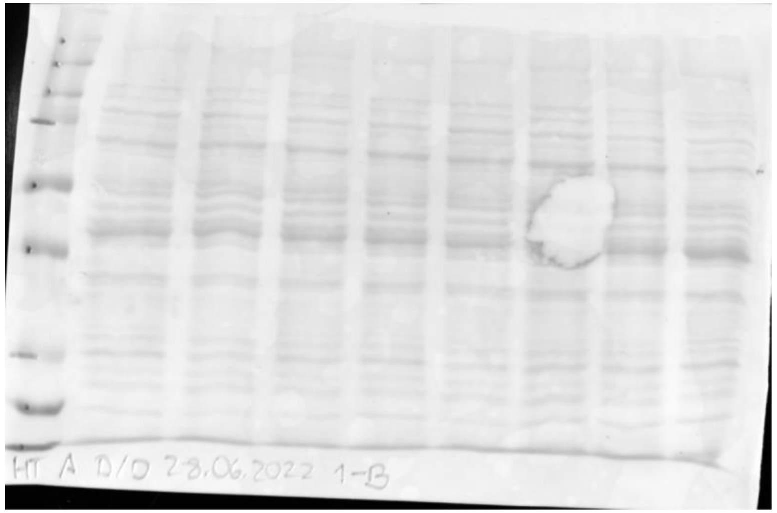

2

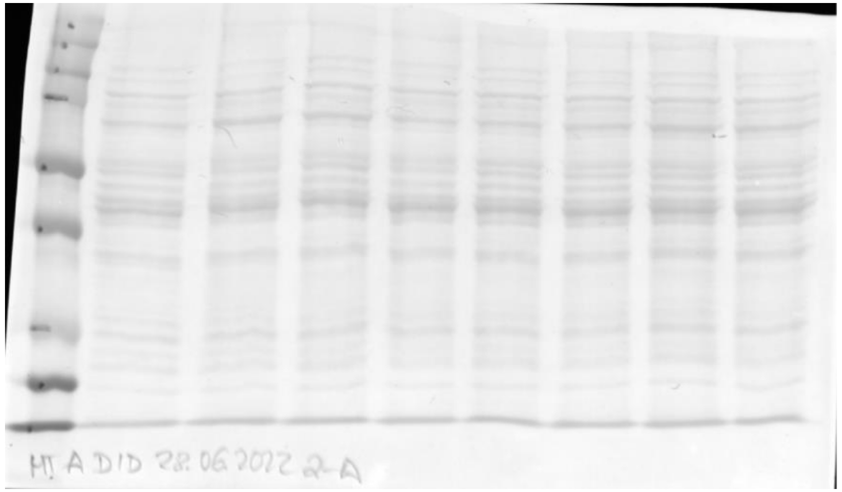

3

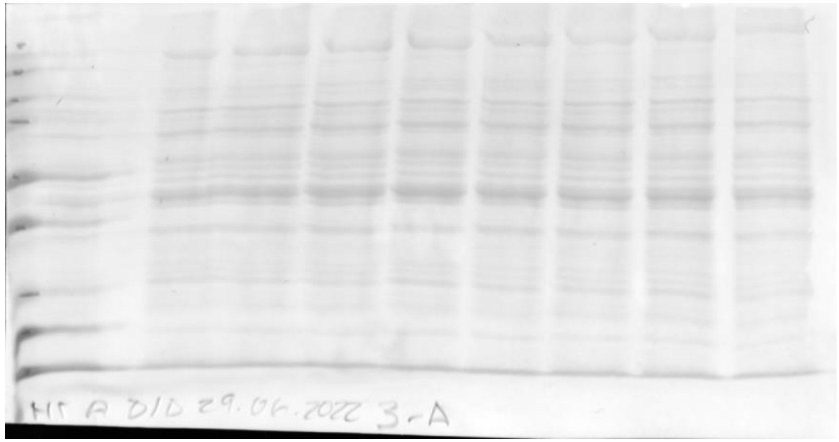

Samples order from the left in each of the 3 membranes: MWM | CTRL1-1 | DOX1-1 | CTRL2-1 | DOX2-1 | CTRL1-2 | DOX1-2 | CTRL2-2 | DOX2-2
MWM: molecular weight marker

MnSOD - Blot

1

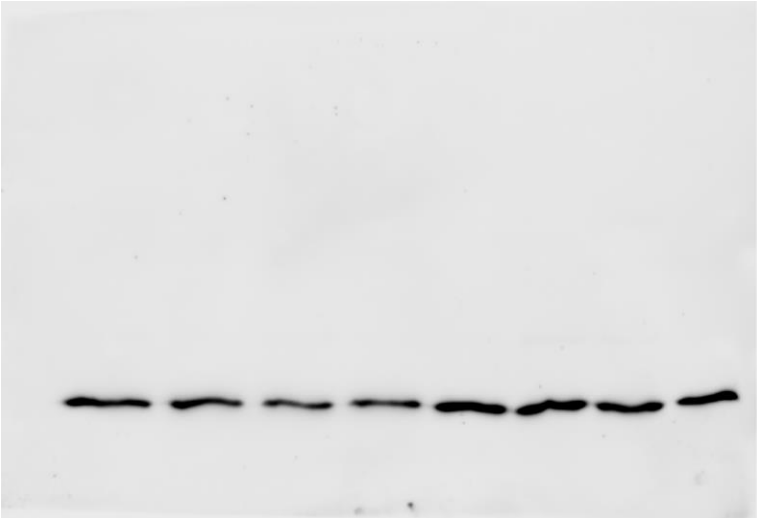

2

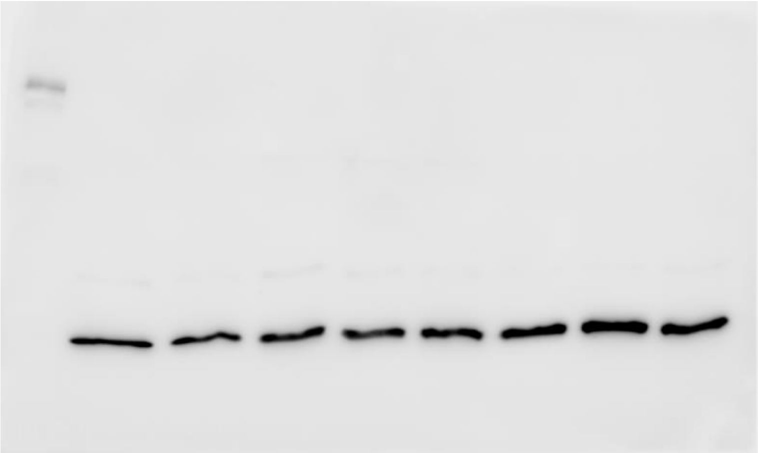

3

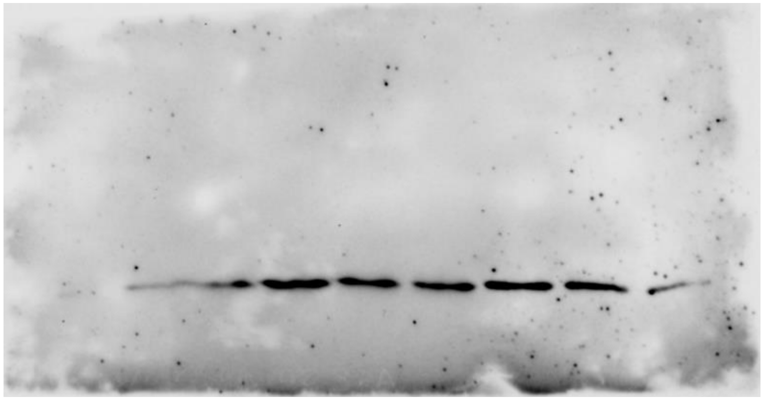

PGC-1α - PonceauS

1

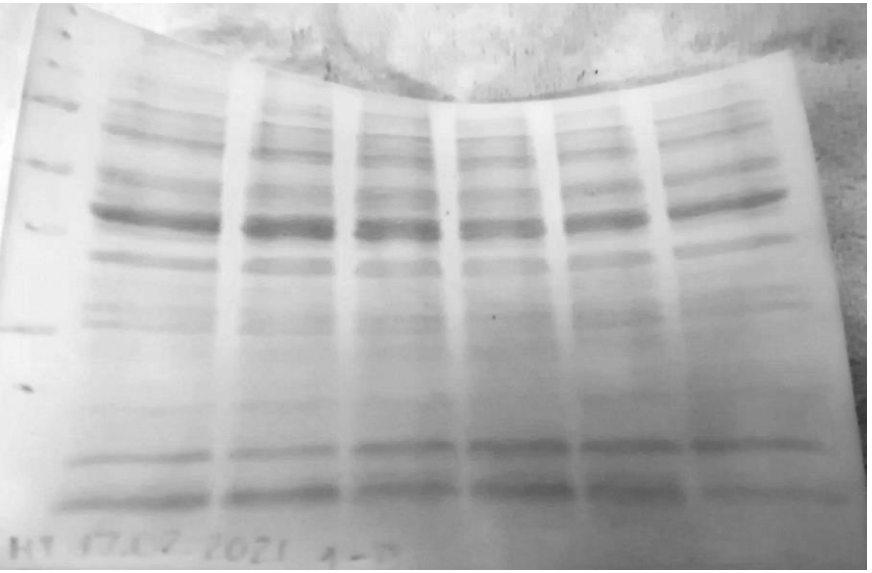

2

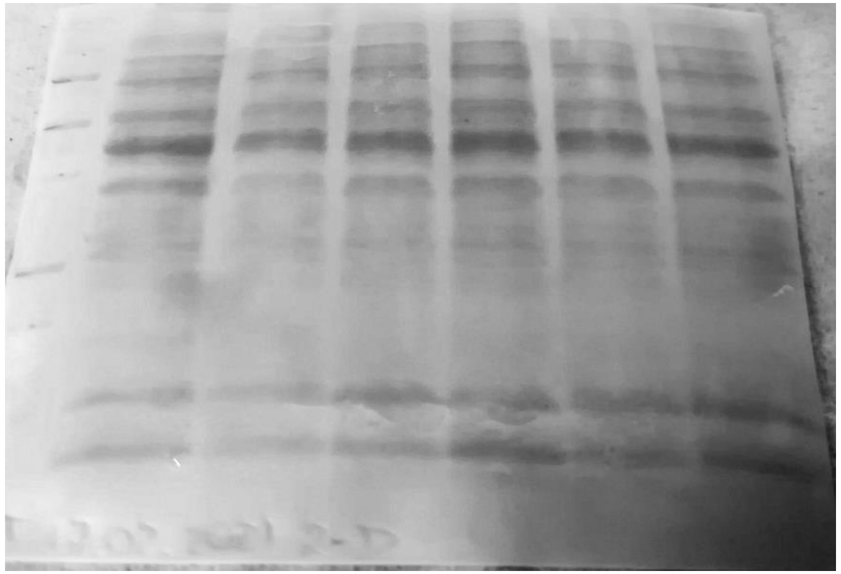

3

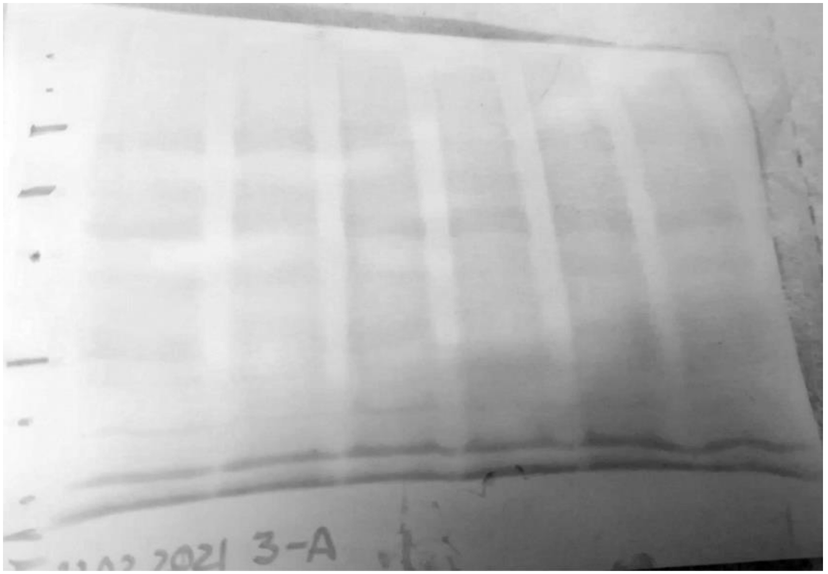

4

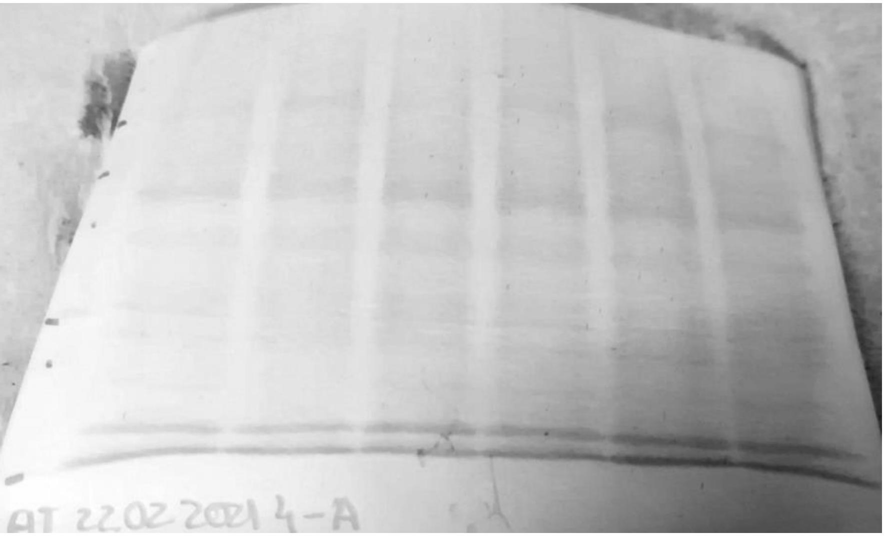

5

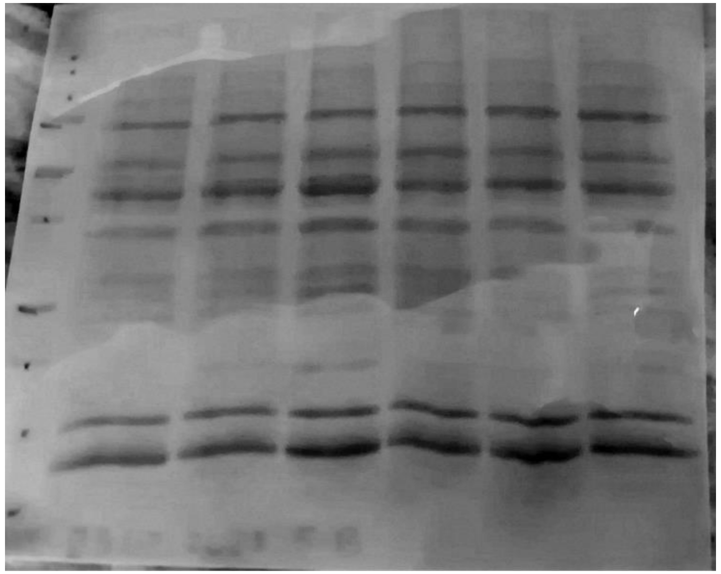

6

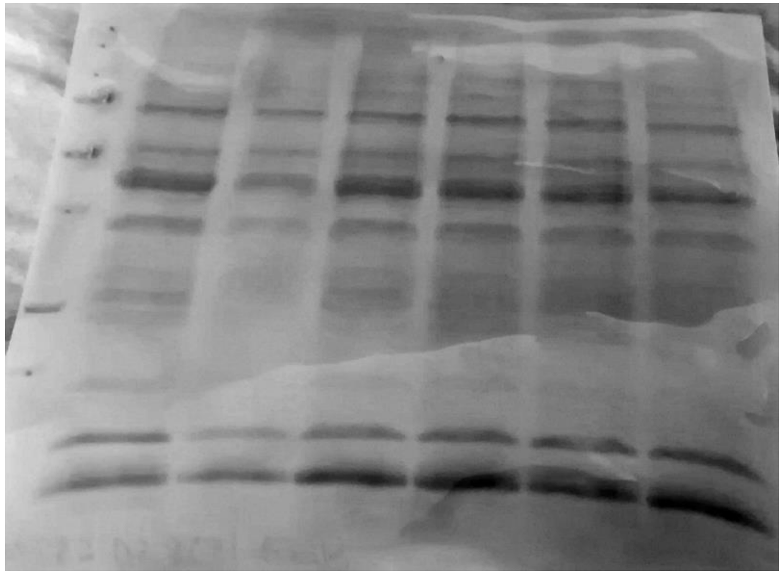

MWM: molecular weight marker

Samples order from the left in each of the 6 membranes: MWM | MTX | DOX18 | **CTRL1 | DOX1 | CTRL2 | DOX2**

PGC-1α - Blot

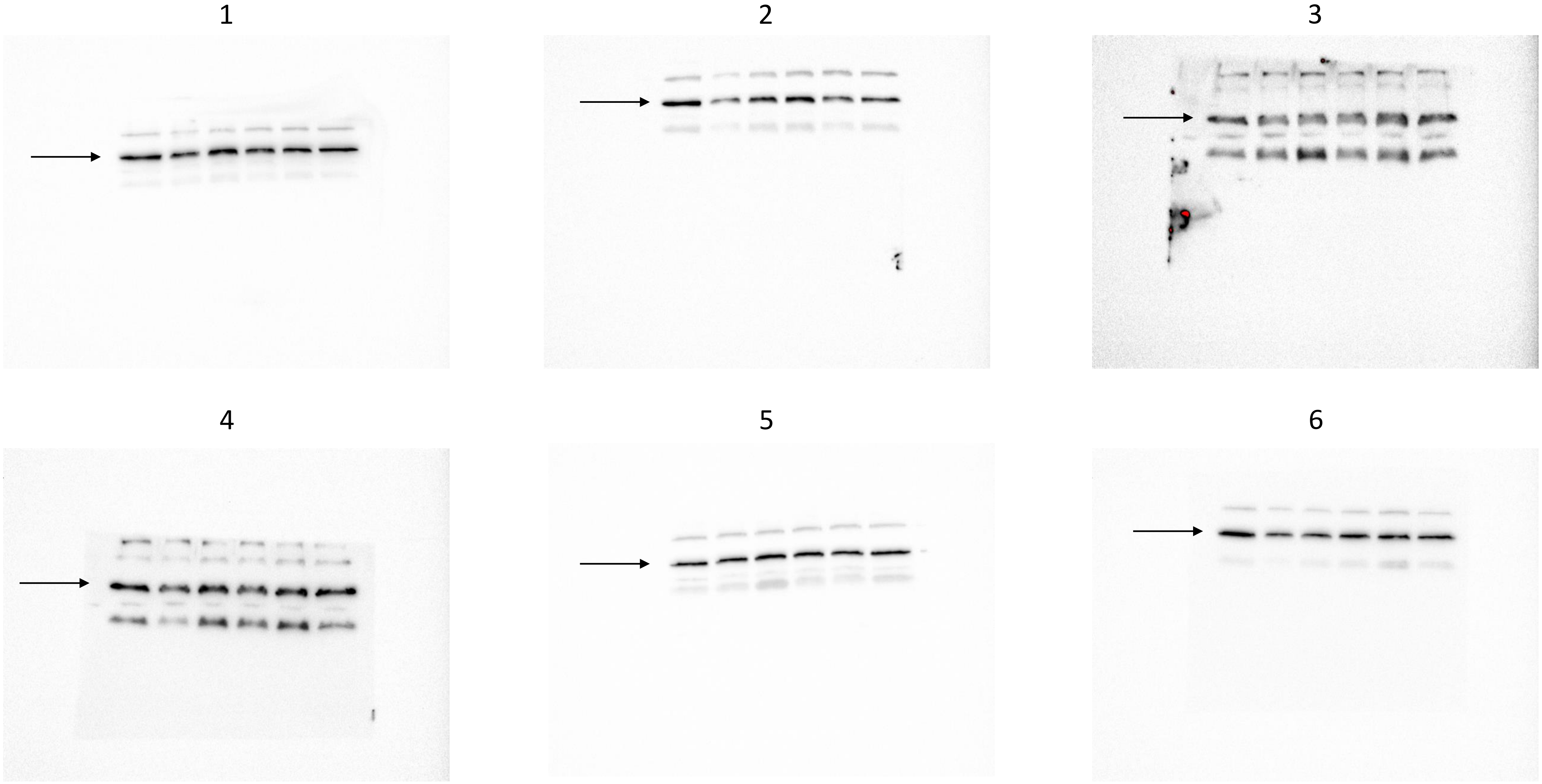

MWM: molecular weight marker

Samples order from the left in each of the 6 membranes: MWM | MTX | DOX18 | **CTRL1 | DOX1 | CTRL2 | DOX2**

Tfam - PonceauS

1

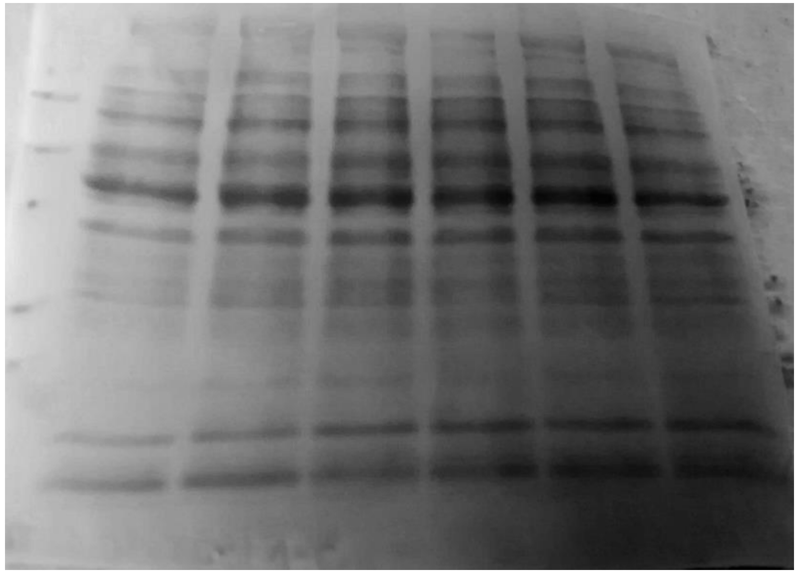

2

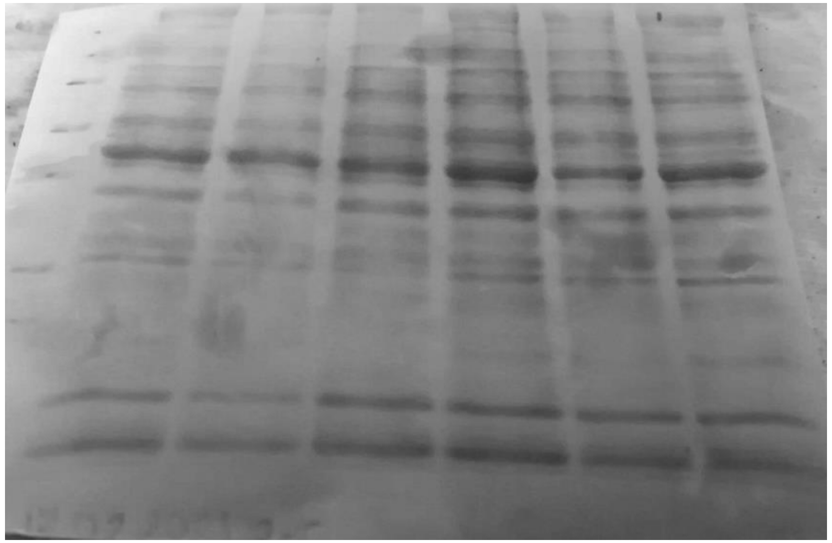

3

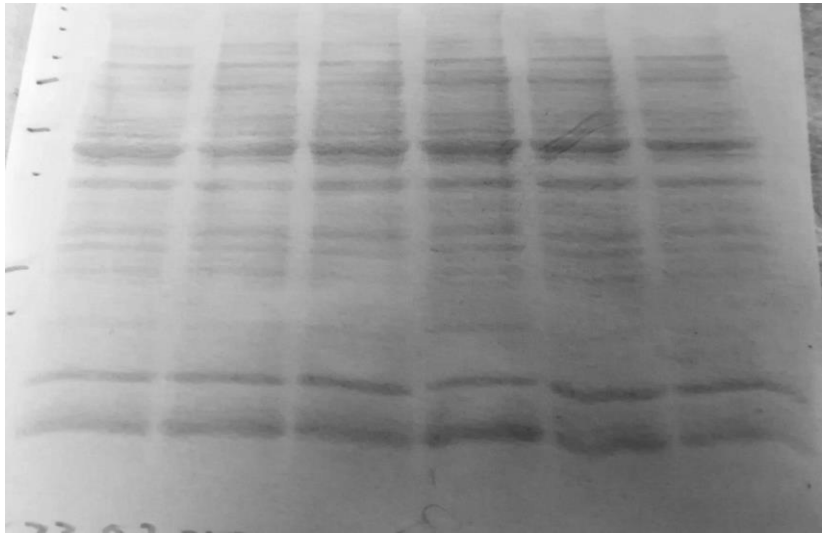

4

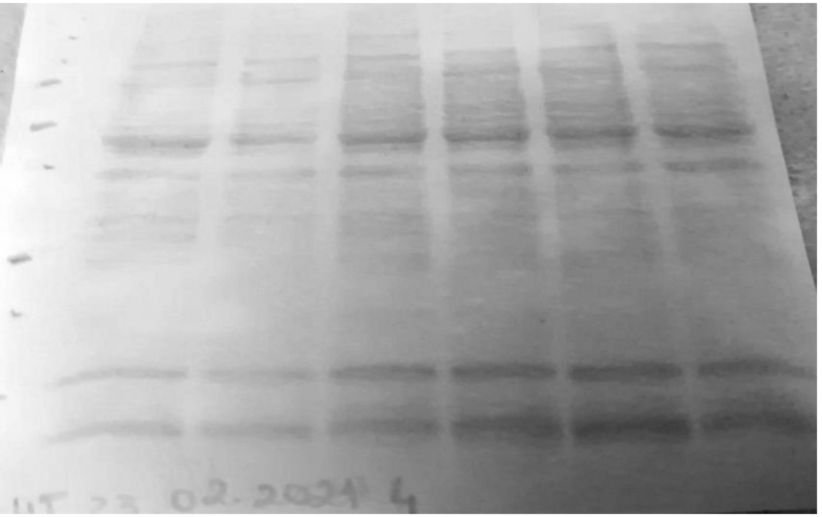

5

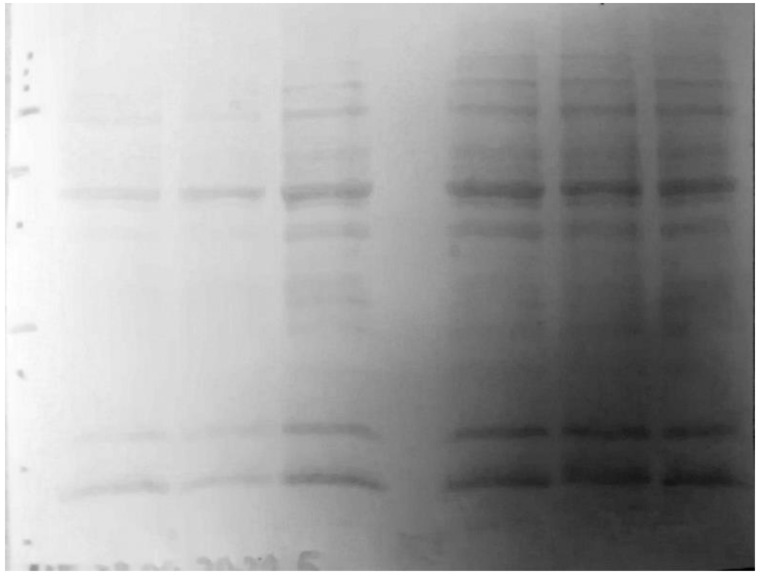

6

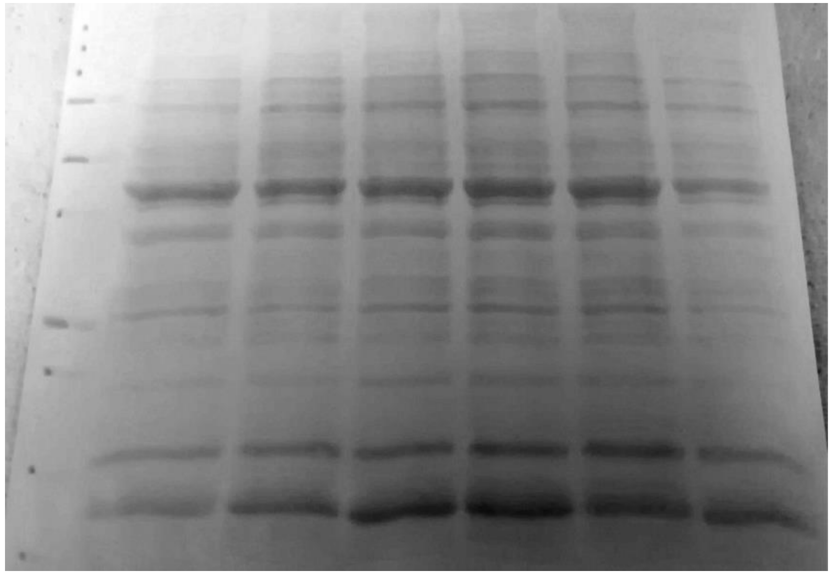

MWM: molecular weight marker

Samples order from the left in each of the 6 membranes: MWM | MTX | DOX18 | **CTRL1 | DOX1 | CTRL2 | DOX2**

Tfam - Blot

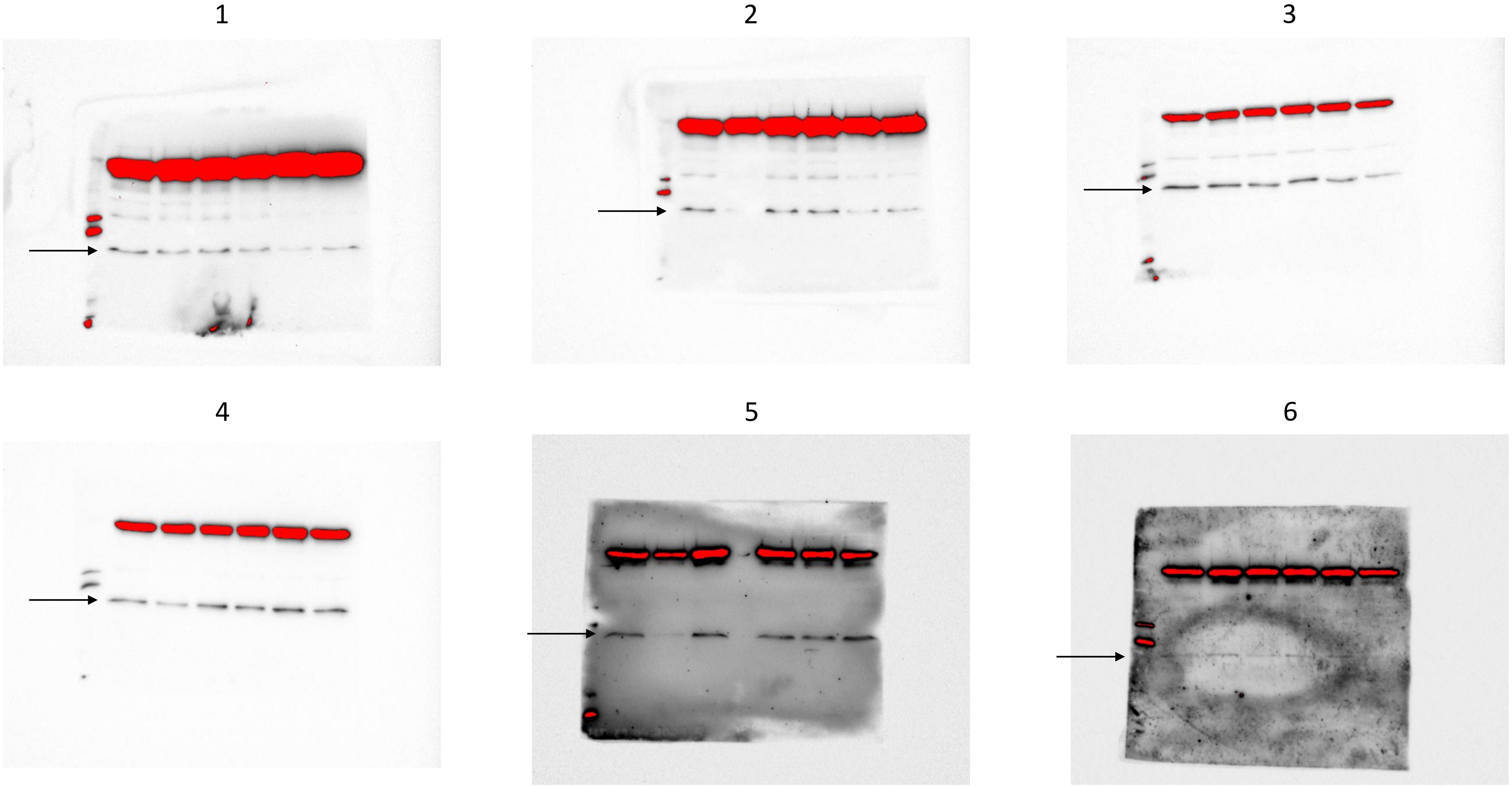

MWM: molecular weight marker

Samples order from the left in each of the 6 membranes: MWM | MTX | DOX18 | **CTRL1 | DOX1 | CTRL2 | DOX2**

Mfn1 - PonceauS

1

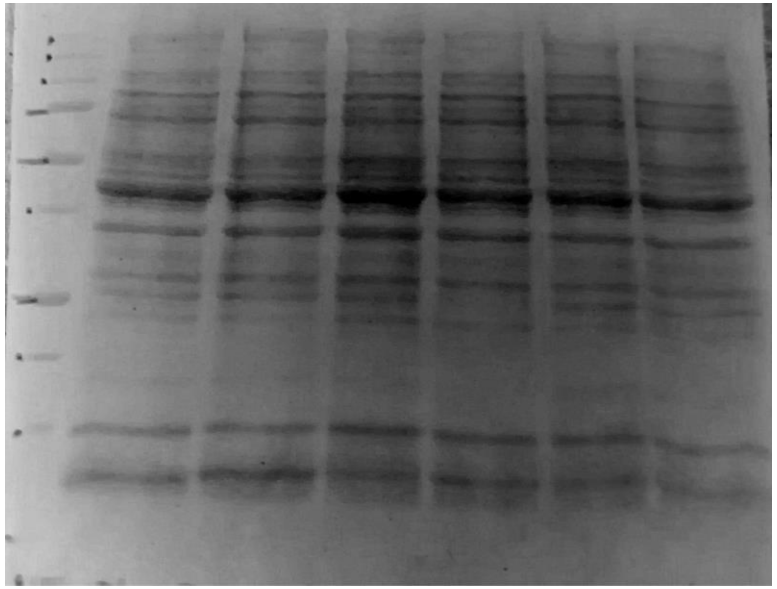

2

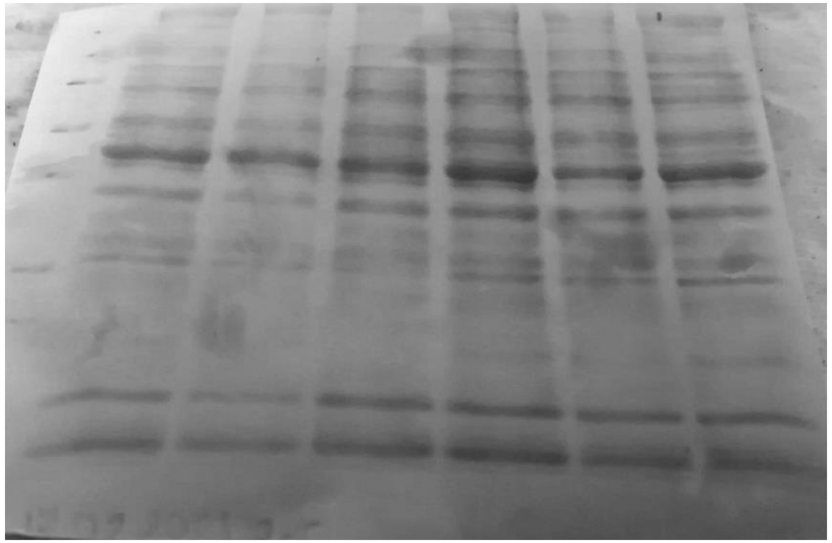

3

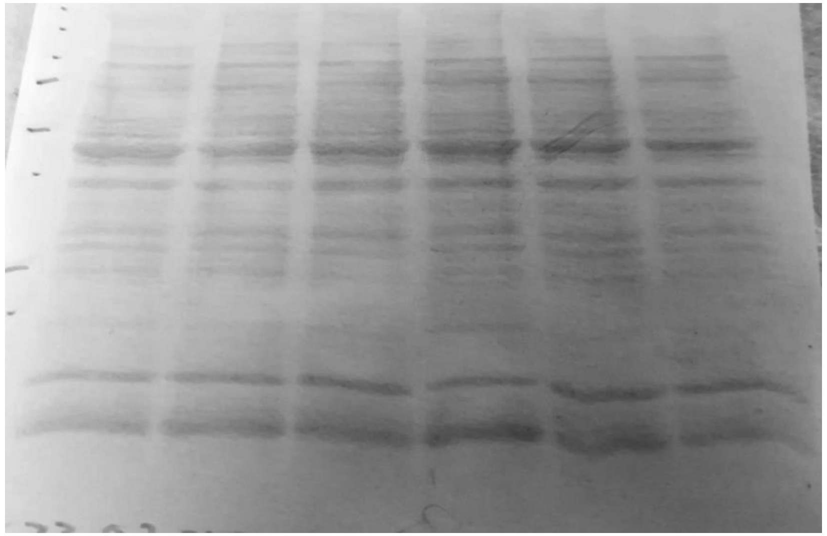

4

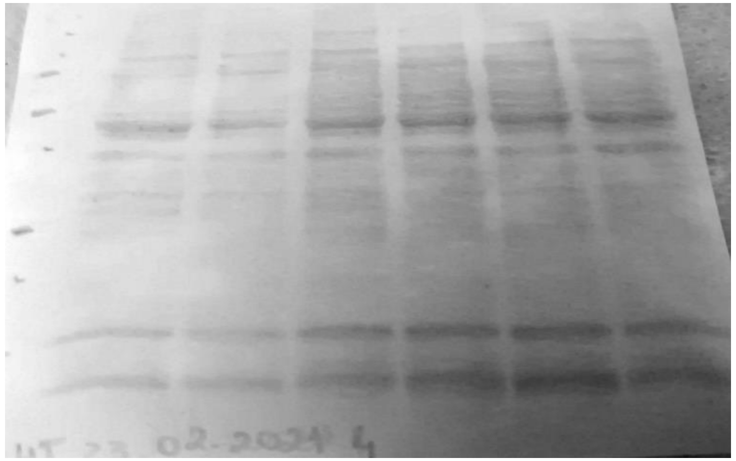

5

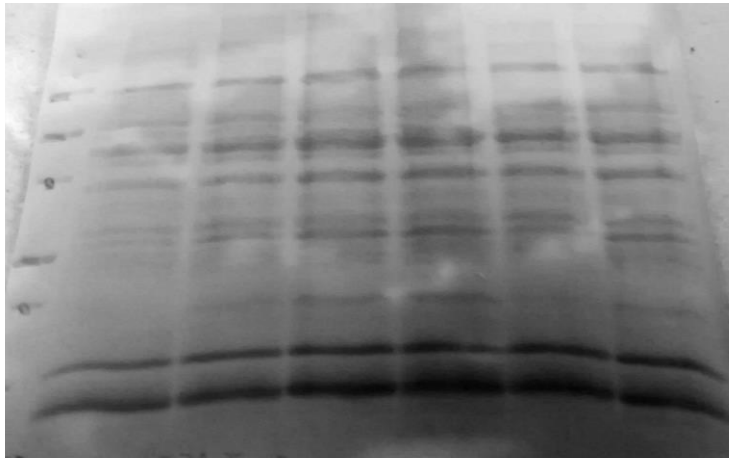

6

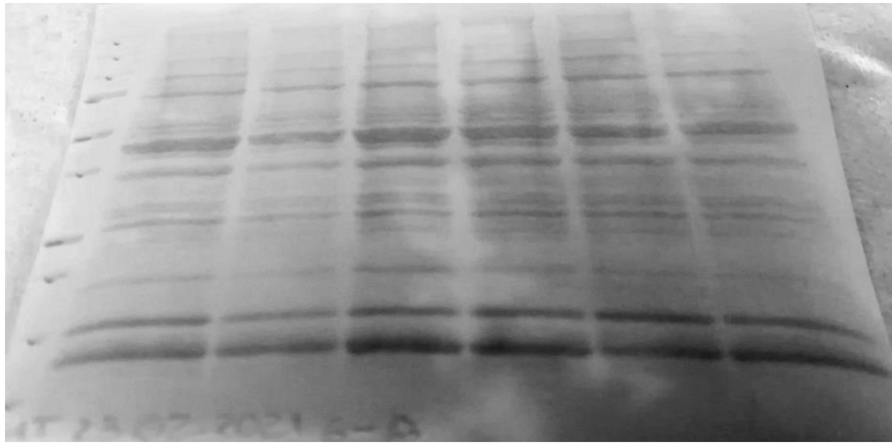

MWM: molecular weight marker

Samples order from the left in each of the 6 membranes: MWM | MTX | DOX18 | **CTRL1 | DOX1 | CTRL2 | DOX2**

Mfn1 - Blot

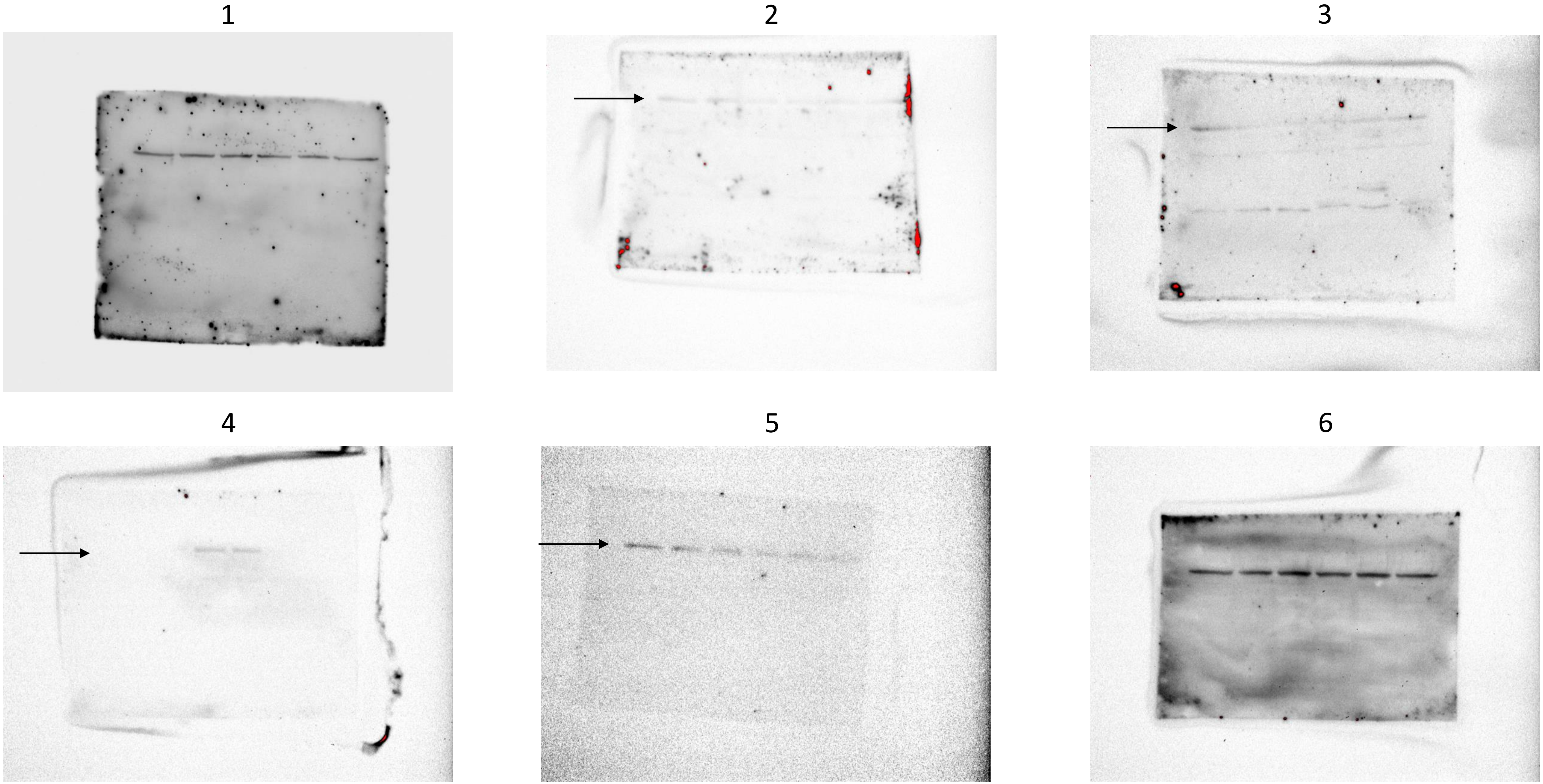

MWM: molecular weight marker

Samples order from the left in each of the 6 membranes: MWM | MTX | DOX18 | **CTRL1 | DOX1 | CTRL2 | DOX2**

Beclin1 - PonceauS

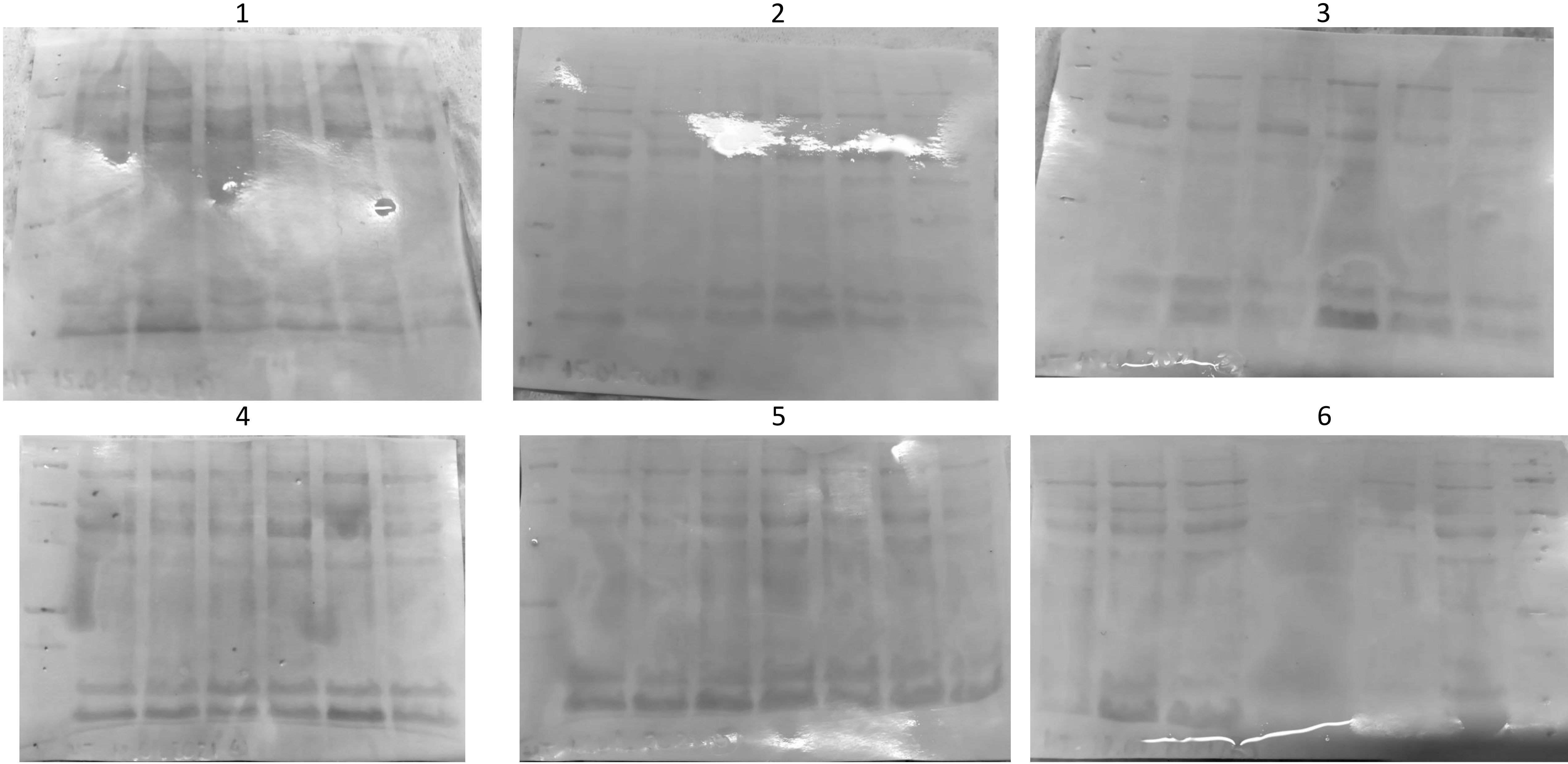

MWM: molecular weight marker

Samples order from the left in each of the 6 membranes: MWM | MTX | DOX18 | **CTRL1 | DOX1 | CTRL2 | DOX2**

Beclin1 - Blot

1

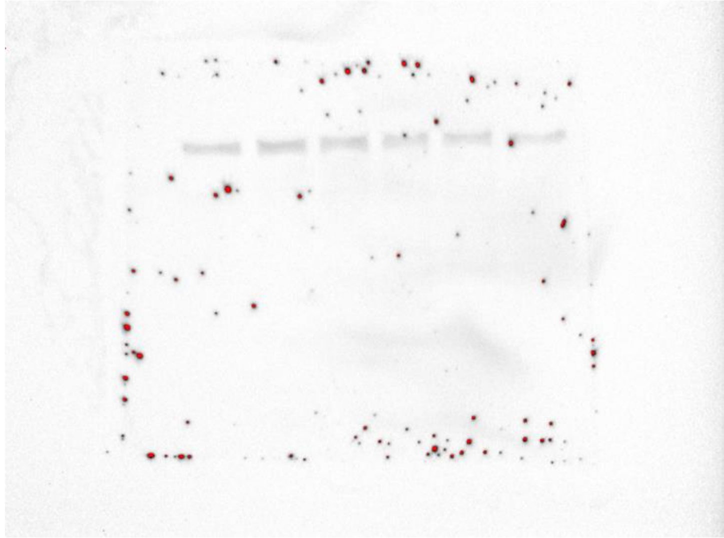

2

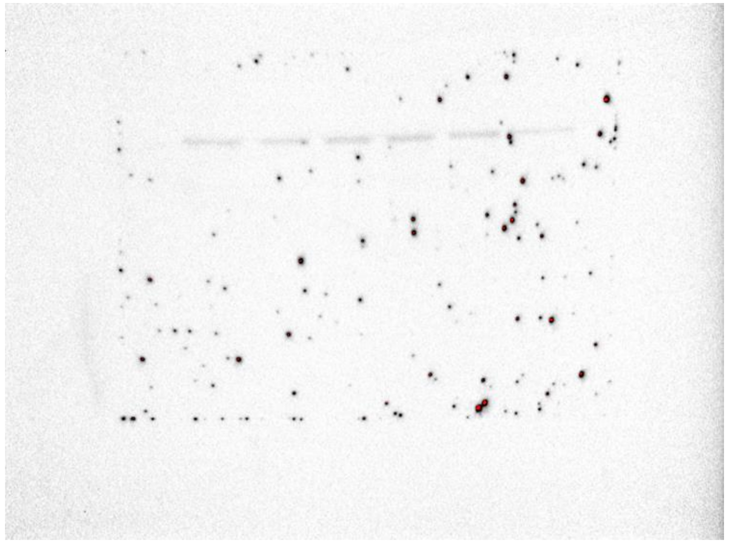

3

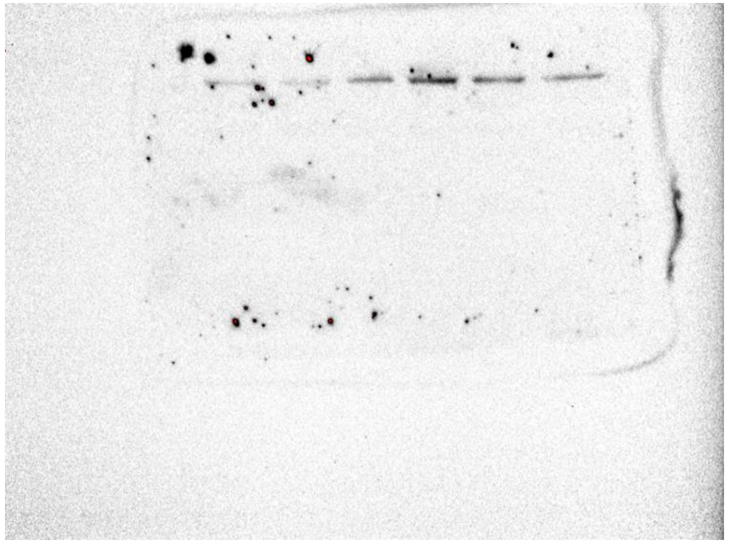

4

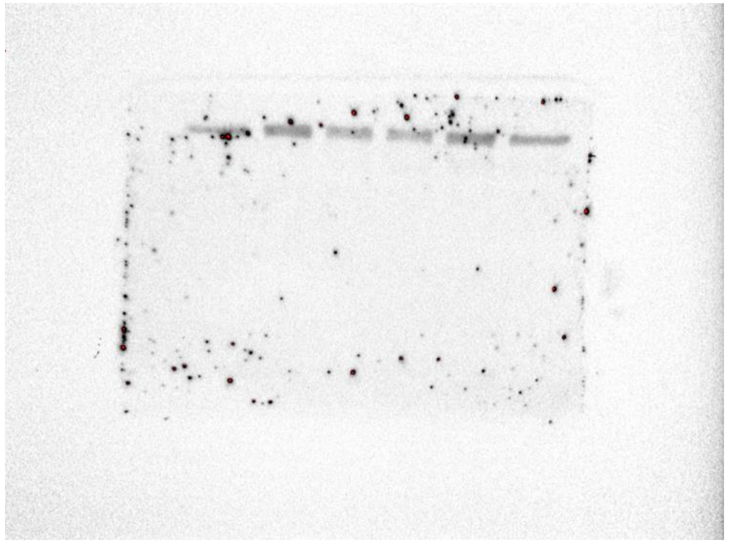

5

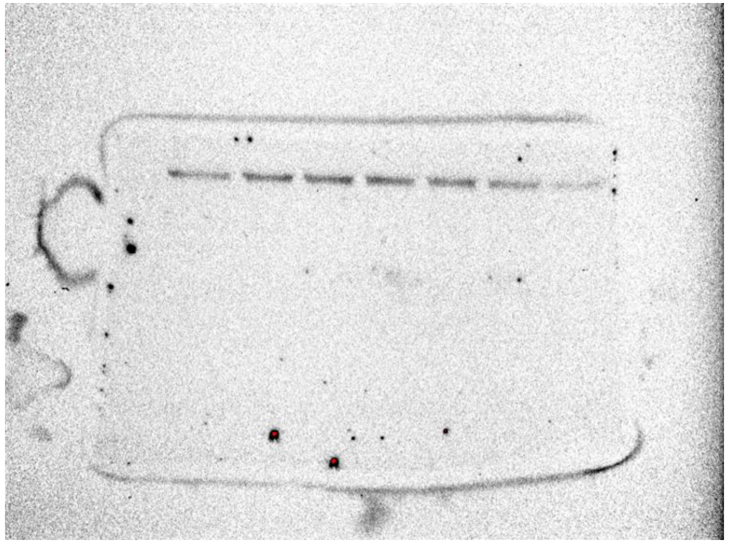

6

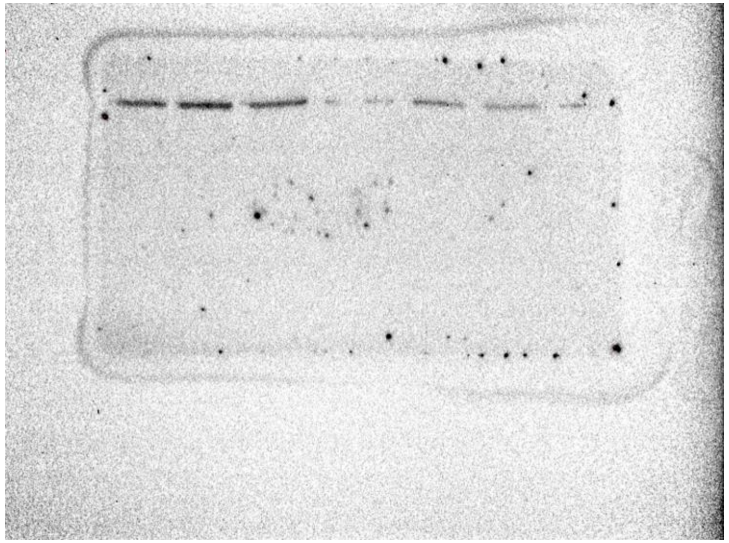

MWM: molecular weight marker

Samples order from the left in each of the 6 membranes: MWM | MTX | DOX18 | **CTRL1 | DOX1 | CTRL2 | DOX2**

ATG5 - PonceauS

1

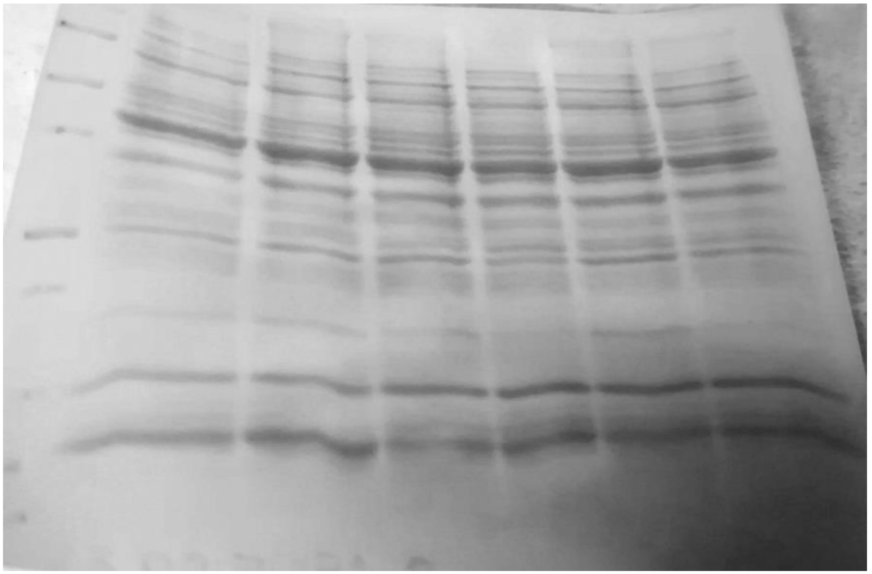

2

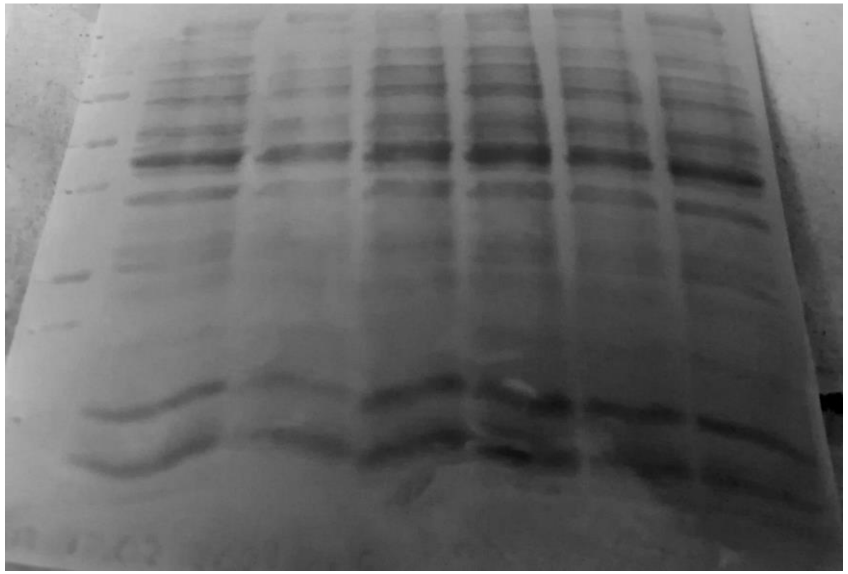

3

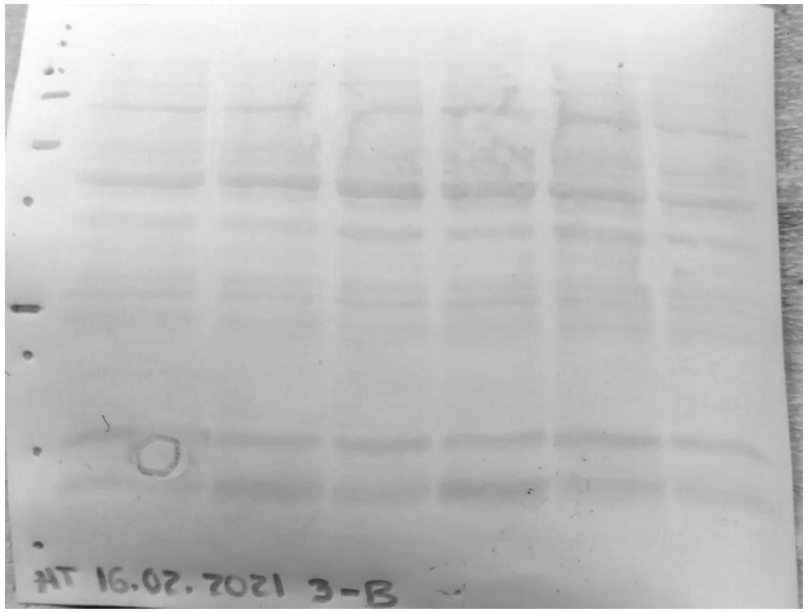

4

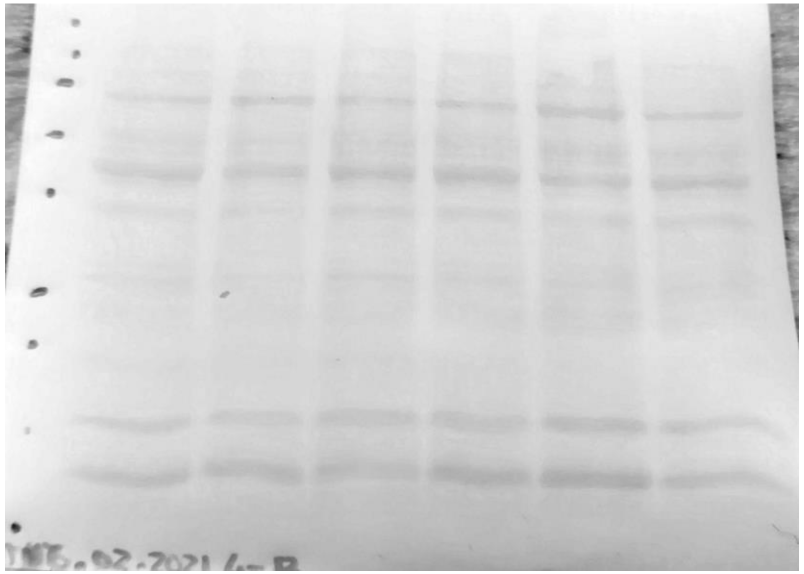

5

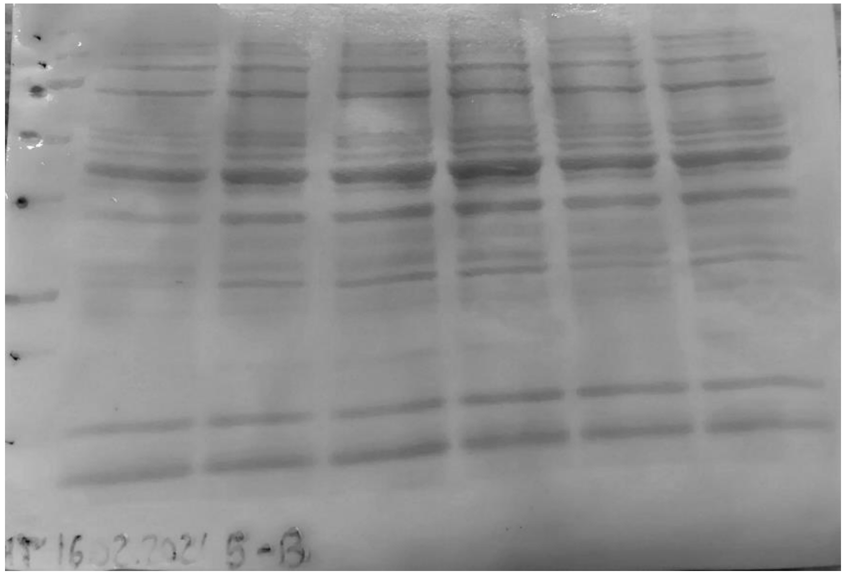

6

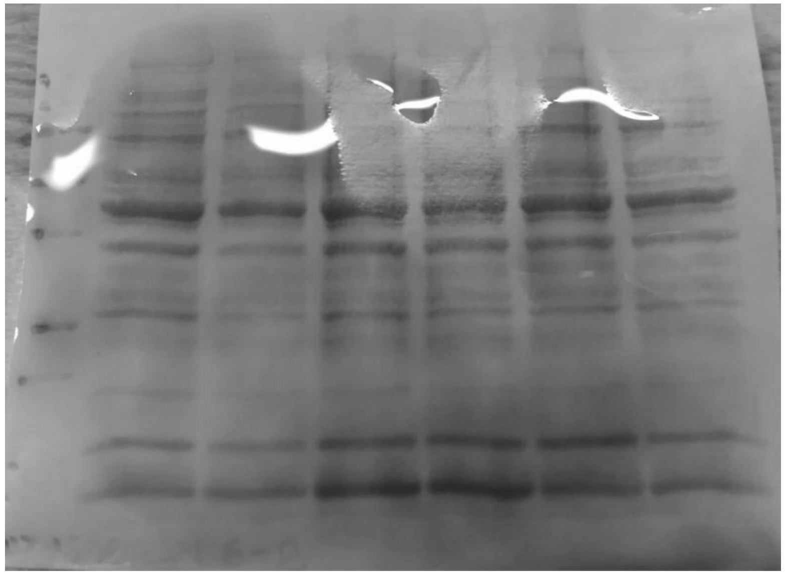

MWM: molecular weight marker

Samples order from the left in each of the 6 membranes: MWM | MTX | DOX18 | **CTRL1 | DOX1 | CTRL2 | DOX2**

ATG5 - Blot

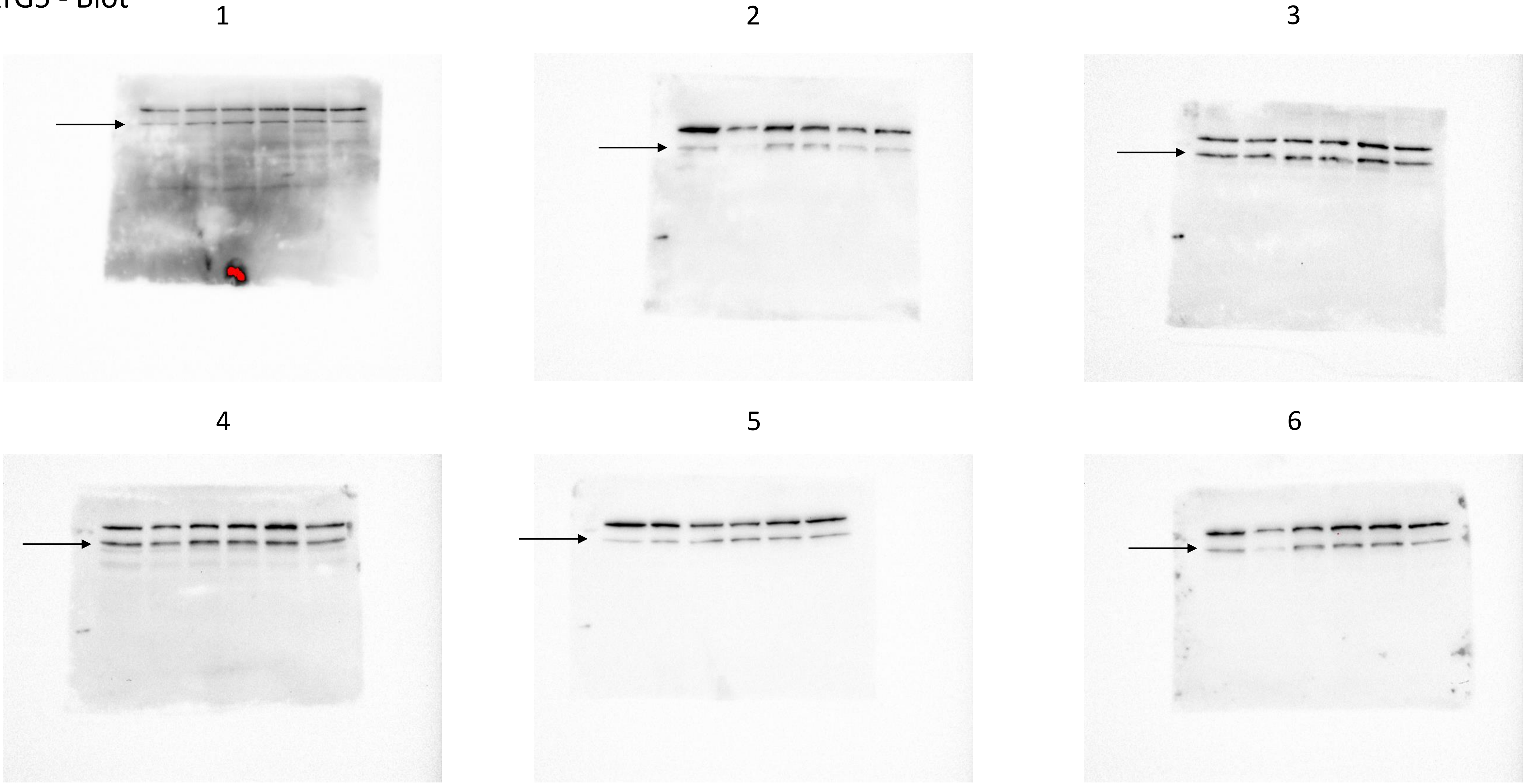

MWM: molecular weight marker

Samples order from the left in each of the 6 membranes: MWM | MTX | DOX18 | **CTRL1 | DOX1 | CTRL2 | DOX2**

LC3B - PonceauS

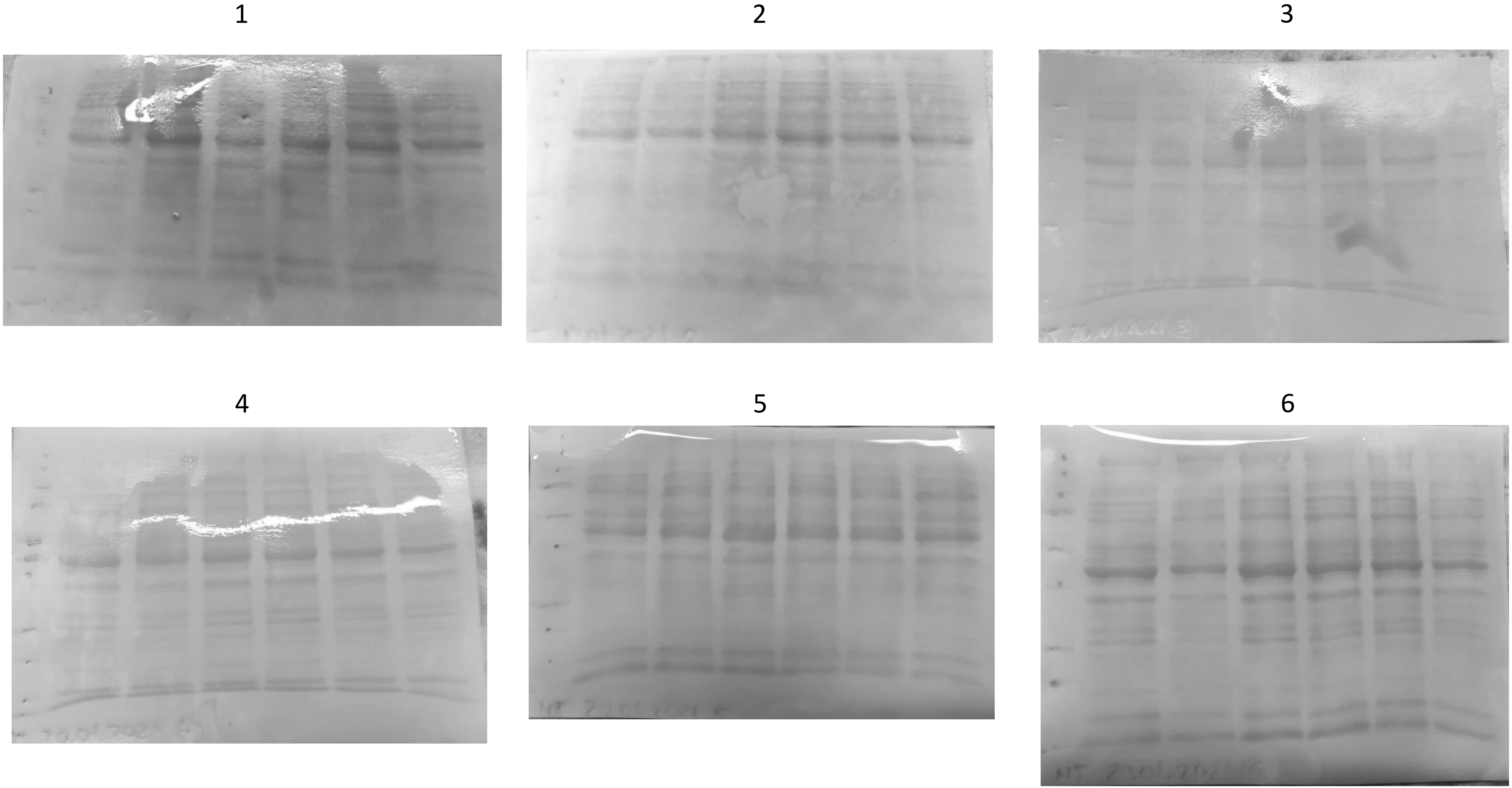

MWM: molecular weight marker

Samples order from the left in each of the 6 membranes: MWM | MTX | DOX18 | **CTRL1 | DOX1 | CTRL2 | DOX2**

LC3B - Blot

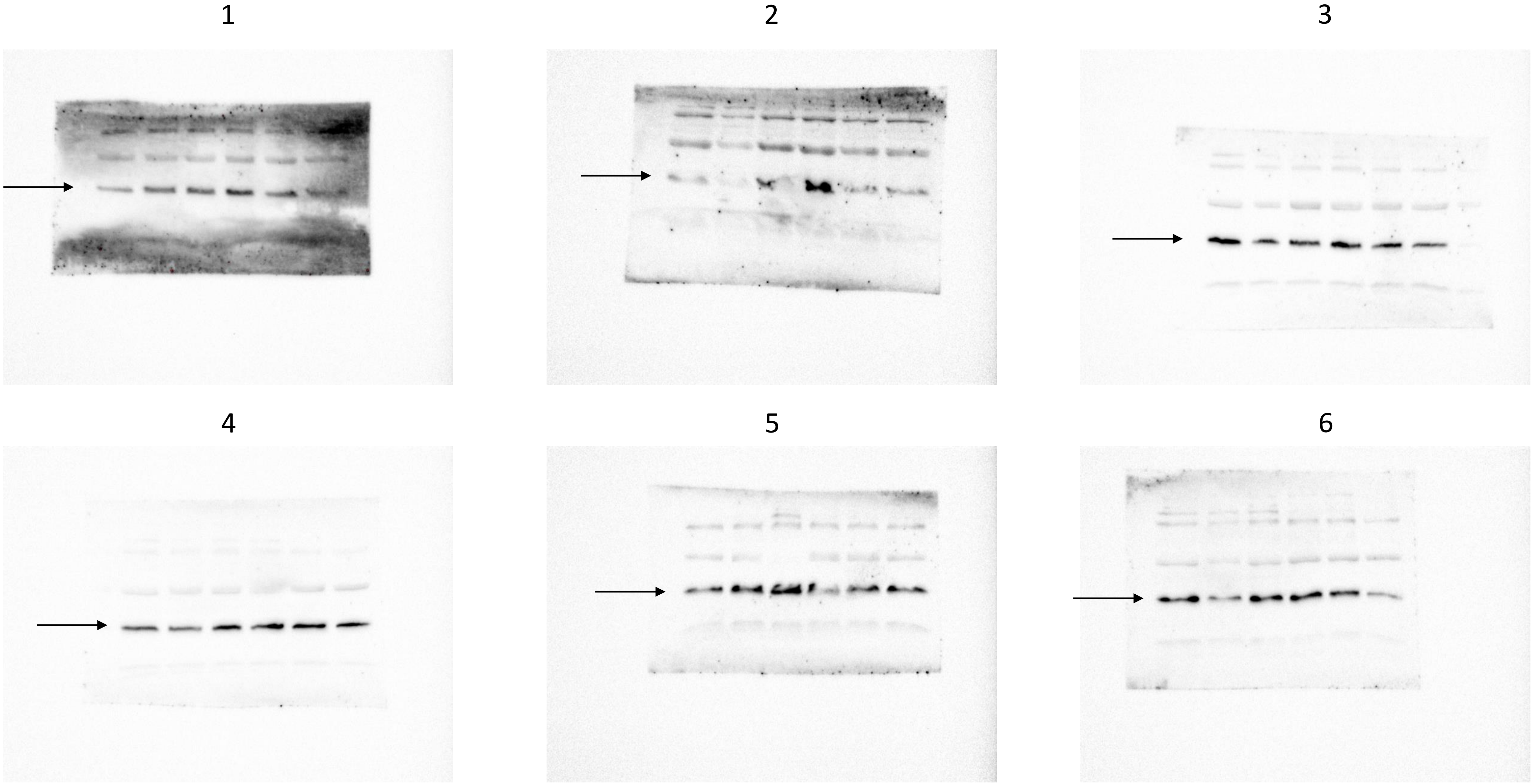

MWM: molecular weight marker

Samples order from the left in each of the 6 membranes: MWM | MTX | DOX18 | **CTRL1 | DOX1 | CTRL2 | DOX2**

Parkin - PonceauS

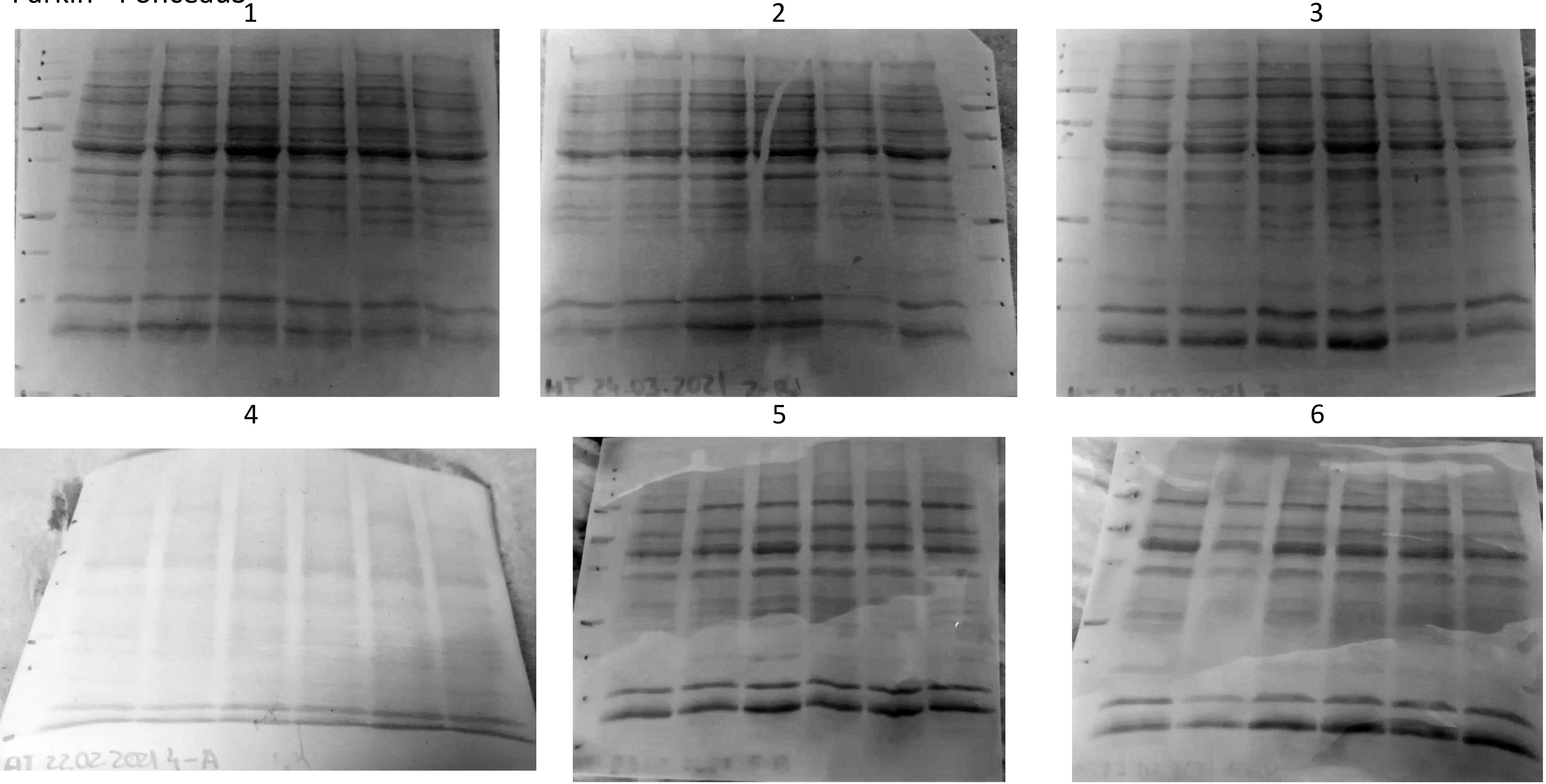

MWM: molecular weight marker

Samples order from the left in each of the 6 membranes: MWM | MTX | DOX18 | **CTRL1 | DOX1 | CTRL2 | DOX2**

Parkin - Blot

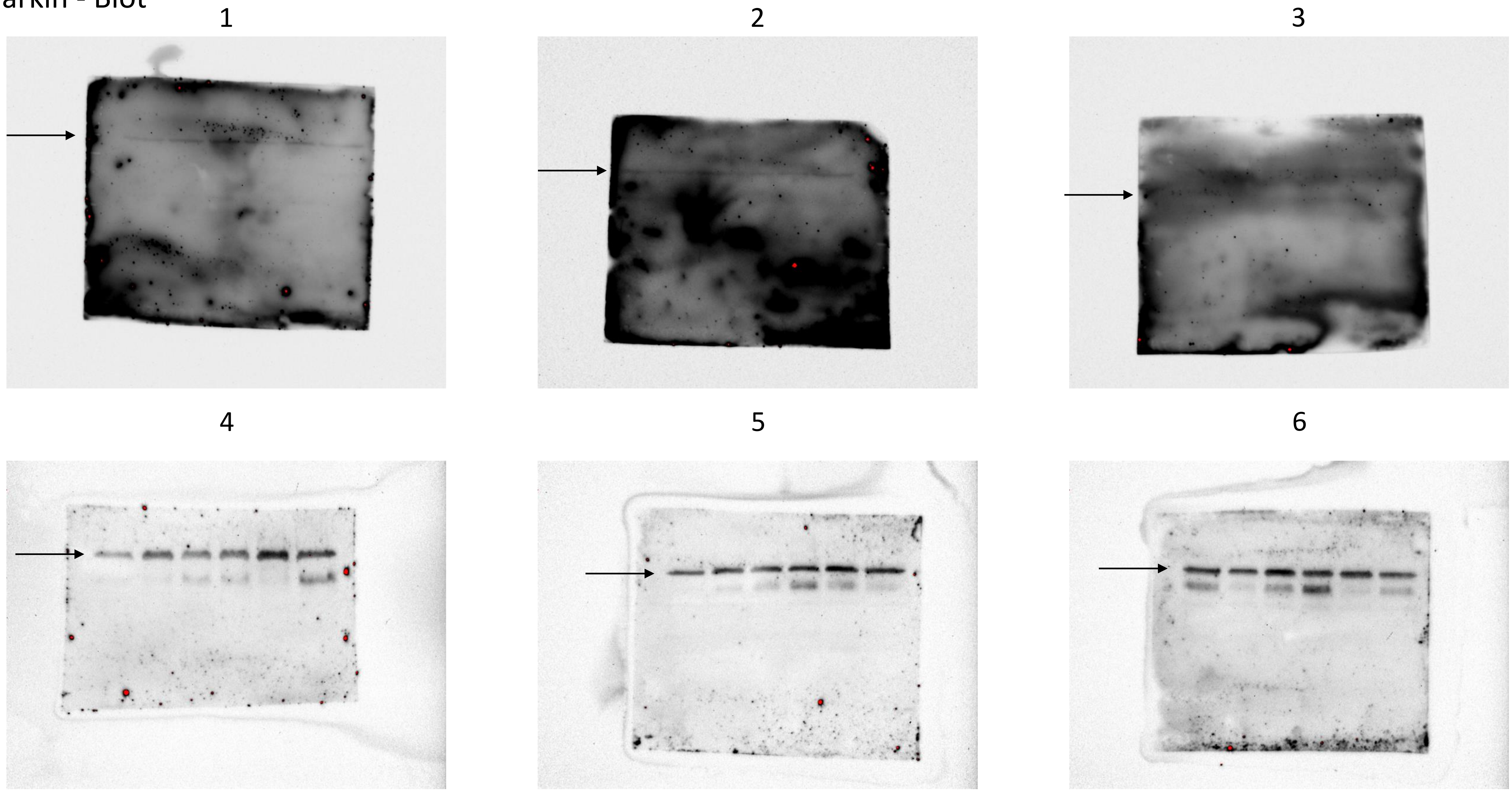

MWM: molecular weight marker

Samples order from the left in each of the 6 membranes: MWM | MTX | DOX18 | **CTRL1 | DOX1 | CTRL2 | DOX2**

BNIP3 - PonceauS

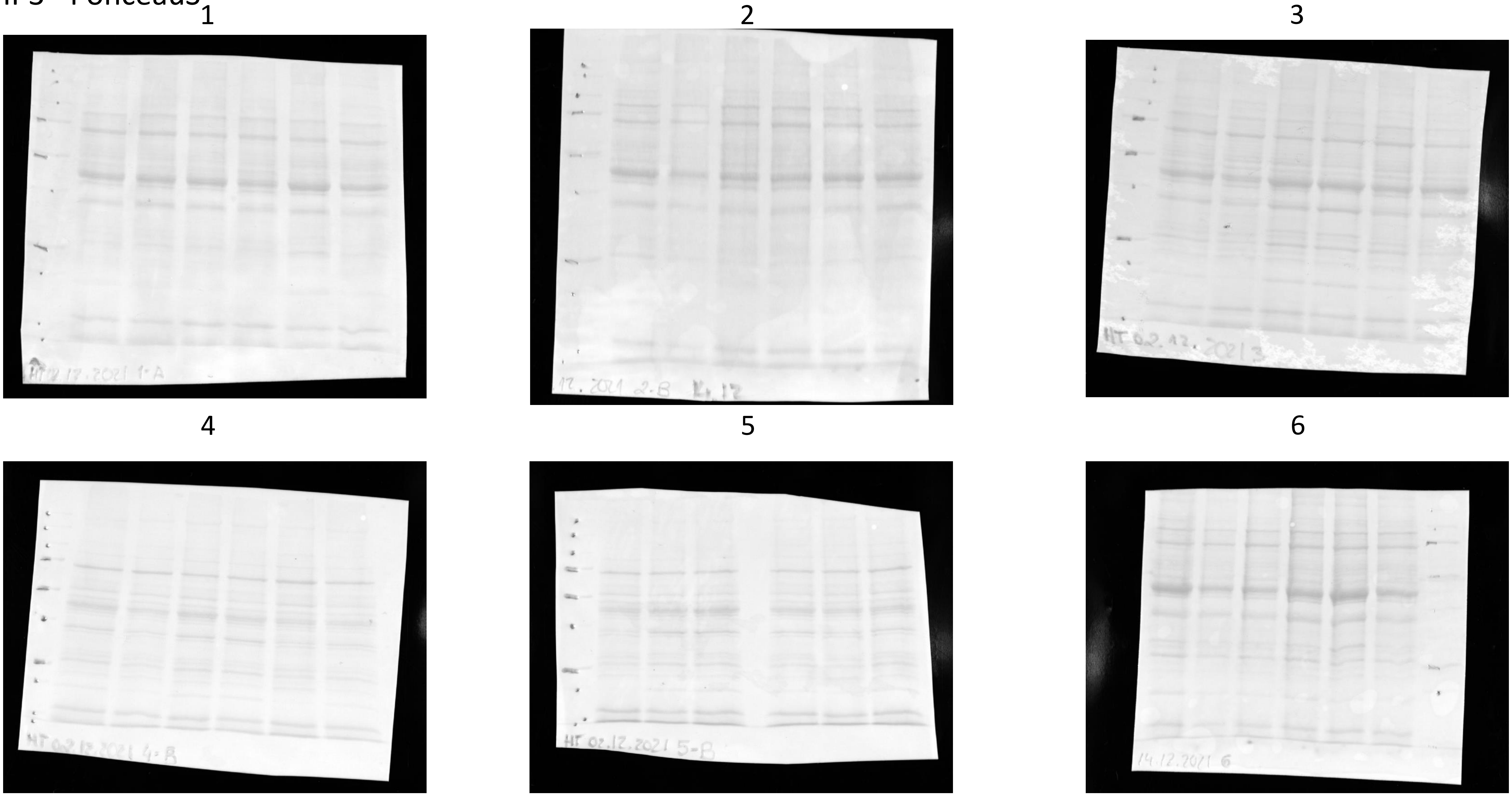

MWM: molecular weight marker

Samples order from the left in each of the 6 membranes: MWM | MTX | DOX18 | **CTRL1 | DOX1 | CTRL2 | DOX2**

BNIP3 - Blot

1

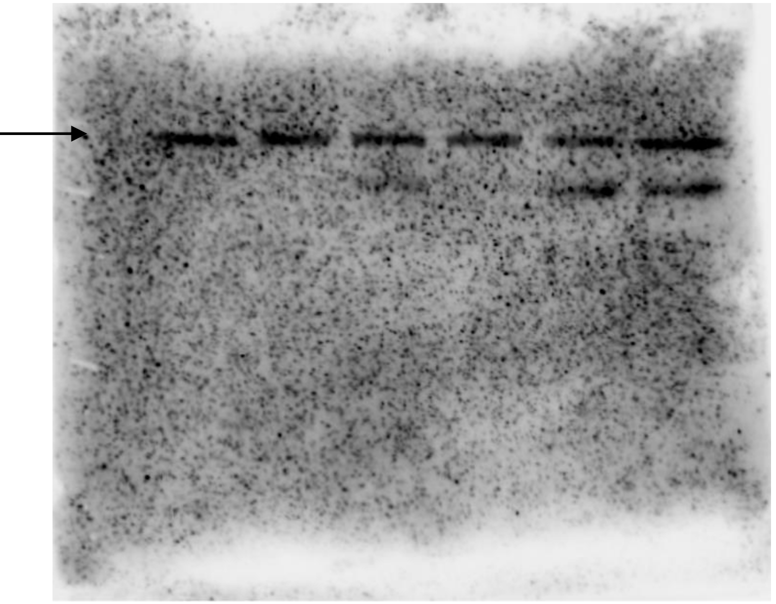

2

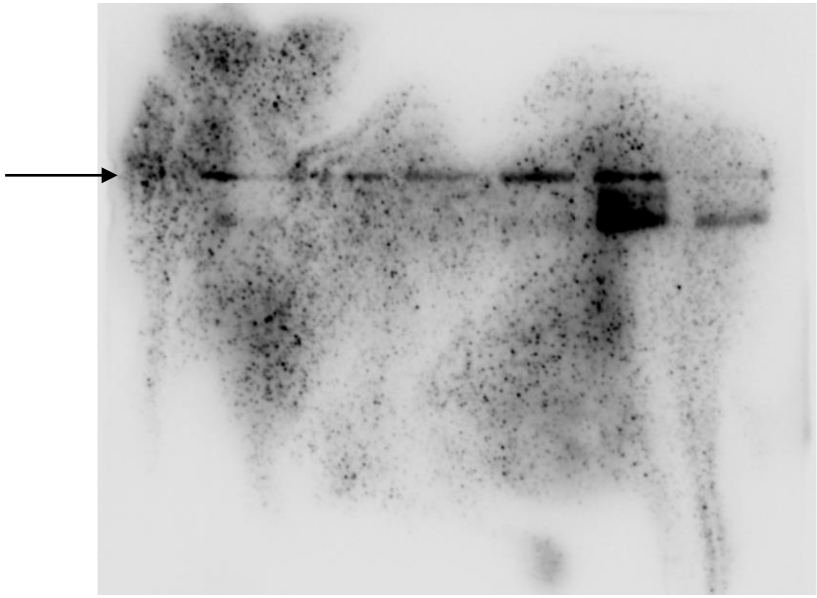

3

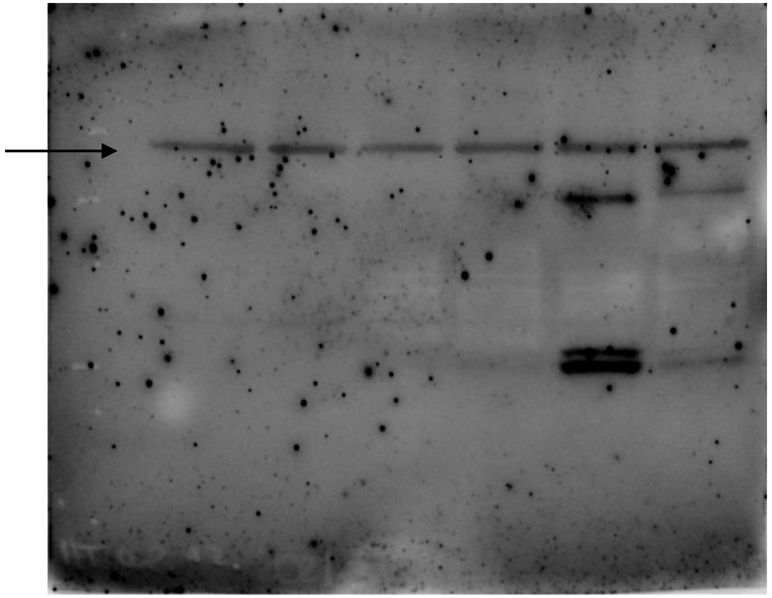

4

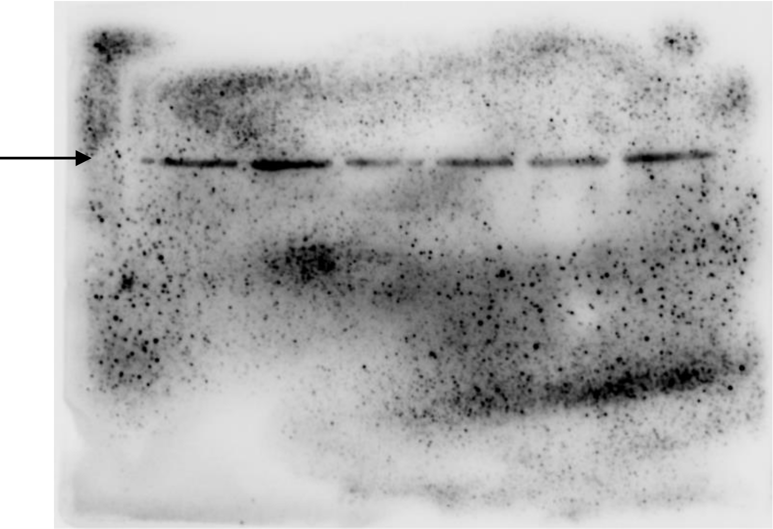

5

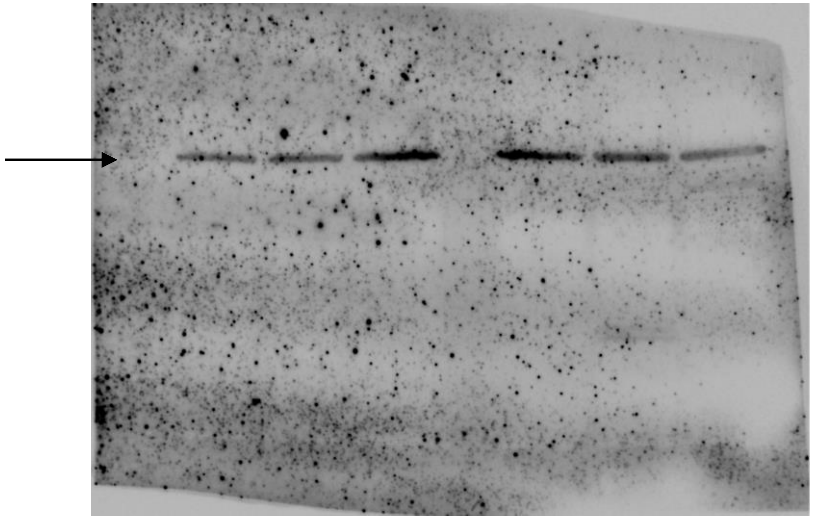

6

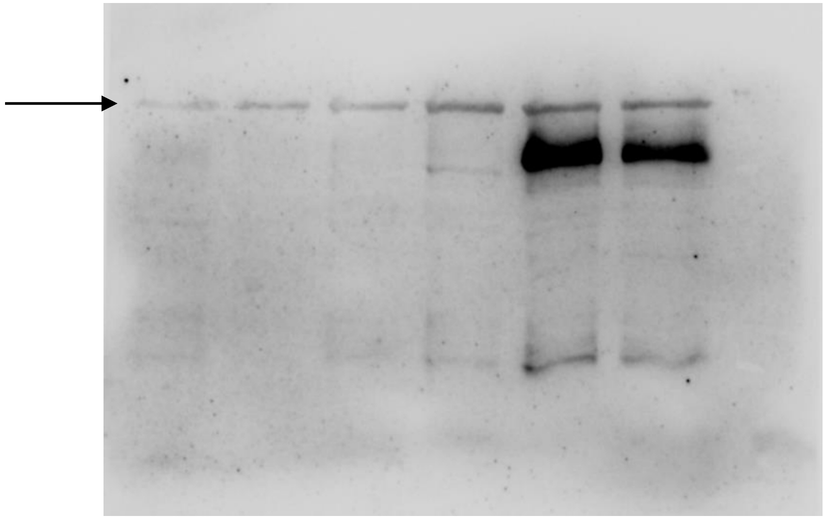

MWM: molecular weight marker

Samples order from the left in each of the 6 membranes: MWM | MTX | DOX18 | **CTRL1 | DOX1 | CTRL2 | DOX2**

BAX - PonceauS

1

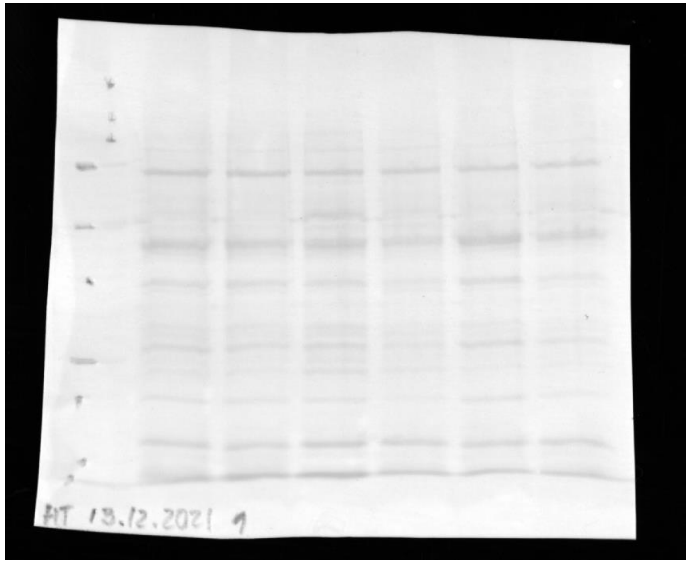

2

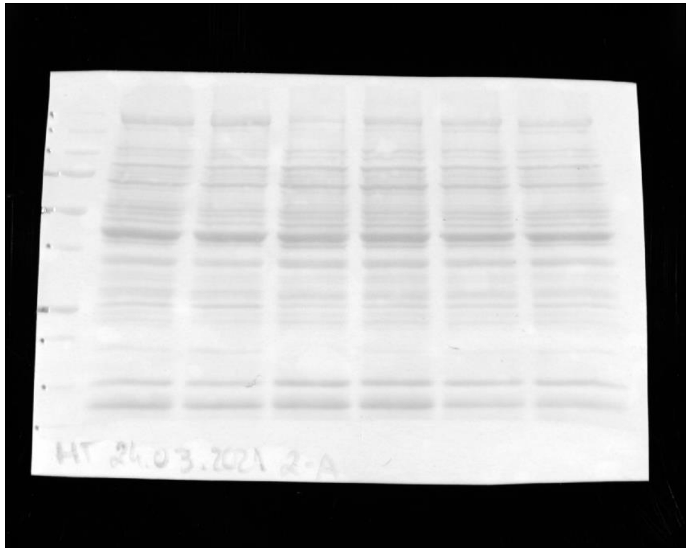

3

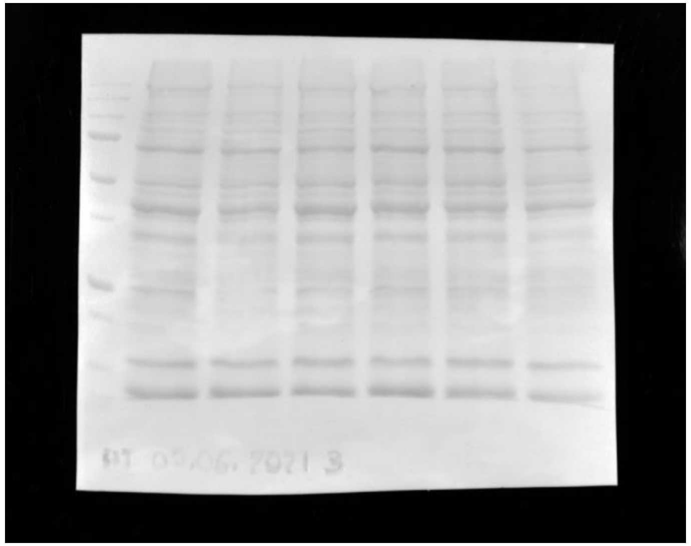

4

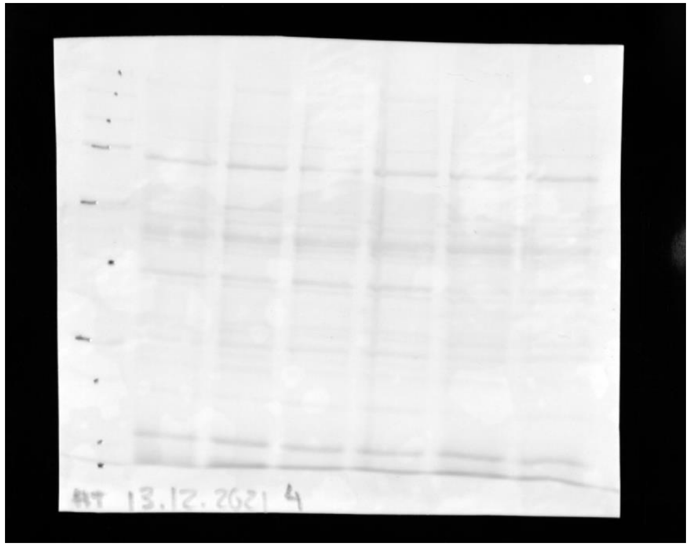

5

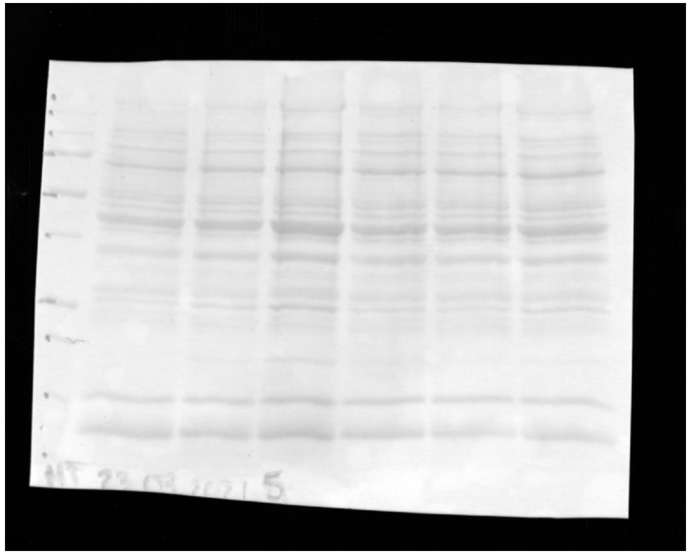

6

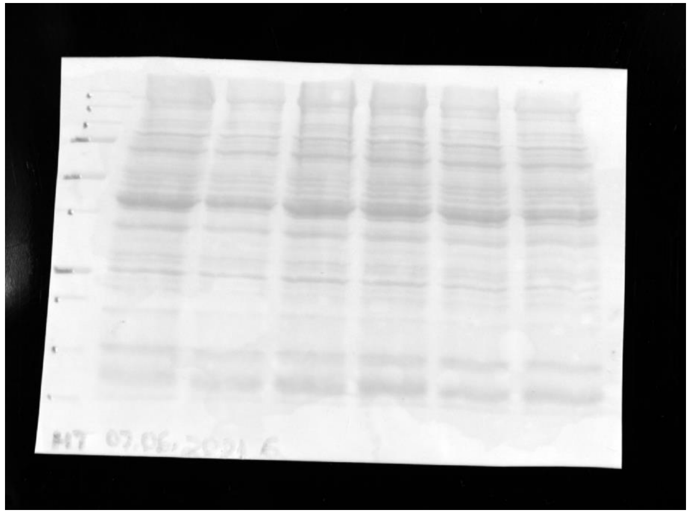

MWM: molecular weight marker

Samples order from the left in each of the 6 membranes: MWM | MTX | DOX18 | **CTRL1 | DOX1 | CTRL2 | DOX2**

BAX - Blot

1

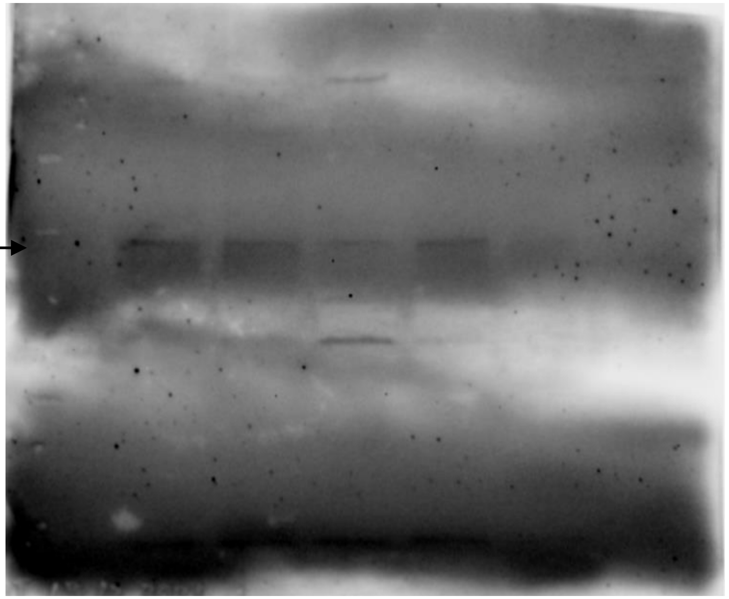

2

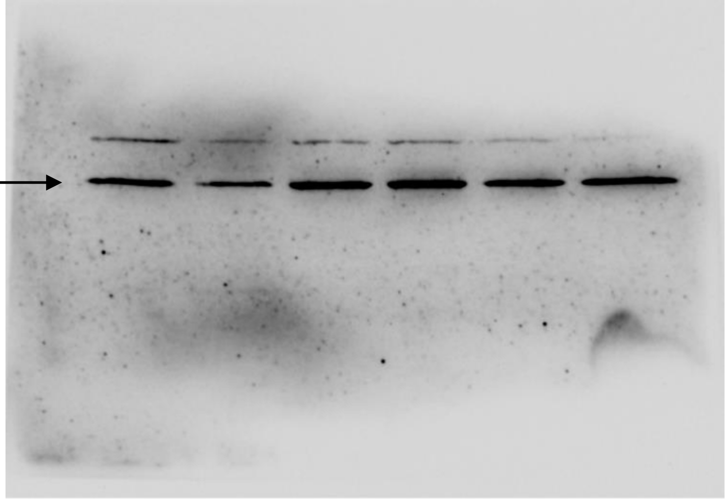

3

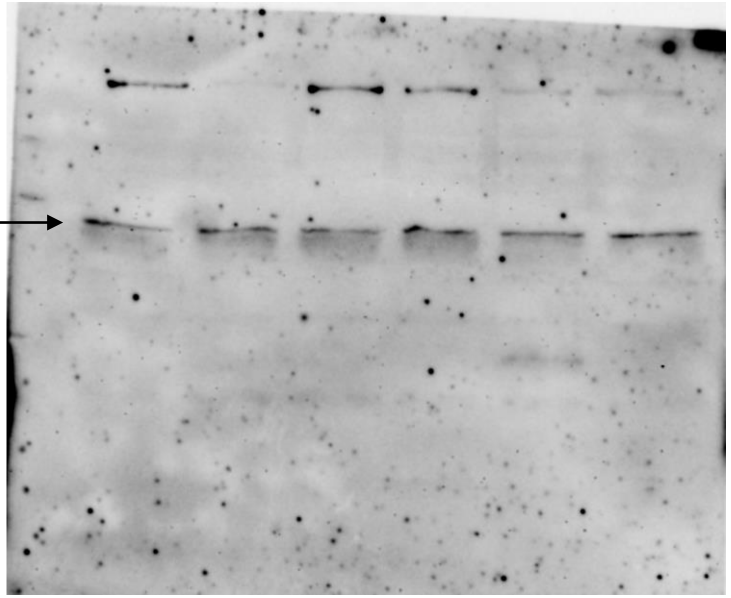

4

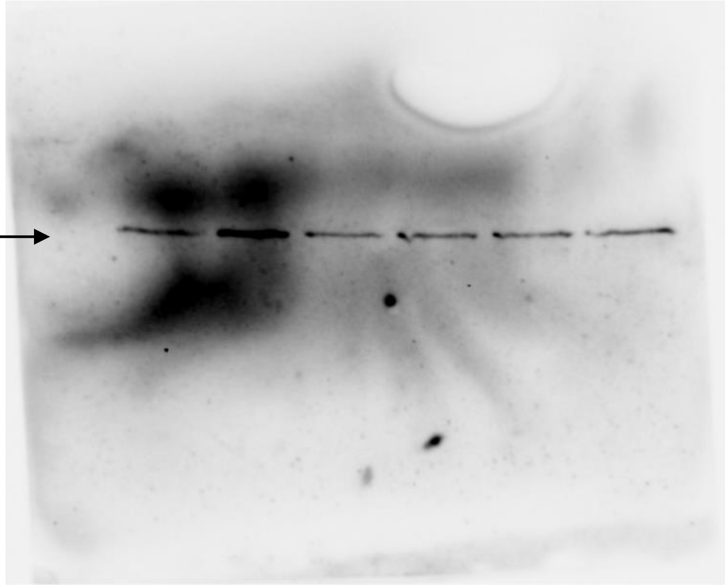

5

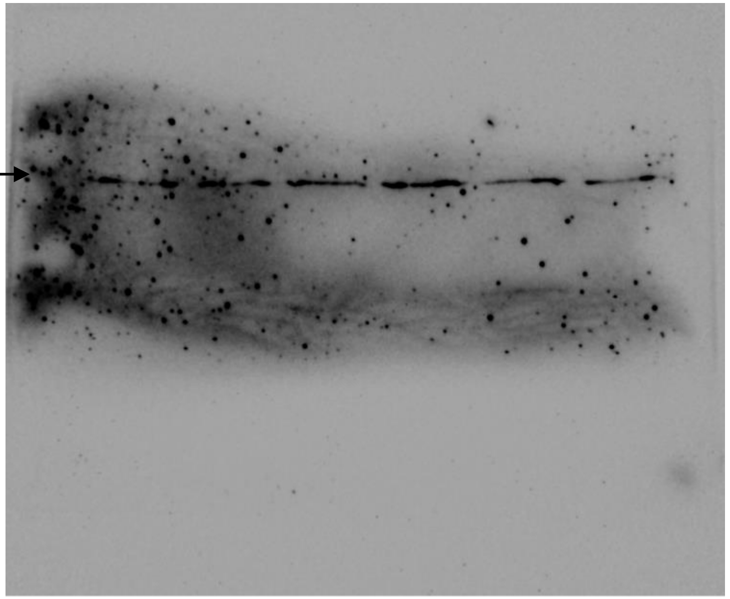

6

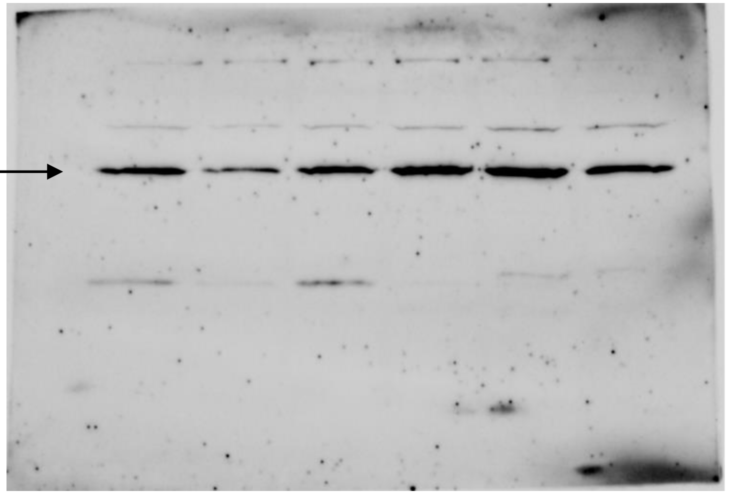

MWM: molecular weight marker

Samples order from the left in each of the 6 membranes: MWM | MTX | DOX18 | **CTRL1 | DOX1 | CTRL2 | DOX2**

BCL2 - PonceauS

1

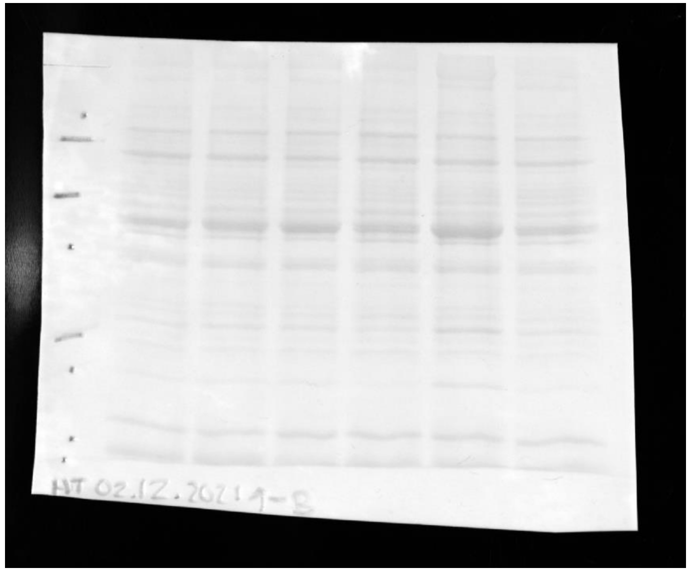

2

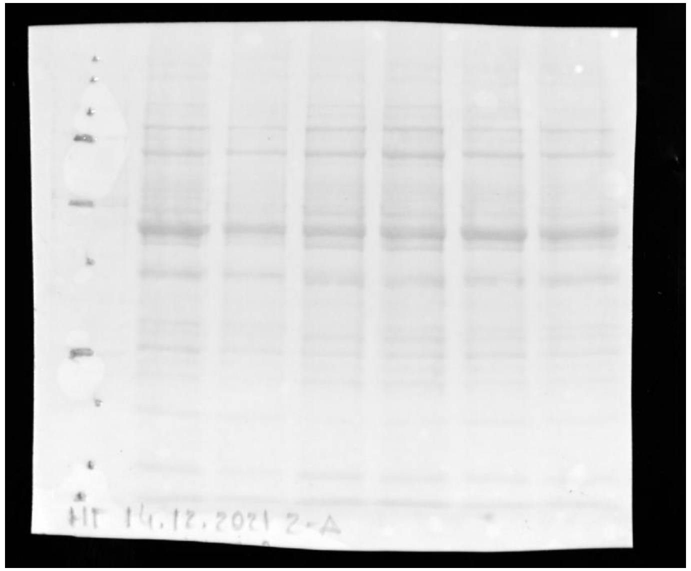

3

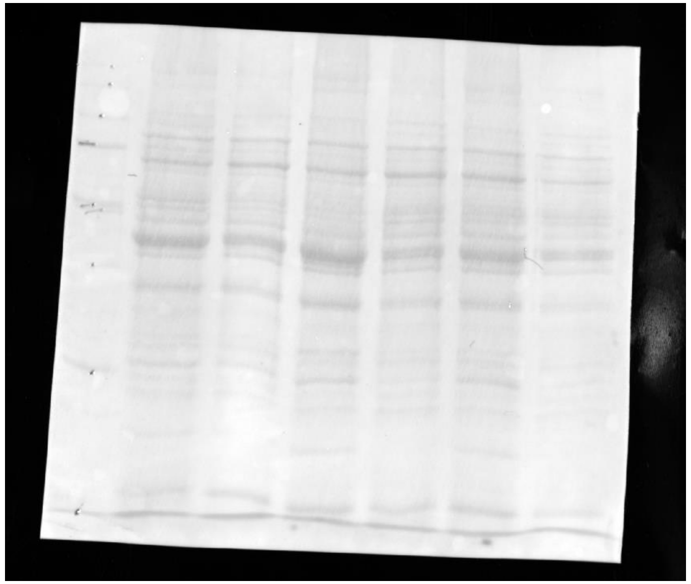

4

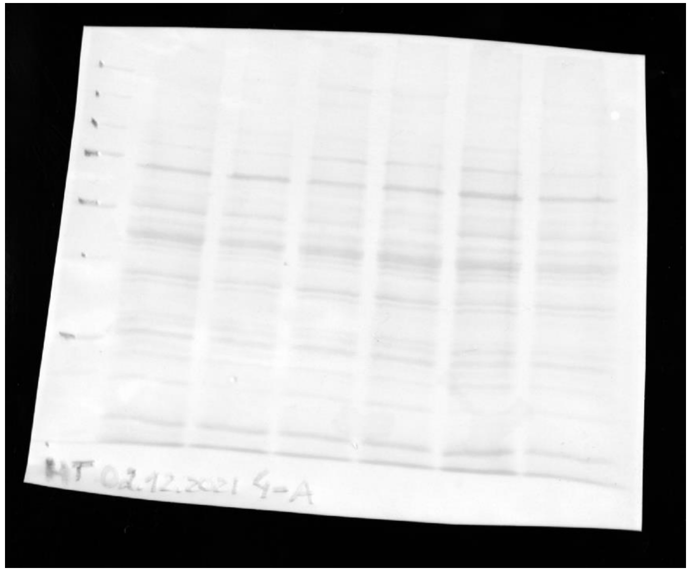

5

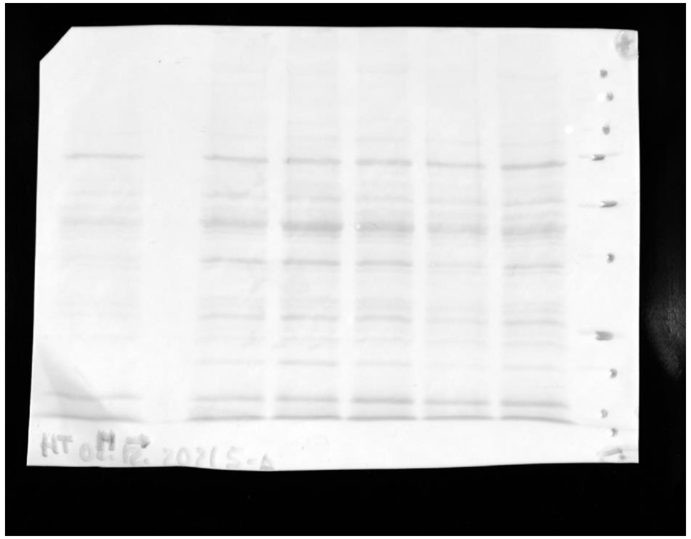

6

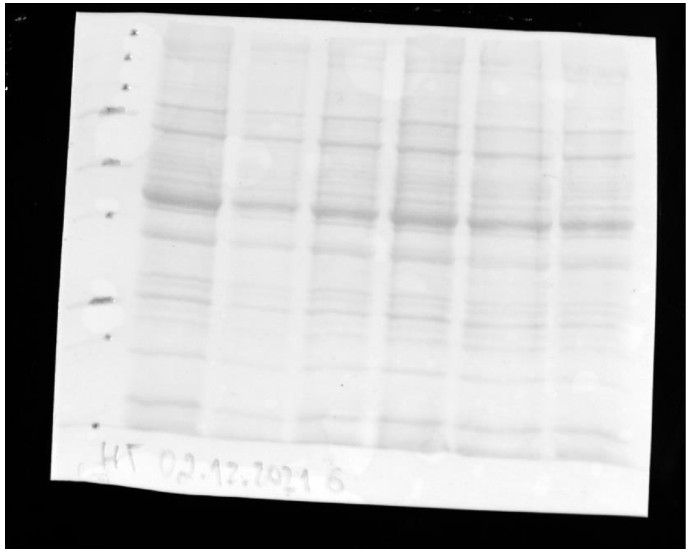

MWM: molecular weight marker

Samples order from the left in each of the 6 membranes: MWM | MTX | DOX18 | **CTRL1 | DOX1 | CTRL2 | DOX2**

BCL2 - Blot

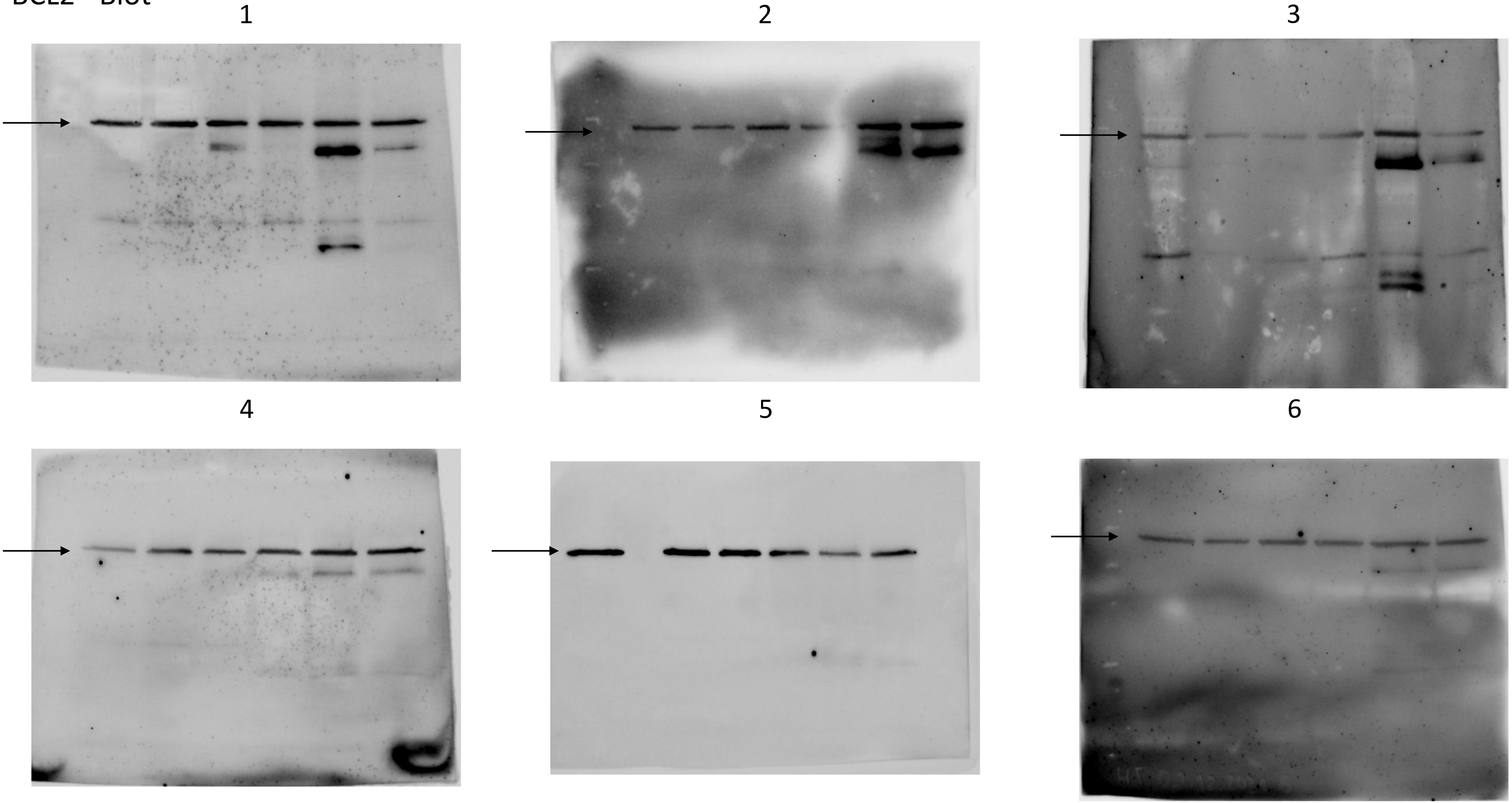

MWM: molecular weight marker

Samples order from the left in each of the 6 membranes: MWM | MTX | DOX18 | **CTRL1 | DOX1 | CTRL2 | DOX2**

HSP27 - PonceauS

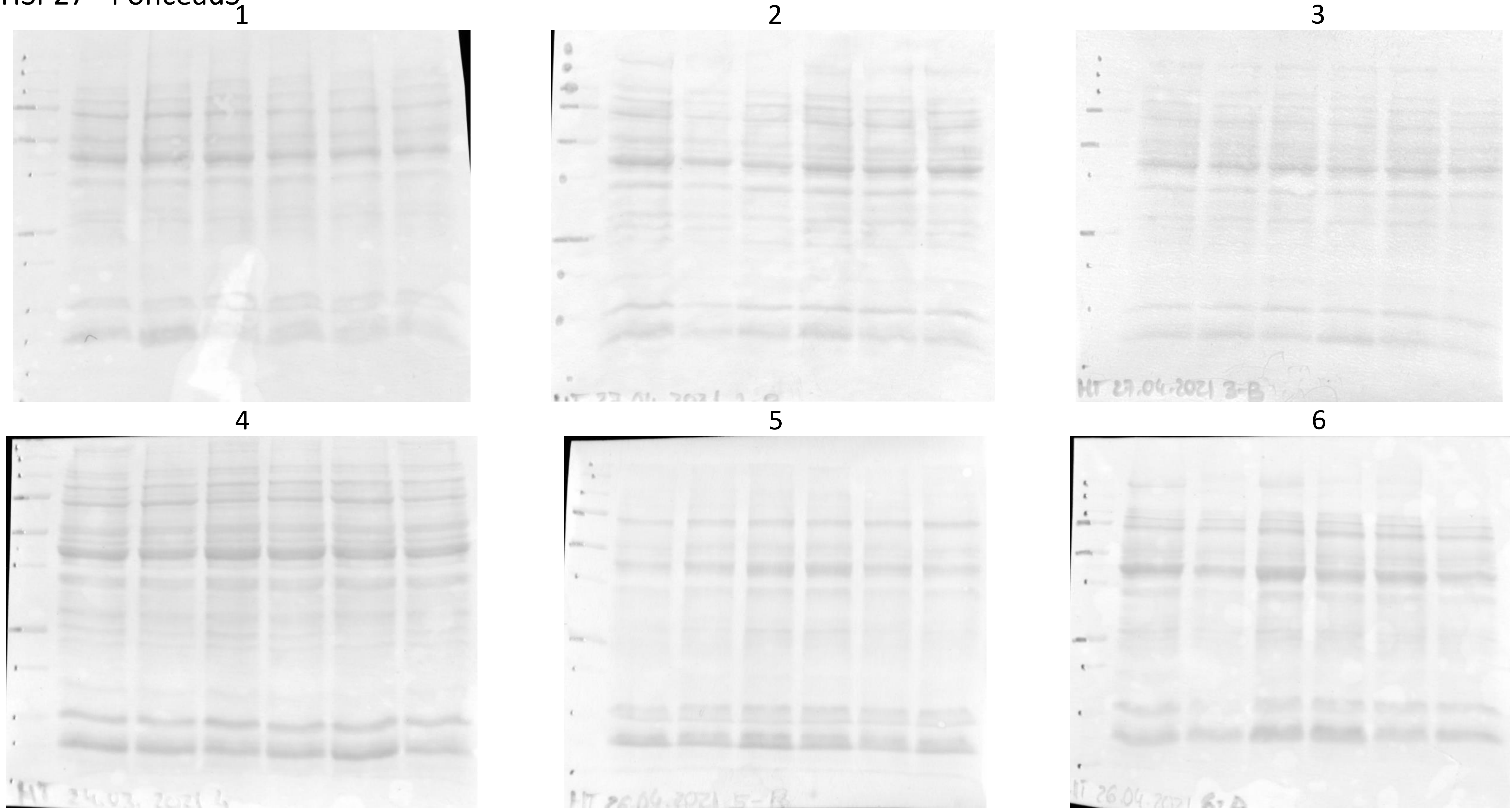

MWM: molecular weight marker

Samples order from the left in each of the 6 membranes: MWM | MTX | DOX18 | **CTRL1 | DOX1 | CTRL2 | DOX2**

HSP27 - Blot

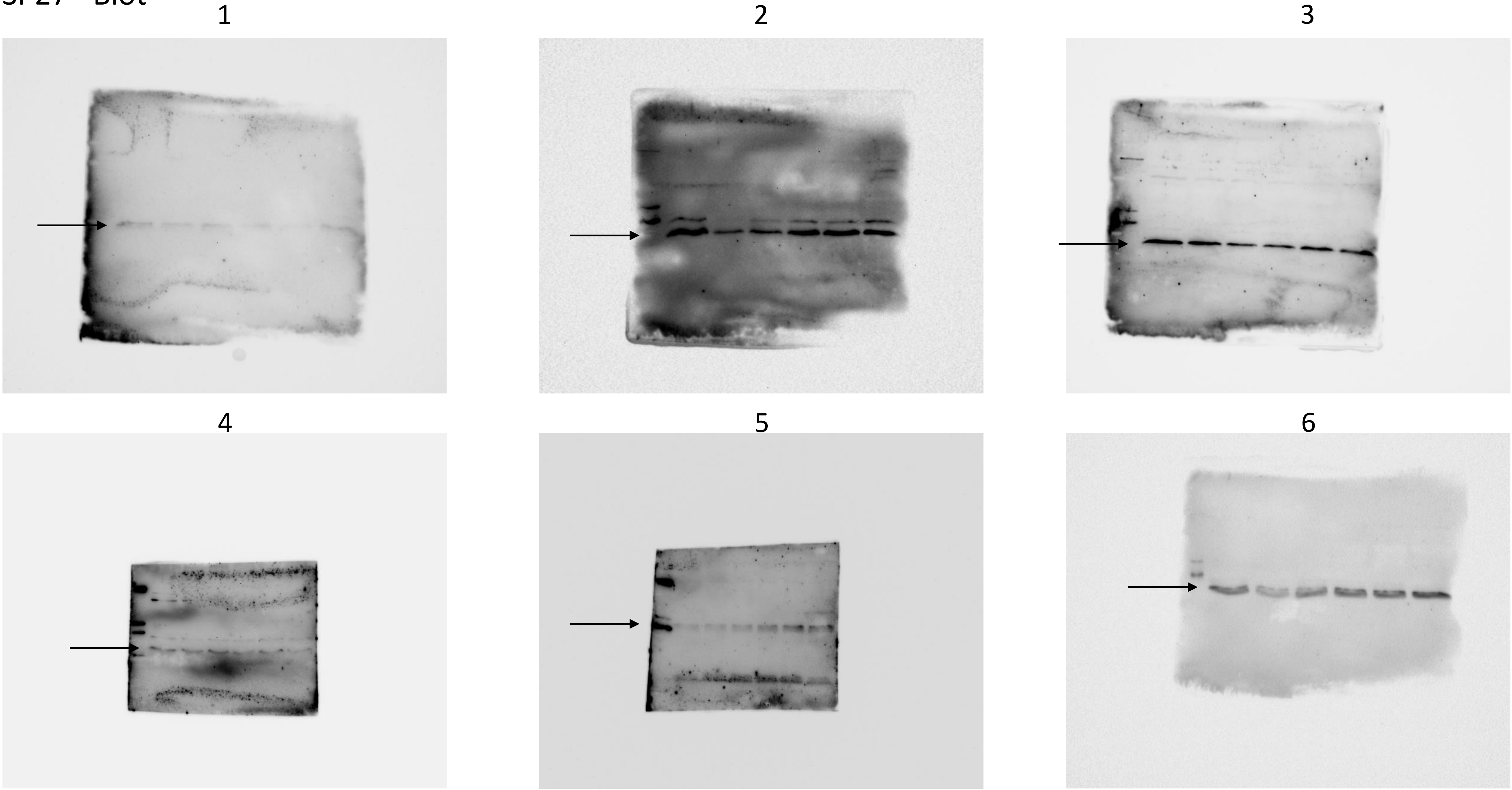

MWM: molecular weight marker

Samples order from the left in each of the 6 membranes: MWM | MTX | DOX18 | **CTRL1 | DOX1 | CTRL2 | DOX2**

HSP70 - PonceauS

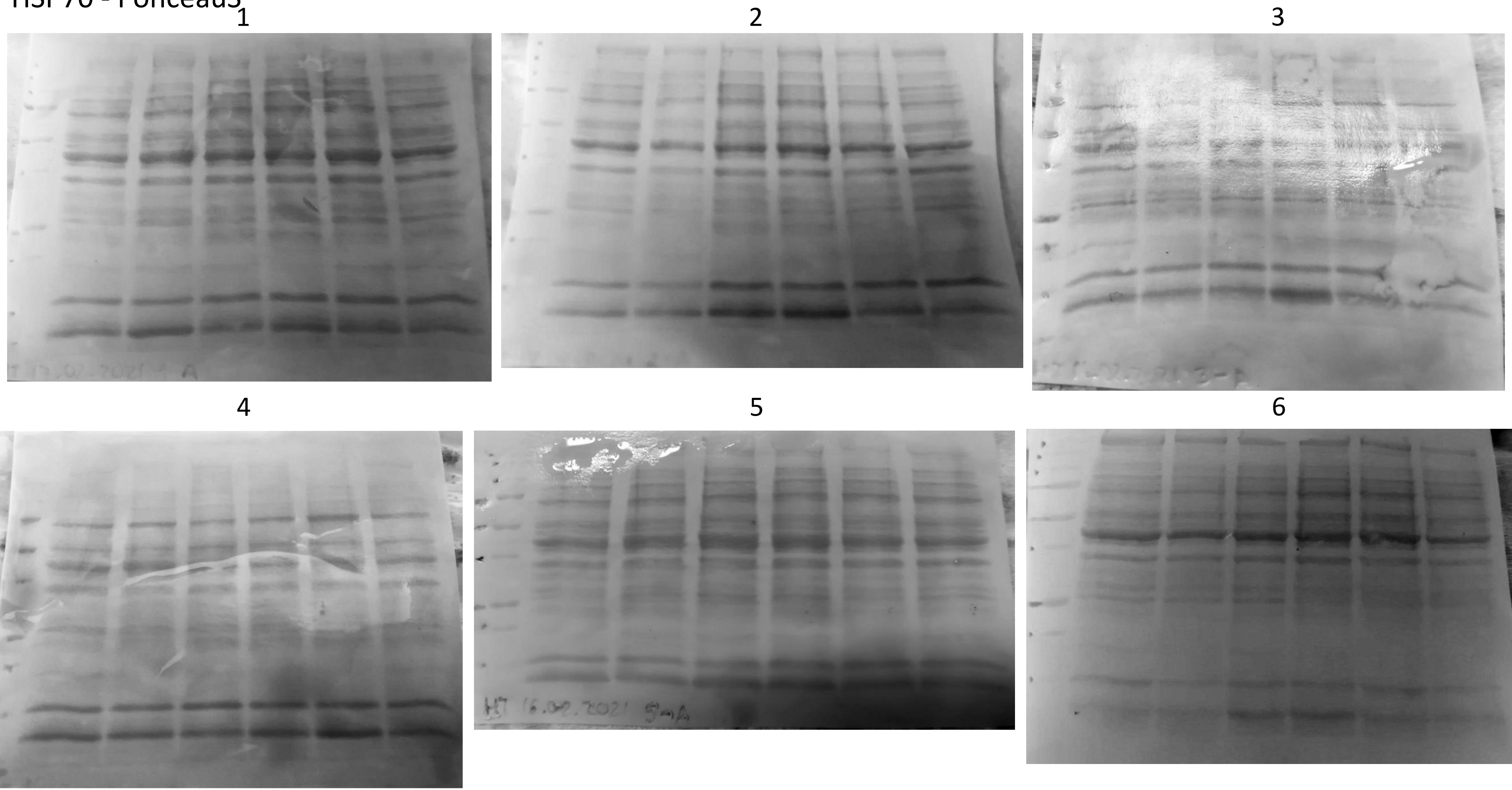

MWM: molecular weight marker

Samples order from the left in each of the 6 membranes: MWM | MTX | DOX18 | **CTRL1 | DOX1 | CTRL2 | DOX2**

HSP70 - Blot

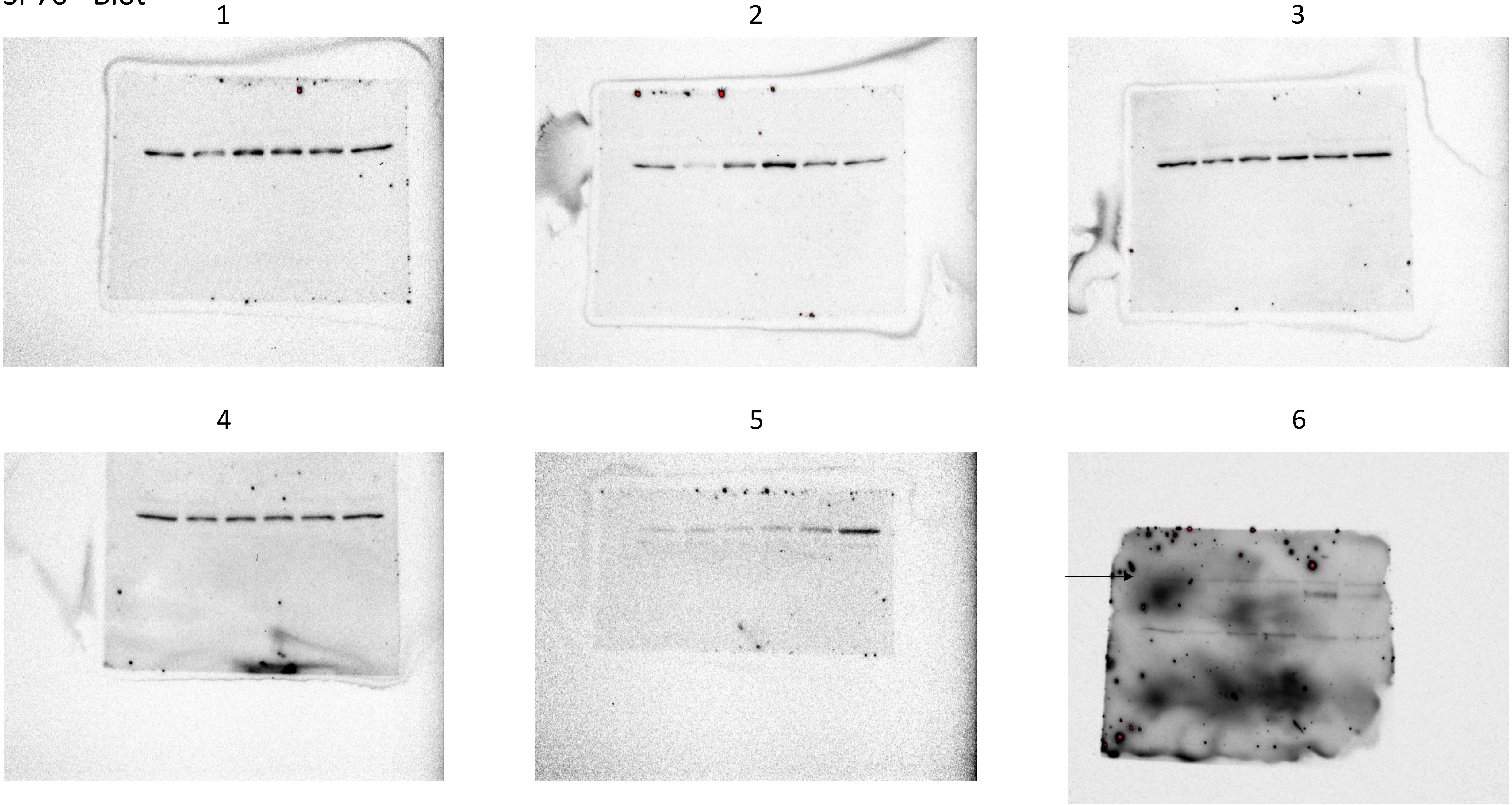

MWM: molecular weight marker

Samples order from the left in each of the 6 membranes: MWM | MTX | DOX18 | **CTRL1 | DOX1 | CTRL2 | DOX2**

SCFR - PonceauS

1

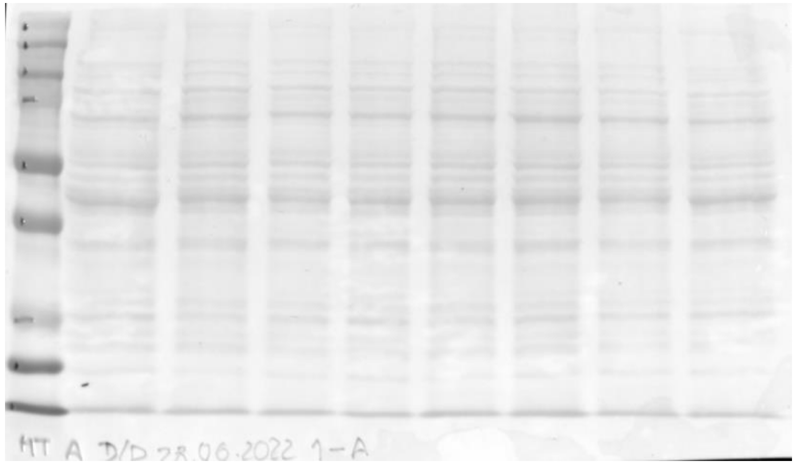

2

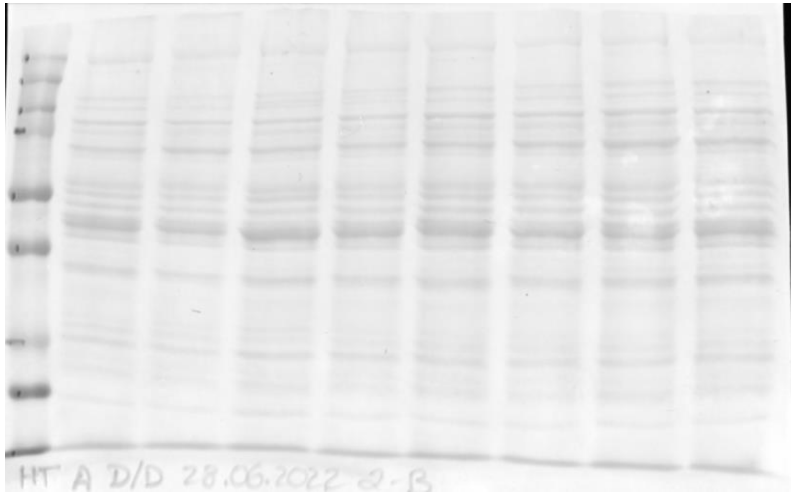

3

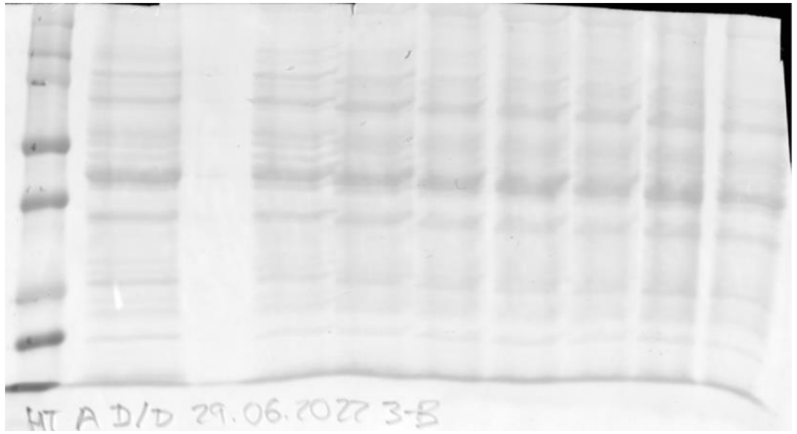

Samples order from the left in each of the 3 membranes: MWM | CTRL1-1 | DOX1-1 | CTRL2-1 | DOX2-1 | CTRL1-2 | DOX1-2 | CTRL2-2 | DOX2-2

MWM: molecular weight marker

SCFR - Blot

1

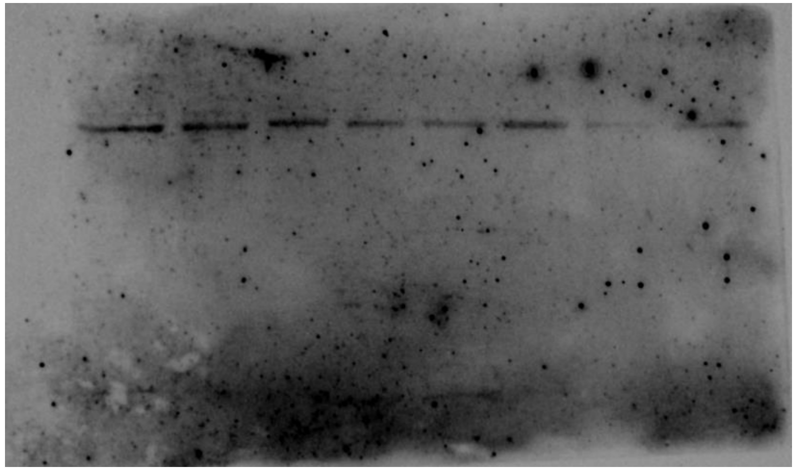

2

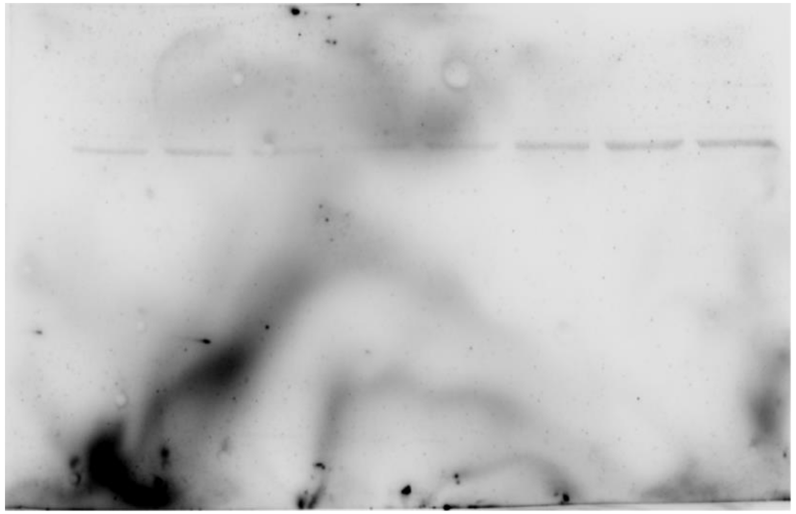

3

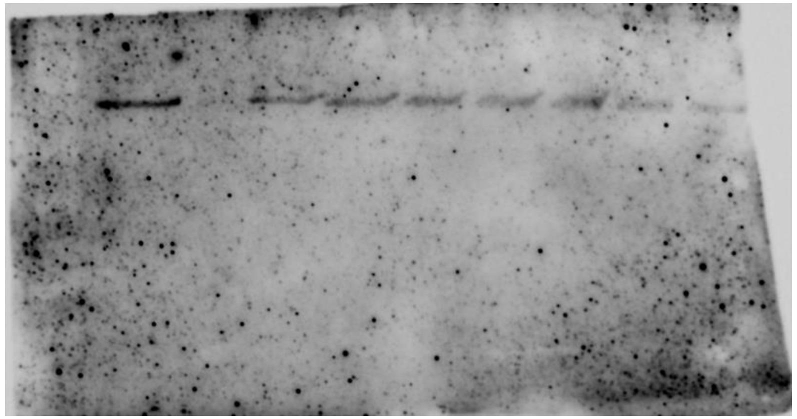

C/EBPβ - PonceauS

1

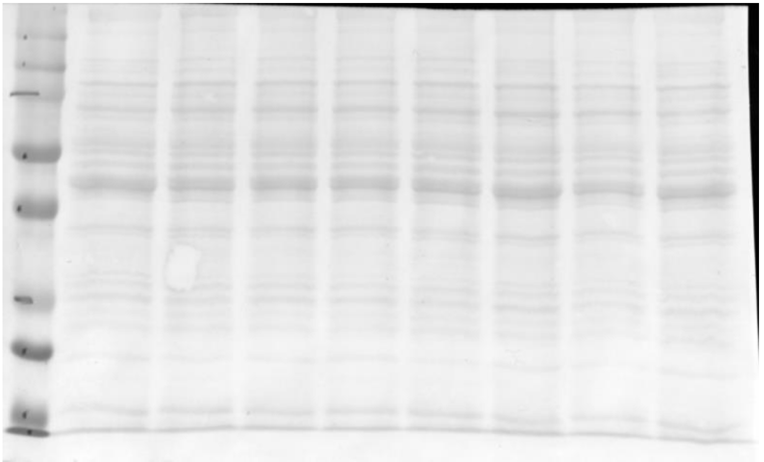

2

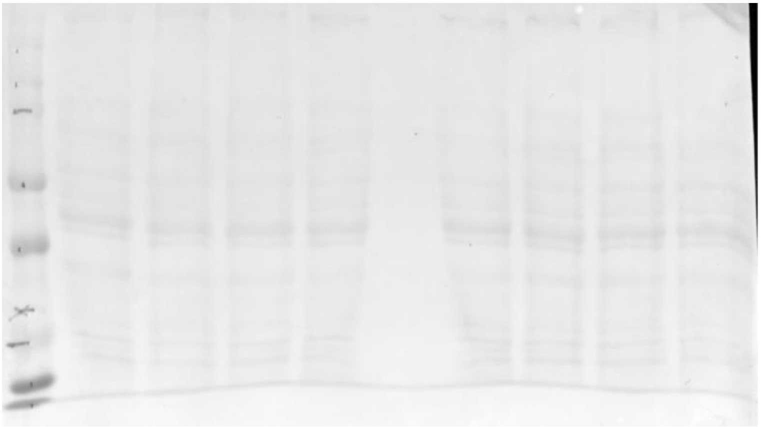

3

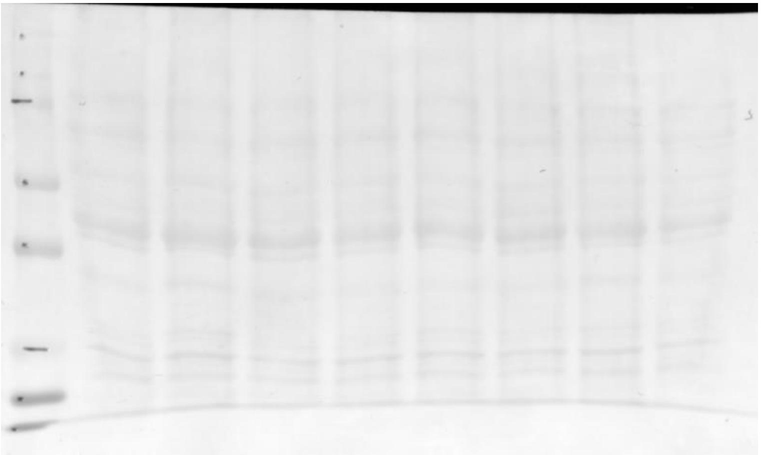

Samples order from the left in each of the 3 membranes: MWM | CTRL1-1 | DOX1-1 | CTRL2-1 | DOX2-1 | CTRL1-2 | DOX1-2 | CTRL2-2 | DOX2-2

MWM: molecular weight marker

C/EBPβ - Blot

1

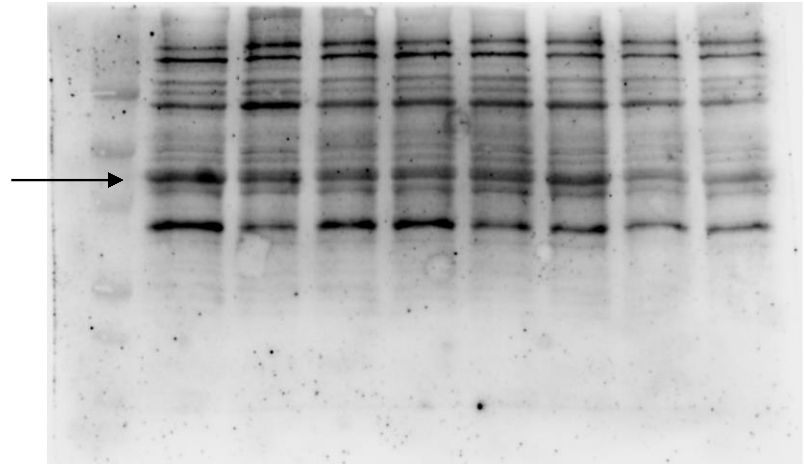

2

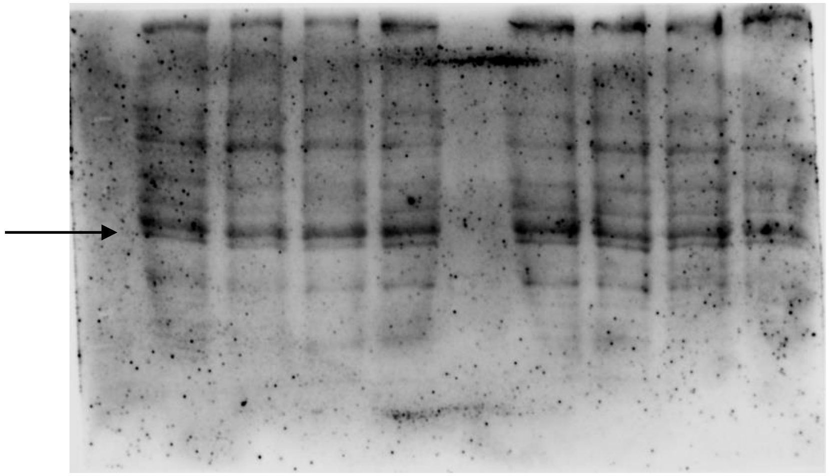

3

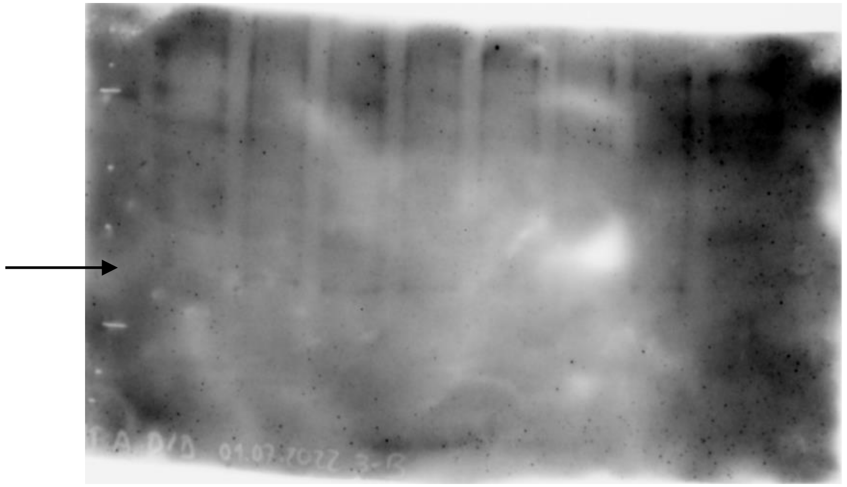

CITED4 - PonceauS

1

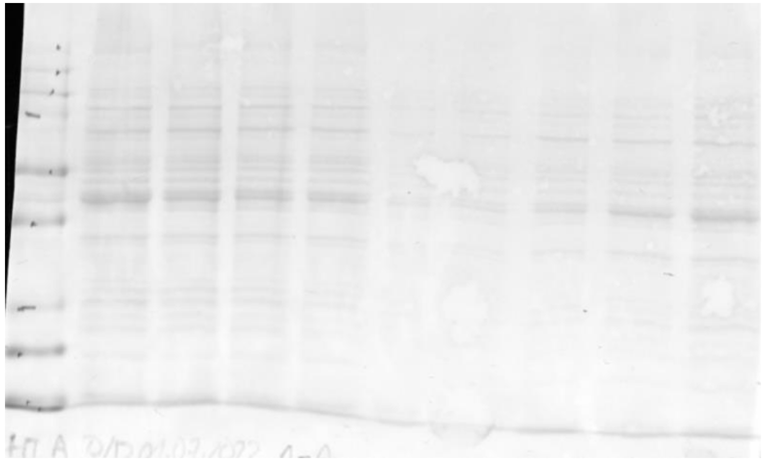

2

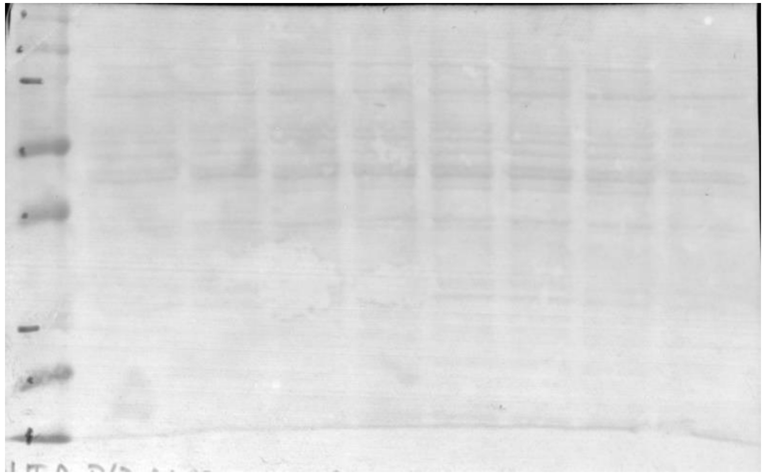

3

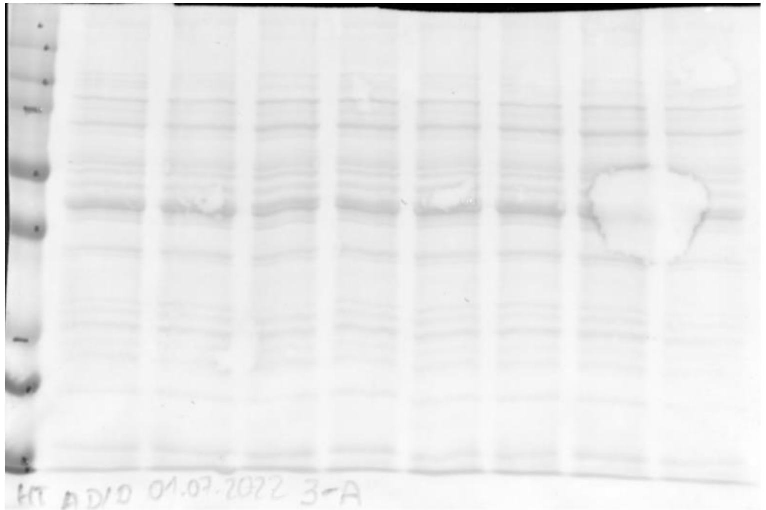

Samples order from the left in each of the 3 membranes: MWM | CTRL1-1 | DOX1-1 | CTRL2-1 | DOX2-1 | CTRL1-2 | DOX1-2 | CTRL2-2 | DOX2-2

MWM: molecular weight marker

CITED4 - Blot

1

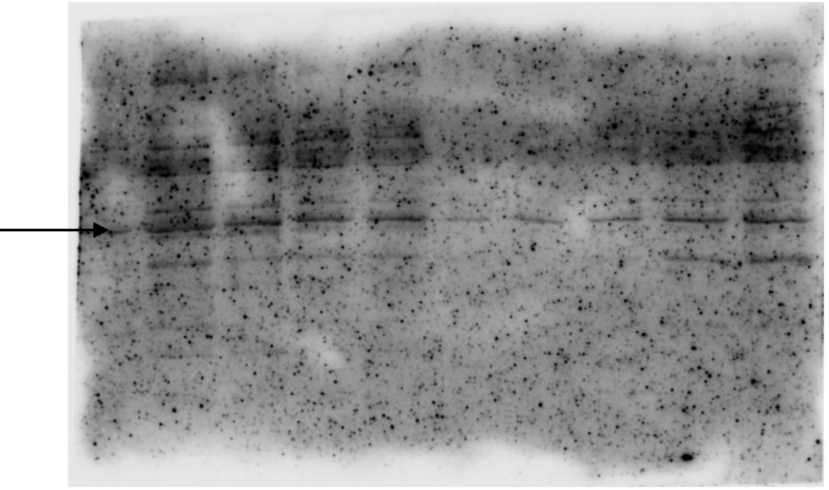

2

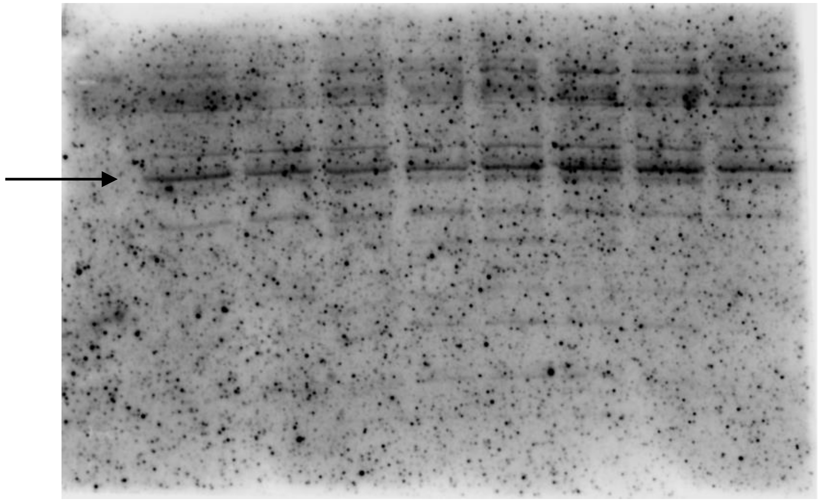

3

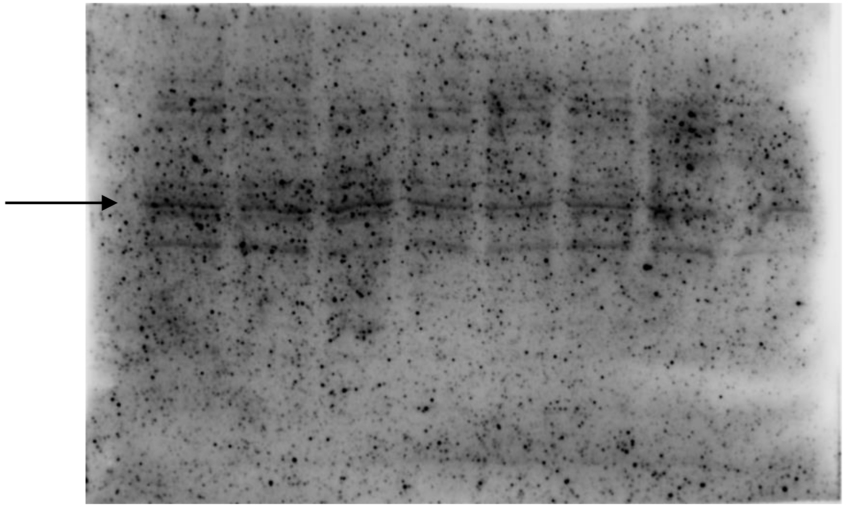

Supplement: Supplementary file 1 [file pharmaceuticals-16-01613-s001.zip › SuppFigureS1.pdf]
