# Supplementary material for: The Metabolic Fingerprint of Doxorubicin-Induced Cardiotoxicity in Male CD-1 Mice Fades Away with Time While Autophagy Increases
Source: Pharmaceuticals (Basel). 2023 Nov 15;16(11):1613. doi: 10.3390/ph16111613 (PMC10675798; doi:10.3390/ph16111613)
Supplement: Supplementary file 1 [file pharmaceuticals-16-01613-s001.zip › SuppTableS1.pdf]

Supplementary Table S1. Biomarkers found modulated after DOX administration for the two time-points selected. The assessment (morphometric/serum/cardiac tissue) and the biological/molecular pathway of each biomarker is indicated. No changing (=), upregulation (↑) and downregulation (↓) is indicated for each time-point considering the modulation found for DOX compared to CTRL group. The predisposition to increased (⤉) or decreased (⤈) values in DOX compared to CTRL group is also indicated.

Assessment	Biological pathway	***Biomarker***	first time-point (one week)	second time-point (five months)
**Morphometric**	biometric	*whole-body weight*	=	↓
**Morphometric**	biometric	*heart weight*	=	↓
**Morphometric**	biometric	*tibial length*	=	↓
**Morphometric**	biometric	*heart weigh-to-whole-body weight*	=	=
**Morphometric**	biometric	*heart weigh-to-tibial length*	=	=
**Serum**	systemic whole-body metabolism	*glucose*	=	=
**Serum**	system inflammation	*total protein*	=	=
**Serum**	system inflammation	*albumin*	⤈	=
**Serum**	system inflammation	*CRP*	=	⤈
**Serum**	system inflammation	*DNP*	=	=
**Serum**	system inflammation	*nitrotyrosine*	=	=
**Serum**	cardiac inflammation	*CK-MB*	=	=
**Cardiac tissue**	energetic metabolism	*GLUT4*	⤈	=
**Cardiac tissue**	energetic metabolism	*PFKM*	=	=

**Cardiac tissue**	energetic metabolism	*ETF-QO*	↑	=
**Cardiac tissue**	energetic metabolism	*ATPB*	⤉	=
**Cardiac tissue**	energetic metabolism	*SIRT3*	=	=
**Cardiac tissue**	energetic metabolism	*PPARα*	=	=
**Cardiac tissue**	energetic metabolism	*PPARγ*	=	=
**Cardiac tissue**	energetic metabolism	*GSK-3β*	=	=
**Cardiac tissue**	energetic metabolism	*AMPK*	↓	=
**Cardiac tissue**	energetic metabolism	*pAMPK*	=	=
**Cardiac tissue**	energetic metabolism	*pAMPK-to-AMPK*	=	=
**Cardiac tissue**	redox/oxidative stress status	*DNP*	=	=
**Cardiac tissue**	redox/oxidative stress status	*nitrotyrosine*	=	=
**Cardiac tissue**	redox/oxidative stress status	*MnSOD*	=	⤈
**Cardiac tissue**	mitochondrial density	*CS activity*	⤉	↓
**Cardiac tissue**	mitochondrial biogenesis	*PGC-1α*	=	=
**Cardiac tissue**	mitochondrial biogenesis	*Tfam*	=	=
**Cardiac tissue**	mitochondrial fusion	*Mfn1*	↓	=
**Cardiac tissue**	autophagy	*Beclin1*	=	↓
**Cardiac tissue**	autophagy	*ATG5*	=	↓
**Cardiac tissue**	autophagy	*LC3B*	=	⤈

**Cardiac tissue**	mitophagy	*Parkin*	=	=
**Cardiac tissue**	mitophagy/apoptosis	*BNIP3*	⤉	=
**Cardiac tissue**	apoptosis	*BAX*	↑	=
**Cardiac tissue**	apoptosis	*BCL2*	=	=
**Cardiac tissue**	apoptosis	*BAX-to-BCL2*	⤉	=
**Cardiac tissue**	autophagy/protein folding	*HSP27*	=	⤉
**Cardiac tissue**	autophagy/protein folding	*HSP70*	=	=
**Cardiac tissue**	regeneration	*SCFR*	=	=
**Cardiac tissue**	regeneration	*C/EBPβ*	=	=
**Cardiac tissue**	regeneration	*CITED4*	=	=
