# Supplementary material for: The Metabolic Fingerprint of Doxorubicin-Induced Cardiotoxicity in Male CD-1 Mice Fades Away with Time While Autophagy Increases
Source: Pharmaceuticals (Basel). 2023 Nov 15;16(11):1613. doi: 10.3390/ph16111613 (PMC10675798; doi:10.3390/ph16111613)
Supplement: Supplementary file 1 [file pharmaceuticals-16-01613-s001.zip › SuppTableS2.pdf]

Supplementary Table S2. Specification of the supplier, reference code, host species, purity, and dilutions for each primary antibody used as well as their application on serum (S) or cardiac (C) samples. Antibodies were diluted 1:200 to 1:1000 in 5% (w/v) nonfat dry milk prepared in TBS-T.

Primary antibody	Supplier	Reference code	Host	Purity	Dilution used	Sample
**AMPK**	Abcam (Cambridge, UK)	ab80039	mouse	monoclonal	1:1000	C
**ATG5**	Abcam	ab108327	rabbit	monoclonal	1:1000	C
**ATPB**	Abcam	ab14730	mouse	monoclonal	1:1000	C
**BAX**	Abcam	ab32503	rabbit	monoclonal	1:1000	C
**BCL2**	Santa Cruz Technologies (Dallas, Texas, USA)	sc-7382	mouse	monoclonal	1:200	C
**Beclin1**	Santa Cruz Technologies	sc-48341	mouse	monoclonal	1:1000	C
**BNIP3**	Abcam	ab10433	mouse	monoclonal	1:1000	C
**C/EBPβ**	Abcam	ab32358	rabbit	polyclonal	1:1000	C
**CITED4**	MyBioSource (San Diego, CA, USA)	MBS833529	rabbit	polyclonal	1:1000	C
**CRP**	Abcam	ab65842	rabbit	polyclonal	1:1000	S
**DNP**	Merck KGaA (Darmstadt, Germany)	MAB2223	mouse	monoclonal	1:1000	S/C
**ETF-QO**	Abcam	ab91508	rabbit	polyclonal	1:1000	C
**GLUT4**	Abcam	ab48547	mouse	monoclonal	1:1000 or 1:500	C

**GSK-3β**	Santa Cruz Technologies	sc-377213	mouse	monoclonal	1:500	C
**HSP27**	Santa Cruz Technologies	sc-9012	rabbit	polyclonal	1:500	C
**HSP70**	Stress gen (Farmingdale, NY, USA)	SPA-810	mouse	monoclonal	1:1000	C
**LC3B**	Sigma-Aldrich (St. Louis, MO, USA)	L7543	rabbit	not defined	1:1000	C
**Mfn1**	Santa Cruz Technologies	sc-166644	mouse	monoclonal	1:1000	C
**MnSOD**	Abcam	ab13533	rabbit	polyclonal	1:1000	C
**Nitrotyrosine**	Merck KGaA	MAB5404	mouse	monoclonal	1:1000	S/C
**pAMPK**	Abcam	ab23875	rabbit	polyclonal	1:1000	C
**Parkin**	Santa Cruz Technologies	sc-32282	mouse	monoclonal	1:1000 or 1:500	C
**PFKM**	Abcam	ab154804	rabbit	monoclonal	1:1000	C
**PGC-1α**	Abcam	ab191838	rabbit	polyclonal	1:1000	C
**PPARα**	Abcam	ab24509	rabbit	polyclonal	1:1000	C
**PPARγ**	Abcam	ab41928	mouse	monoclonal	1:1000 or 1:500	C
**SCFR**	Santa Cruz Technologies	sc-168	rabbit	polyclonal	1:1000	C
**SIRT3**	Santa Cruz Technologies	sc-49744	goat	polyclonal	1:1000	C
**Tfam**	Santa Cruz Technologies	sc-28200	rabbit	polyclonal	1:1000	C
